# Combined anti-PD-L1 and anti-VEGFR2 therapy promotes the antitumor immune response in GBM by reprogramming tumor microenvironment

**DOI:** 10.1038/s41420-025-02427-7

**Published:** 2025-04-03

**Authors:** Lin Yao, Hao Wang, Yongsheng Liu, Ming Feng, Yanyan Li, Zuopeng Su, Wen Li, Yun Xiong, Heyang Gao, Youxin Zhou

**Affiliations:** Neurosurgery & Brain and Nerve Research Laboratory, The First Affiliated Hospital of Soochow University, Suzhou, China

**Keywords:** Cancer, Immunology

## Abstract

Inhibitors of programmed cell death ligand 1 (PD-L1) and vascular endothelial growth factor receptor 2 (VEGFR2) are commonly used in the clinic, but they are beneficial for only a minority of glioblastoma multiforme (GBM) patients. GBM has significant immunosuppressive properties, and there are many immunosuppressive cells and dysfunctional effector T cells in the tumor microenvironment (TME), which is one of the important reasons for the failure of clinical treatment of GBM. Here, we have identified P21 activated kinase 4 (PAK4) as a pivotal immune suppressor in the TME. PAK4 is a threonine protein kinase, and PAK4 knockdown attenuates vascular abnormalities and promotes T-cell infiltration. In this study, our results showed that the expression of PAK4 was significantly downregulated after VEGFR2 knockdown. Next, we constructed a coculture system of CD8+ T cells and GBM cells. Our findings showed that combined anti-PD-L1 and anti-VEGFR2 therapy can regulate the TME and inhibit GBM cells' immune escape; overexpression of PAK4 can reverse this effect. Finally, we tested the combination therapy in mouse intracranial graft tumor models and found that combination therapy can prolong mouse survival. These findings suggest that anti-VEGFR2 therapy can downregulate PAK4, reprogram the TME by increasing cytotoxic CD8+ T cells infiltration and activation, and enhance the therapeutic effect of anti-PD-L1 therapy on GBM cells.

## Introduction

Glioblastoma multiforme (GBM) is the most common primary malignant tumor of the adult central nervous system [1]. At present, the standard treatment for GBM is surgery supplemented with postoperative synchronous radiotherapy and adjuvant chemotherapy, but the prognosis of patients is still poor, and the median survival time is about 8 months [2, 3]. In recent years, immunotherapy has been increasingly applied in clinical trials, of which the most notable class is immune checkpoint inhibitors (ICIs). ICIs targeting programmed cell death receptor 1 (PD-1) and programmed cell death ligand 1 (PD-L1) can regulate the body’s own immune response to exert antitumor effects. Clinical studies have shown that PD-1/PD-L1 inhibitor immunotherapy for high-grade gliomas, including GBM, does not significantly improve the median overall survival (mOS) of patients, and the complex tumor microenvironment (TME) in GBM may be the reason for the unsatisfactory treatment response of GBM [4, 5].

Angiogenesis is one of the main characteristics of the TME, and GBM neovasculature is disordered, which can cause insufficient blood perfusion between tissues, hypoxia, low pH, and high osmotic pressure in interstitial fluid [6]. These tumor blood vessels are twisted, tangled, enlarged, and unevenly distributed, affecting the endothelium and pericytes and leading to vascular leakage and insufficient secretion of cell adhesion molecules, which increases the difficulty of immune cell infiltration into the tumor and limits the effectiveness of immunotherapy [7–9]. In the TME, hypoxic cancer cells, neutrophils, and other cells secrete vascular endothelial growth factor A (VEGFA) acts through their cognate receptors on the endothelium and blocks the expression of adhesion molecules, preventing T-cell infiltration [8, 10, 11].

Bevacizumab, an anti-VEGF antibody, can be used as a first-line treatment for GBM. Bevacizumab improves progression-free survival (PFS) in patients but does not increase the overall survival (OS) rate [12, 13]. Some studies have shown that the use of VEGF/VEGFR2 inhibitors can temporarily normalize the tumor vasculature and increase the number of CD8+ T cells in tumors [14, 15]. Research has demonstrated a dose-dependent synergistic effect between antiangiogenic therapy and immune checkpoint blockade, providing important insights into the optimal combination strategy of immunotherapy and antiangiogenic targeted drugs [16].

P21 activated kinase 4 (PAK4) is a serine/threonine protein kinase that participates in multiple signaling pathways in the body. PAK4 is overexpressed in various types of cancers and plays an important role in tumor invasion and metastasis [17–20]. A study showed that PAK4 knockout induces adhesion protein re-expression in endothelial cells (ECs), reduces vascular abnormalities, improves T cell infiltration, and inhibits GBM growth in mice [21]. Antoni Ribas [22] reported that in mouse tumor models, PAK4 gene knockout improved the response to PD-1 blockade therapy and enhanced antitumor effects. Therefore, targeting VEGFR2 to downregulate PAK4 combined with anti-PD-L1 therapy may be a novel approach for GBM treatment.

## Results

### PD-L1 and VEGFR2 expression are higher in patients with high-grade glioma according to the World Health Organization (WHO) system than in those with low-grade glioma

**Fig. 1:**
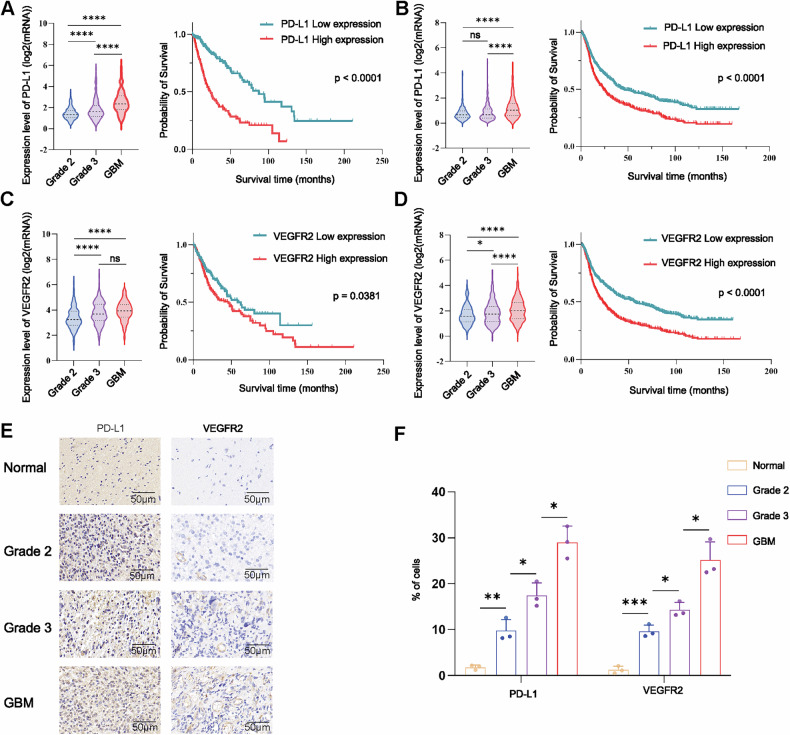
PD-L1 and VEGFR2 expression levels are high in high-grade gliomas and are inversely correlated with OS in glioma patients. Analysis of the TCGA (**A**) and CGGA (**B**) databases showed that PD-L1 expression was highest in GBM, and Kaplan‒Meier analysis indicated that glioma patients with low PD-L1 expression had better survival than those with high PD-L1 expression (*P* < 0.0001). The TCGA (**C**) and CGGA (**D**) databases showed that VEGFR2 was more highly expressed in high-grade gliomas than in low-grade gliomas, and Kaplan‒Meier analysis indicated that glioma patients with low VEGFR2 expression had better survival times than those with high VEGFR2 expression (*P* < 0.0001). **E**,**F** The protein expression levels of PD-L1 and VEGFR2 in normal and glioma tissues were detected by immunohistochemistry staining. Scale bar = 50 μm. ^ns^*P* > 0.05, *P < 0.05, **P < 0.01, ***P < 0.001, ****P < 0.0001, Student’s *t*-test.

Analysis of the The Cancer Genome Atlas (TCGA) database revealed that PD-L1 and VEGFR2 mRNA levels were greater in high-grade gliomas than in low-grade gliomas. In addition, in the TCGA cohort, Kaplan‒Meier analysis of PD-L1 and VEGFR2 expression indicated that patients with low PD-L1 and VEGFR2 expression had better survival, and similar results were obtained in the Chinese Glioma Genome Atlas (CGGA) database analysis (Fig. 1A‒D). Then, we examined the expression of PD-L1 and VEGFR2 in normal and glioma tissues of different WHO grades by immunohistochemistry. We found that these genes were more highly expressed in high-grade gliomas than in low-grade gliomas (Fig. 1E, F).

### Anti-PD-L1 and anti-VEGFR2 therapy promotes the toxic effect of immune cells in GBM cells

**Fig. 2:**
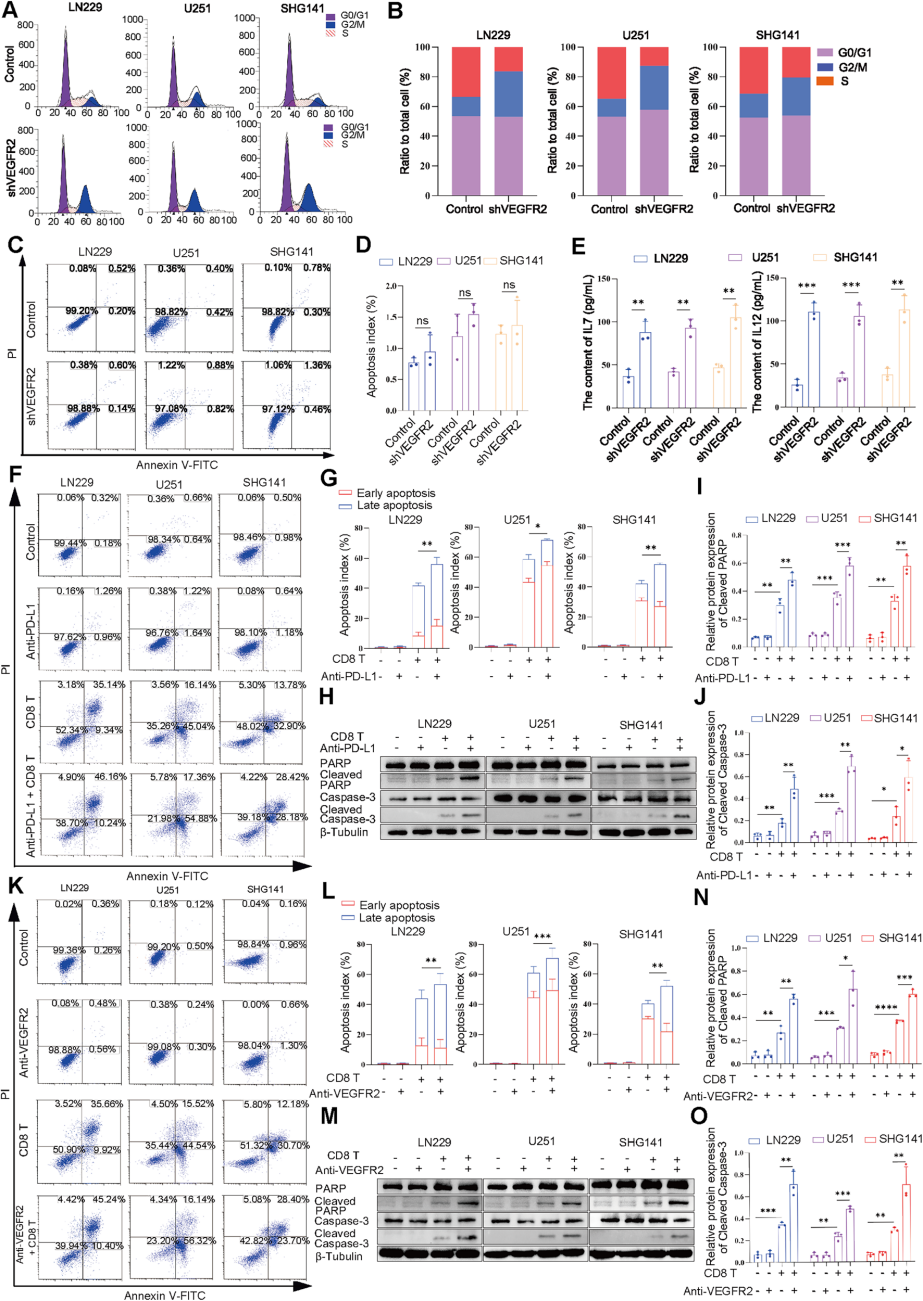
Anti-PD-L1 and anti-VEGFR2 therapy promotes antitumor immune response. **A**, **B** Flow cytometry showed that VEGFR2 knockdown induced cell cycle arrest in the G2-M phase on LN229, U251, and SHG141 cells. **C**, **D** Flow cytometry showed that VEGFR2 knockdown did not induce tumor cell apoptosis. **E** ELISA results showed that the concentrations of IL-7 and IL-12 were significantly greater in the VEGFR2 knockdown group than in the control group. **F**,**G**,**K**,**L** Flow cytometry analysis of GBM cells apoptosis in each treatment group. **H**–**J**, **M**–**O** The expression of cleaved PARP and cleaved caspase-3 in each treatment group was detected by western blotting. ^ns^*P* > 0.05, *P < 0.05, **P < 0.01, ***P < 0.001, ****P < 0.0001, Student’s *t*-test.

VEGFR2 expression in GBM cells promotes cell cycle progression [23]. To investigate the effect of VEGFR2 knockdown on the growth of GBM cells, we carried out cell cycle assays. We selected three GBM cell lines and used lentiviral transduction to knock down the VEGFR2 gene as the experimental group. The results showed that VEGFR2 knockdown causes cell cycle arrest in the G2/M phase (Fig. 2A, B). Next, we examined the apoptosis‐inducing effect of VEGFR2 knockdown in GBM cells by flow cytometry, and the results showed that VEGFR2 knockdown had no effect on GBM cells' apoptosis (Fig. 2C, D).

Next, we used Enzyme-linked immunosorbent assay (ELISA) to measure the levels of relevant cytokines. We found that the concentrations of interleukin-7 (IL-7) and interleukin-12 (IL-12) were significantly greater in the knockdown group (Fig. 2E). Research has shown that IL-7 and IL-12 play critical roles in maintaining lymphocyte homeostasis and have been suggested to reverse lymphopenia and improve the clinical outcomes of cancer patients, and recent studies showed that intratumoral dual expression of IL-7 and IL-12 increased activated CD8+ T cells in poorly immunogenic tumors [24–26].

Subsequently, we used flow cytometry to analyze the effect of anti-PD-L1 and anti-VEGFR2 agents on GBM cells' apoptosis. Anti-PD-L1 and anti-VEGFR2 therapy alone were added to culture medium containing only GBM cells, but could not induce the apoptosis of GBM cells. Next, to investigate the effect of anti-PD-L1 and anti-VEGFR2 therapy on the tumor immune response, we cocultured activated CD8+ T cells and GBM cells in medium treated with anti-PD-L1 or anti-VEGFR2 therapy, and results showed that both anti-PD-L1 and anti-VEGFR2 therapy induced GBM cells apoptosis (Fig. 2F, G, K, L). We further analyzed the expression of apoptosis proteins using western blotting and obtained similar results. Treatment with anti-PD-L1 and anti-VEGFR2 in the coculture system of activated CD8+ T cells and GBM cells resulted in a significant increase in cleaved poly ADP-ribose polymerase (PARP) and cleaved caspase-3 (Fig. 2H–J, M–O). These results suggest that anti-PD-L1 and anti-VEGFR2 therapy can promote the toxic effect of immune cells on GBM cells.

### VEGFR2 knockdown downregulates the expression of PAK4 and affects tumor microenvironment-related genes

**Fig. 3:**
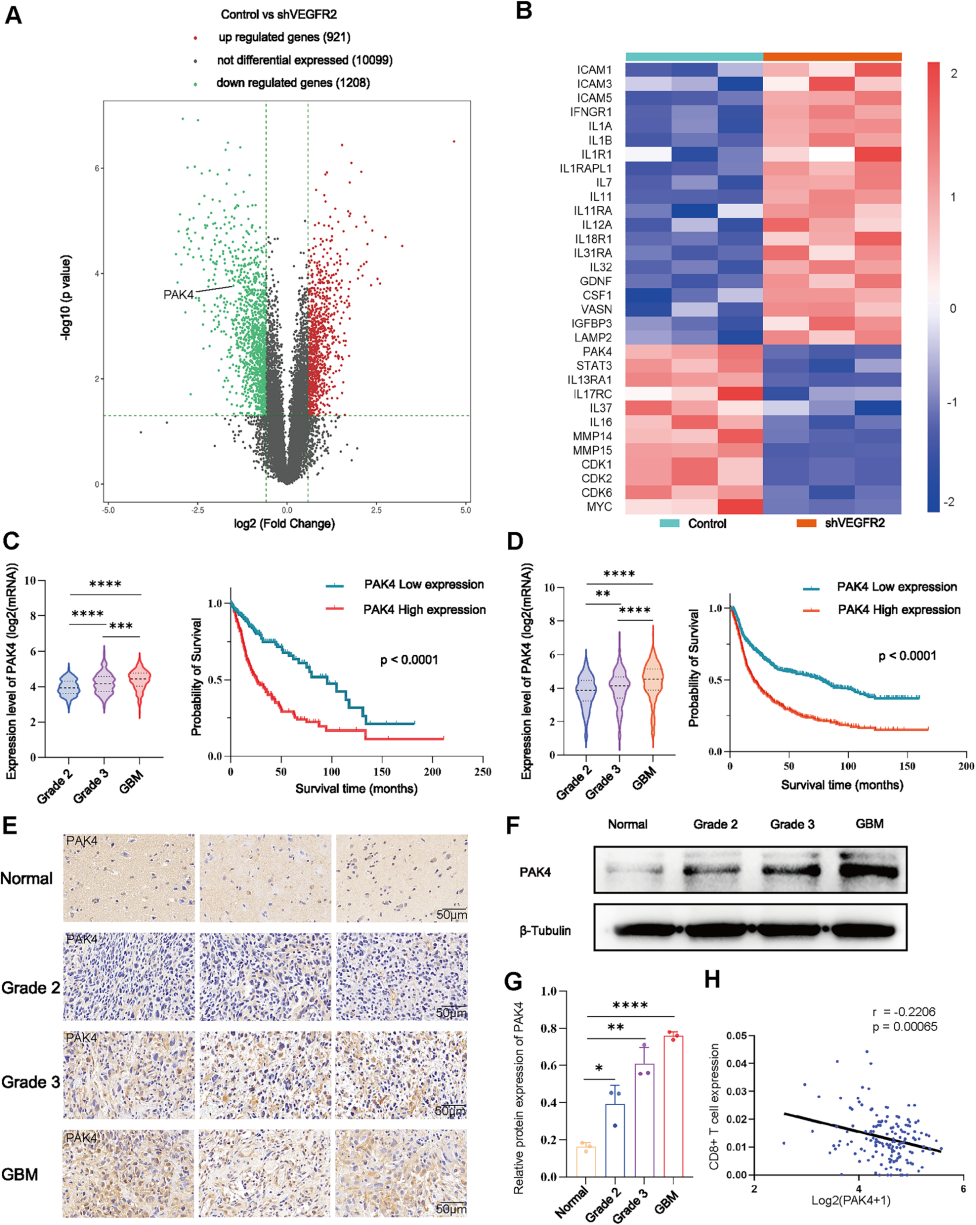
VEGFR2 in GBM regulates PAK4 and tumor microenvironment-related gene expression. **A** Volcano plot of the RNA-Seq results. Red dots indicate upregulated genes, green dots indicate downregulated genes, and black dots indicate genes without differential expression. **B** Heatmap of genes expressed in the VEGFR2 knockdown and control groups. **C, D** Analysis of the TCGA (**C**) and CGGA (**D**) databases showed that PAK4 was most highly expressed in GBM among different glioma grades. Kaplan‒Meier analysis indicated that glioma patients with low PAK4 expression had better survival times than those with high PAK4 expression. PAK4 protein expression in normal and glioma tissues was detected by immunohistochemistry (**E**) and western blotting (**F**, **G**). Scale bar = 50 μm. **H** The correlations between PAK4 expression and CD8+ T cells immune score were analyzed with Spearman. ^ns^*P* > 0.05, *P < 0.05, **P < 0.01, ***P < 0.001, ****P < 0.0001, Student’s *t*-test.

The RNA sequencing (RNA-seq) results showed that the expression levels of 921 genes were significantly increased, and those of 1208 genes were decreased after VEGFR2 knockdown (Fig. 3A). We used heatmaps to assess the expression of TME-related genes. VEGFR2 knockdown significantly downregulated PAK4 and upregulated TME-related genes such as IL-7, IL-12, and intercellular adhesion molecule-1 (ICAM-1) (Fig. 3B). Gene Ontology (GO) analysis of the downregulated genes revealed that the genes were enriched in the cell cycle (Supplemental Fig. 1A, B). Kyoto Encyclopedia of Genes and Genomes (KEGG) pathway analysis of the genes revealed similar results (Supplemental Fig. 1C, D). Moreover, GO analysis of the upregulated genes revealed enrichment in the response to stimulus and cell communication (Supplemental Fig. 2A, B). In addition, KEGG pathway analysis revealed that the tumor necrosis factor (TNF) signaling pathway was significantly enriched in upregulated genes (Supplemental Fig. 2C, D).

Subsequently, the results of the TCGA and CGGA database analyses showed that PAK4 mRNA levels were greater in high-grade gliomas, and Kaplan‒Meier analysis of PAK4 indicated that patients with low PAK4 expression had longer survival (Fig. 3C, D). The results of immunohistochemistry and western blotting showed that PAK4 was more highly expressed in glioma tissues than in normal tissues, and the expression of PAK4 protein was positively correlated with the WHO grade (Fig. 3E–G). The correlations between PAK4 expression and CD8+ T cells' immune score were analyzed with Spearman, and the result showed a negative correlation (*r* = −0.2206, *p* = 0.00065) (Fig. 3H).

### Knocking down VEGFR2 can reduce the binding between VEGFR2 and STAT3, decrease the generation of p-STAT3, and thereby inhibit the expression of PAK4

**Fig. 4:**
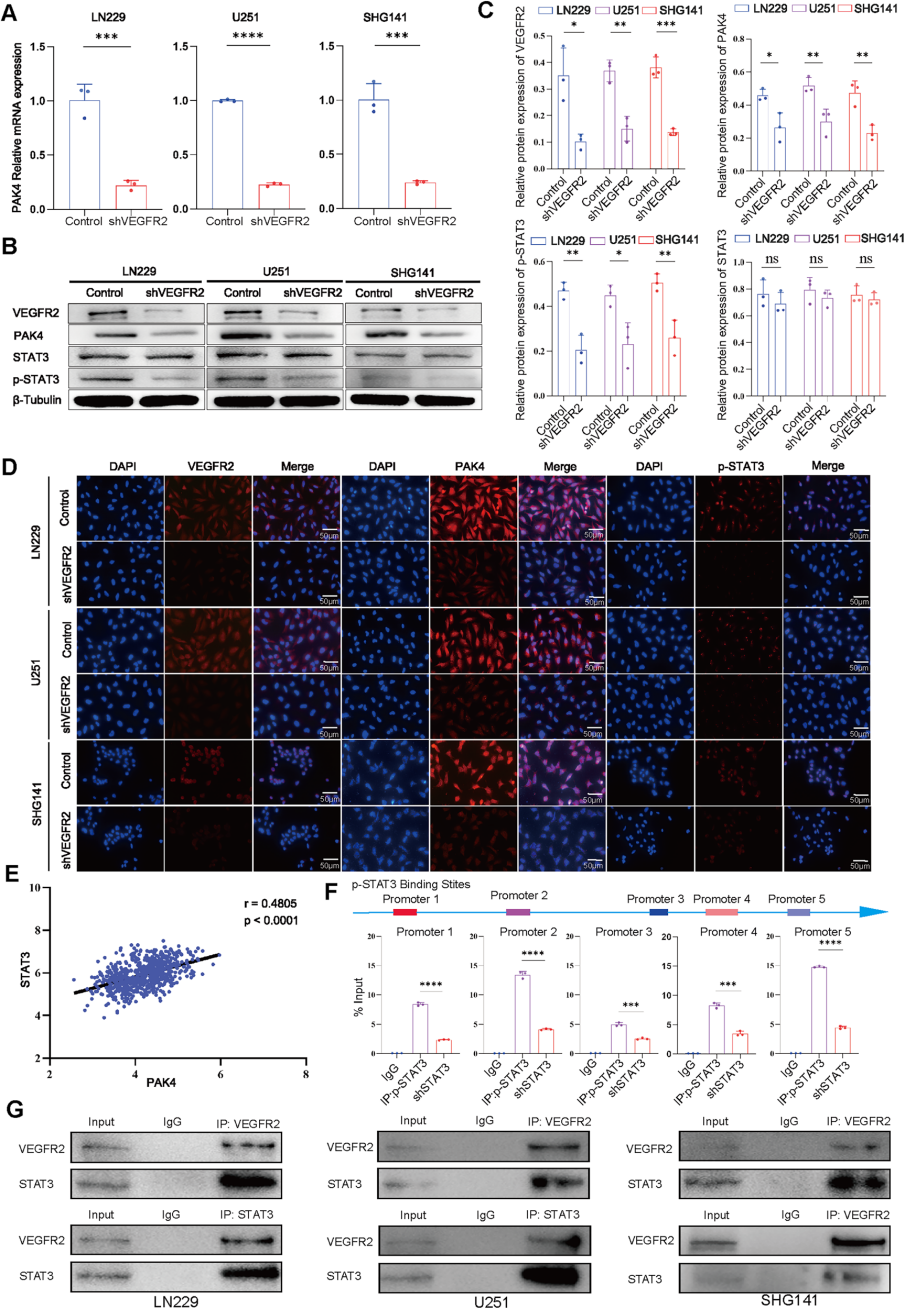
VEGFR2 binds to STAT3 and phosphorylates STAT3, thereby regulating the expression of PAK4. **A** qRT‒PCR was used to investigate the expression of PAK4 after VEGFR2 knockdown. Western blotting (**B**, **C**) and immunofluorescence staining (**D**) showing the expression of VEGFR2, PAK4, STAT3, and p-STAT3 after VEGFR2 knockdown. Scale bar = 50 μm. **E** The TCGA database was used to determine the correlation between the expression profiles of PAK4 and STAT3. **F** Potential promoter binding sites and ChIP‒qPCR data showing the binding of p-STAT3 to the PAK4 gene. IgG was used as a reference. **G** Co-IP assays demonstrated that VEGFR2 can interact with STAT3. ^ns^*P* > 0.05, *P < 0.05, **P < 0.01, ***P < 0.001, ****P < 0.0001, Student’s *t*-test.

To explore the underlying mechanism, real-time quantitative reverse transcription PCR (qRT‒PCR) analysis revealed that VEGFR2 knockdown significantly downregulated the expression of PAK4 (Fig. 4A) in the GBM cell lines. RNA-seq revealed that after VEGFR2 knockdown, the expression of PAK4 was significantly downregulated, and the signal transducer and activator of transcription 3 (STAT3) was downregulated. STAT3 is involved in regulating cell growth, proliferation, and apoptosis, and its sustained activation is closely related to the occurrence, development, and treatment resistance of GBM [27, 28]. Studies have suggested that STAT3 suppresses CD8+ T cells' activity in the TME [29, 30]. Next, we further investigated the expression levels of related genes after VEGFR2 knockdown at the protein level in GBM cells. As shown by western blotting and immunofluorescence staining, after VEGFR2 knockdown, the expression of VEGFR2, PAK4, and phosphorylated STAT3 (p-STAT3), but not that of STAT3, was significantly decreased (Fig. 4B–D). Next, we treated GBM cells with anti-PD-L1 and anti-VEGFR2 for 48 h. We observed that anti-VEGFR2 therapy alone significantly downregulated the expression of PAK4 and p-STAT3, and the expression of STAT3 remained unchanged. After anti-PD-L1 therapy alone, the gene expression of PAK4, STAT3, and p-STAT3 did not decrease. Meanwhile, after combination therapy, the expression of PAK4 and p-STAT3 did not further decrease compared to anti-VEGFR2 therapy alone (Supplemental Fig.3A–D). Bioinformatics predictions indicated that PAK4 is highly positively correlated with STAT3 (*r* = 0.4805), and there may be an interaction between PAK4 and STAT3 (Fig. 4E). Bioinformatics predictions indicated that p-STAT3 has multiple binding sites in the regulatory region of the PAK4 gene. To confirm binding at these sites, chromatin immunoprecipitation followed by quantitative PCR (ChIP‒qPCR) was performed in LN229 cells. The results showed that p-STAT3 binding sites were spread across the PAK4 promoter region, and PAK4 expression significantly decreased after STAT3 knockdown (Fig. 4F). These studies provide a mechanistic explanation for the regulation of PAK4 expression by p-STAT3. We further confirmed the interaction between VEGFR2 and STAT3. Coimmunoprecipitation (Co-IP) revealed that the antibody against VEGFR2 was able to pull down STAT3 in GBM cells, and the antibody against STAT3 could also pull down VEGFR2, confirming direct binding between VEGFR2 and STAT3 (Fig. 4G).

### PAK4 levels in GBM affect the antitumor immune response

**Fig. 5:**
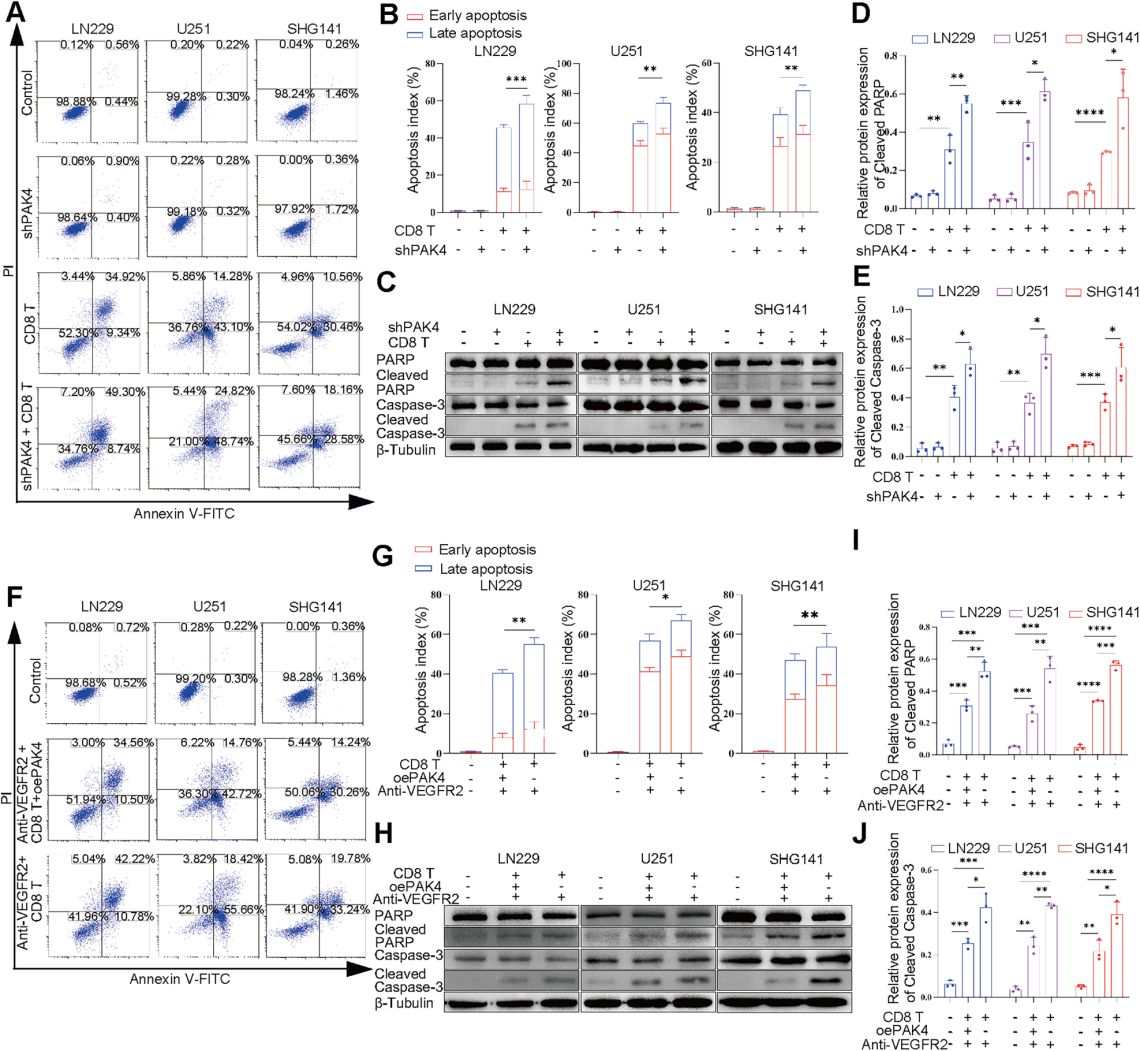
PAK4 affects the antitumor immune response. **A**, **B**, **F**, **G** Flow cytometry assays showing the apoptosis of GBM cells in the control and experimental groups. **C**–**E**, **H**–**J** The expression of cleaved PARP and cleaved caspase-3 in the control and experimental groups was detected by western blotting. ^ns^*P* > 0.05, *P < 0.05, **P < 0.01, ***P < 0.001, ****P < 0.0001, Student’s t test.

PAK4 was knocked down in GBM cells via shRNA lentiviral transduction. After conducting flow cytometry, we found that PAK4 knockdown did not induce the apoptosis of GBM cells. Next, we cocultured PAK4 knockdown GBM cells with activated CD8+ T cells for 48 h. To our surprise, we observed that PAK4 knockdown induced apoptosis in GBM cells, leading to a significant increase in the percentage of apoptotic cells compared to that in the control group of GBM cells cocultured with CD8+ T cells (Fig. 5A, B). The western blot results showed that PAK4 knockdown in GBM cells resulted in a significant increase in cleaved PARP and cleaved caspase-3 levels in the coculture system (Fig. 5C–E).

Next, we used lentiviral transduction to overexpress PAK4 in GBM cells. In the coculture system of activated CD8+ T cells and GBM cells, the flow cytometry results indicated that PAK4 overexpression can reduce apoptosis induced by anti-VEGFR2 therapy (Fig. 5F, G). Subsequently, we analyzed the proportion of apoptotic cells using western blot and obtained similar results (Fig. 5H–J). Overall, these findings suggest that PAK4 levels in GBM affect the toxicity of immune cells in GBM cells and the immune escape of GBM cells.

### Anti-PD-L1 and anti-VEGFR2 therapy can increase the secretion of IFN-γ, GZMB, and perforin by CD8+ T cells and can decrease exhaustion markers in vitro

**Fig. 6:**
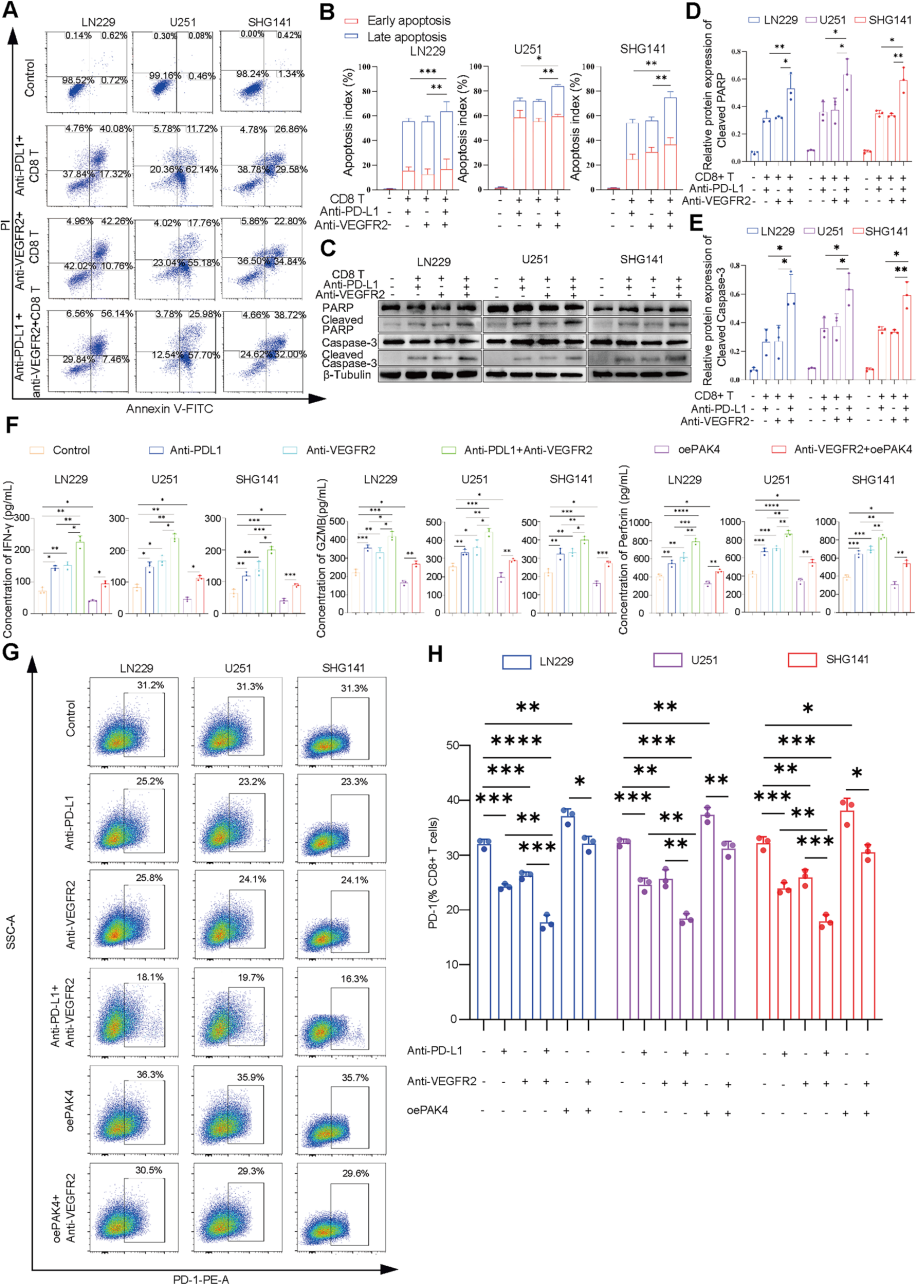
Anti-PD-L1 and anti-VEGFR2 therapy can increase the secretion of cytokines by CD8+ T cells. **A**, **B** Flow cytometry assay showing the apoptosis of GBM cells in the control and experimental groups. **C**–**E** The expression of cleaved PARP and cleaved caspase-3 in the control and experimental groups was detected by western blotting. **F**–**H** Activated CD8+ T cells were cocultured with GBM cells at a 1:2 ratio, and were treated with anti-PD-L1/anti-VEGFR2 for 48 h. ELISA was used to evaluate the levels of IFN-γ, GZMB, and perforin secreted by CD8+ T cells in the control and experimental groups (**F**). Flow cytometry assay showed the expression of PD-1 on CD8+ T cells in the control and experimental groups (**G**, **H**). ^ns^*P* > 0.05, *P < 0.05, **P < 0.01, ***P < 0.001, ****P < 0.0001, Student’s *t*-test.

In this study, we cocultured CD8+ T cells and GBM cells in medium with both anti-PD-L1 and anti-VEGFR2 therapy, and flow cytometry showed that combination therapy with anti-PD-L1 and anti-VEGFR2 could promote the apoptosis of GBM cells to a greater degree than therapy with either agent alone (Fig. 6A, B), and western blot analysis yielded similar results (Fig. 6C–E). Next, the ELISA results showed that the expression levels of interferon-γ (IFN-γ), granzyme B (GZMB), and perforin increased after anti-PD-L1 or anti-VEGFR2 therapy, and their expression increased further when the anti-PD-L1 and anti-VEGFR2 therapies were combined. To further investigate the impact of PAK4 on the immune function of CD8+ T cells, we conducted coculture experiments using PAK4-overexpressing GBM cells and CD8+ T cells. The levels of IFN-γ, GZMB, and perforin were decreased in the PAK4 overexpression group compared with the control group, and anti-VEGFR2 therapy reversed these changes (Fig. 6F). Subsequently, flow cytometry was conducted to investigate the impact of anti-PD-L1/anti-VEGFR2 therapy on the immune function of CD8+ T cells. The results showed that PD-1 on CD8+ T cells was significantly decreased in anti-PD-L1 or anti-VEGFR2 therapy group, and PD-1 expression decreased further when the anti-PD-L1 and anti-VEGFR2 therapies were combined. Meanwhile, PD-1 on CD8+ T cells was increased in the PAK4 overexpression group compared with the control group, and anti-VEGFR2 therapy reversed these changes (Fig. 6G, H). This finding suggested that anti-PD-L1 and anti-VEGFR2 therapy increases the secretion of IFN-γ, GZMB, and perforin by T-cell and reduces T-cell exhaustion to promote the cytotoxic effects of CD8+ T cells.

### Combination therapy with anti-PD-L1 and anti-VEGFR2 agents prevents tumor growth in an intracranial tumor model

**Fig. 7:**
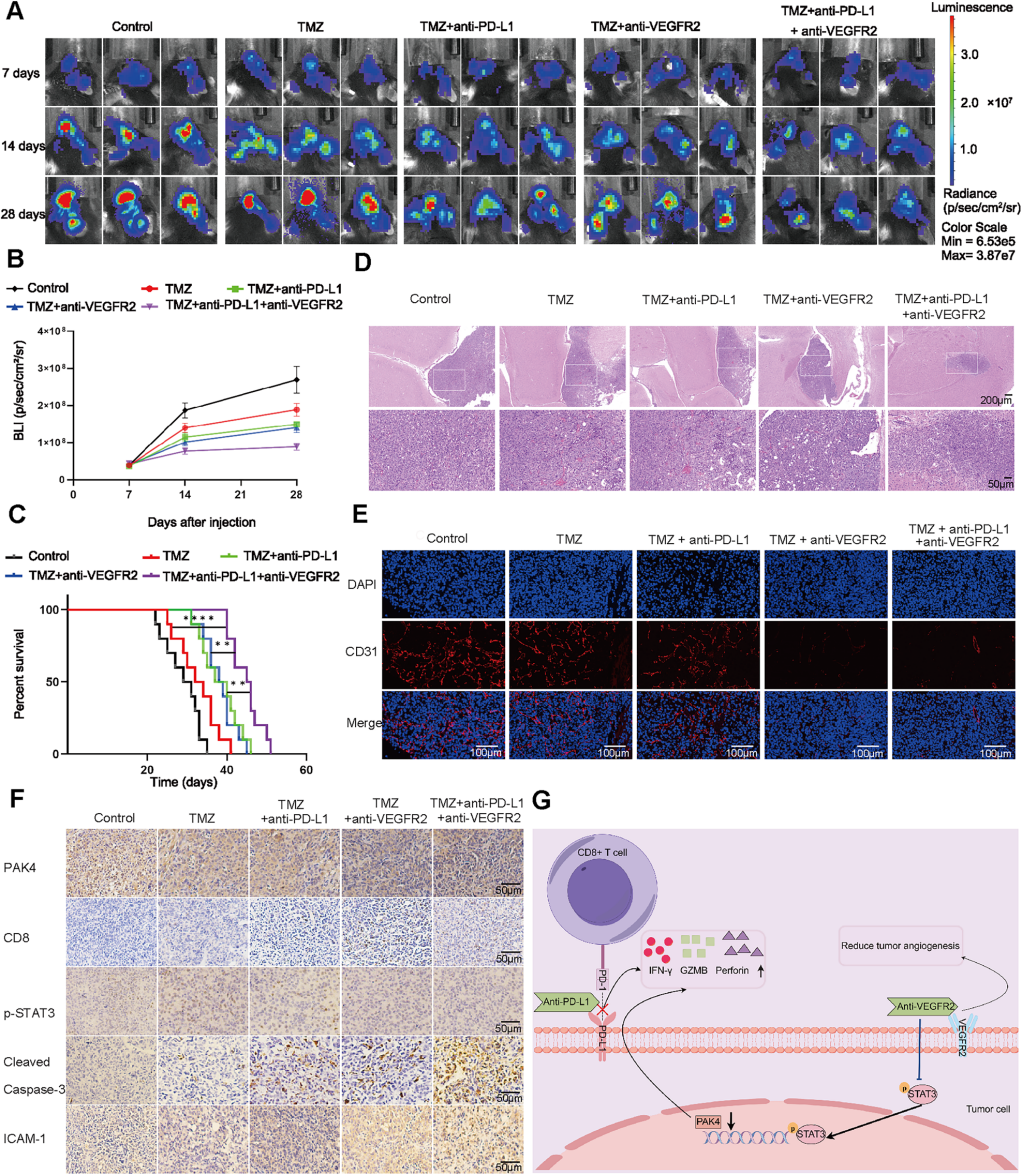
Combination therapy suppresses GBM tumor growth in intracranial tumor models. **A** Representative fluorescence images of mice in the control and experimental groups on days 7, 14, and 28. **B** Quantitative analysis of fluorescence images. **C** The overall survival of mice in the control and experimental groups. **D** Representative images of H&E-stained tumor sections from mice. Scale bar = 200 μm, enlarged scale bar = 50 μm. **E** Representative images of immunofluorescence staining of tumor sections from mice. Scale bar = 100 μm. **F** Representative images of immunohistochemistry staining of mouse tumor sections. Scale bar = 50 μm. ^ns^*P* > 0.05, *P < 0.05, **P < 0.01, ***P < 0.001, ****P < 0.0001, Student’s *t*-test. **G** Mechanistic diagram showing that anti-VEGFR2 therapy in GBM can downregulate PAK4, increase cytotoxic CD8+ T cells infiltration and activation, and enhance the therapeutic effect of anti-PD-L1 therapy.

To investigate the impact of anti-PD-L1 and anti-VEGFR2 therapy on intracranial transplanted tumors in mice, GL261 cells were injected into the intracranial region of mice to establish in situ models. The size of the intracranial tumors and the survival time of the mice were monitored through in vivo imaging. The results showed that anti-PD-L1 and anti-VEGFR2 therapy significantly suppressed the growth of intracranial tumors in mice and prolonged their survival time, and combination therapy displayed better efficacy than monotherapy (Fig. 7A–C). Subsequent hematoxylin and eosin (H&E) staining of the mouse brain revealed a significant decrease in the growth of intracranial transplanted tumors in the combined treatment group (Fig. 7D). Immunofluorescence analysis revealed a significant reduction in CD31 expression after anti-VEGFR2 therapy (Fig. 7E). The immunohistochemistry showed that anti-VEGFR2 therapy decreased the expression levels of PAK4, p-STAT3, and ICAM-1. In addition, we examined apoptosis marker and CD8 marker levels in situ models by immunohistochemistry analysis, and the results showed that combination therapy significantly increased the expression levels of cleaved caspase-3 and CD8 (Fig. 7F). These findings suggest that combination therapy may change the TME and enhance the killing effect of intracranial CD8+ T cells on intracranially transplanted tumor cells.

In conclusion, we demonstrated that anti-VEGFR2 therapy could reduce the expression of PAK4 and elucidated the underlying molecular mechanisms involved. We also demonstrated that anti-PD-L1 and anti-VEGFR2 therapy can increase the secretion of IFN-γ, GZMB, and perforin by immune cells and reprogram the TME of GBM, thereby promoting the cytotoxic effects of CD8+ T cells (Fig. 7G).

## Discussion

The treatment strategies for GBM include surgery, radiotherapy, chemotherapy, targeted therapy, and immunotherapy [3, 31]. GBM is immunologically cold and tends to lack T-cell infiltration, which is one of the important reasons for the failure of clinical treatment for GBM. For a long time, the clinical treatment efficacy of GBM has been poor, and treatment with PD-L1 immunotherapy to activate the antitumor immune response has only been beneficial for a subset of patients. Groot et al. [4] demonstrated that PD-L1 monotherapy alone cannot induce an effector immunological response in most GBM patients, probably owing to a scarcity of T-cell in the TME and low levels of PD-1 and PD-L1.

Bevacizumab can affect vascular permeability, proliferation, endothelial cell migration, and survival to inhibit tumor angiogenesis, growth, and metastasis [32]. Notably, VEGF can promote angiogenesis by reducing ICAM-1 expression and disrupting the interaction between leukocytes and endothelial cells, thereby hindering the infiltration of immune effector T-cells into tumors [8]. Studies have shown that antiangiogenic drugs can promote tumor vascular normalization by antagonizing VEGF signaling, helping to relieve T-cell immunosuppression and promote T-cell accumulation in tumors; moreover, antiangiogenic drugs can also block VEGF-mediated suppression of dendritic cell functions, which can effectively positively regulate the immune function of the body [33, 34]. Unfortunately, the use of bevacizumab in clinical treatment did not significantly prolong the survival time of newly diagnosed GBM patients, and anti-VEGFR2 therapy alone often has only short-term effects, which may be closely related to excessive vascular pruning and lack of T-cells in the TME [12, 35]. In this study, we used VEGFR2 inhibitors on GBM cells in vitro and found that anti-VEGFR2 therapy did not induce apoptosis in GBM cells. Subsequently, we established a coculture system of activated CD8+ T cells and GBM cells, and the western blotting and flow cytometry results showed that anti-VEGFR2 therapy promotes the toxic effect of immune cells on GBM cells. So, we speculate that anti-VEGFR2 therapy can regulate the TME, thereby promoting T-cell cytotoxicity against GBM cells.

The TME is composed of tumor cells, the extracellular matrix (ECM), blood vessels, immune cells, cytokines, and other factors [36]. In this study, RNA-Seq and immunohistochemistry revealed that the expression of ICAM-1 increased after VEGFR2 knockdown or anti-VEGFR2 therapy. ICAM-1 is an integrin ligand that regulates leukocyte-endothelial interactions and plays a crucial role in the structure of the tumor vasculature and T-cell trafficking into tumors [37]. Our findings indicated that anti-VEGFR2 can modulate the TME by impacting cytokine release and expression of TME-related genes. In all, this part of the research provides a basis for subsequent immunotherapy research. To further explore the specific molecular mechanism by which VEGFR2 knockdown is involved in regulating the TME and promoting immune cell-induced GBM cells apoptosis, we screened the target gene PAK4 based on RNA-Seq results and bioinformatics analysis.

PAK4 reportedly regulates many cellular functions, such as adhesion, migration, proliferation, and immune regulation; moreover, PAK4 can contribute to abnormal tumor vascularization and hypoxia in cancer [38, 39]. Divya Kesanakurti [19] reported that PAK4 upregulation in gliomas and further determined its role in mesenchymal transition and radioresistance. Leon J [40] reported that PAK4 is a putative target for radiosensitization and impairing DNA repair in GBM. Fan, Y [21]. reported that PAK4 knockdown can increase T-cell infiltration and inhibit the growth of tumors derived from human GBM cells transplanted into animal models. In this study, western blotting, immunofluorescence, and immunohistochemistry were used to verify that VEGFR2 knockdown could downregulate the expression of PAK4 and p-STAT3. In addition, we used Co-IP and ChIP-qPCR to reveal that VEGFR2 binds and phosphorylates STAT3, and then involved in PAK4 transcription.

Recently, immunotherapy combined with targeted therapy has shown great therapeutic potential, as many immunosuppressive cells and dysfunctional effector T cells are present in the TME. The combined use of ICIs and antiangiogenic drugs can significantly prolong the therapeutic window of tumor vascular normalization, while vascular normalization attenuates immunosuppression in the TME, increases the infiltration of T-cell, and ultimately promotes tumor regression [41]. Shigeta et al. [42]. showed that dual anti-PD-1/VEGFR2 therapy has a durable vessel fortification effect on hepatocellular carcinoma (HCC) and can overcome treatment resistance to either treatment alone, and increase overall survival in HCC models by influencing the TME. To investigate the efficacy of combination therapy in the treatment of GBM in this study, we established a coculture system and assessed the apoptosis of GBM cells through western blotting and flow cytometry. We further detected the levels of IFN-γ, GZMB, and perforin in the medium by ELISA, and detected the changes in the expression of exhaustion-associated markers by flow cytometry. The study revealed that the combination treatment yielded outcomes superior to those of anti-PD-1/anti-VEGFR2 therapy alone. The results also showed that PAK4 overexpression can inhibit immune cell-induced GBM cells apoptosis in the coculture system and can lead to CD8+ T cell dysfunction. We speculate that anti-VEGFR2 therapy can downregulate PAK4, reprogram the GBM TME, and increase immune cell infiltration, thereby enhancing the therapeutic effect of anti-PD-L1 therapy on GBM cells. Finally, we tested the combination therapy in orthotopic mouse models of GBM and found that the combination therapy prolonged mouse survival.

Drug resistance is a common problem with single-target antitumor therapy; combination therapy can solve this problem, and the presence of inhibitory metabolites in the TME can prevent T-cells from attacking cancer cells. Additionally, the upregulation of the expression of other immune checkpoint molecules due to compensation can promote tumor immune escape, leading to poor efficacy of monotherapy. Combination therapy has been shown to “heat up” cold tumors and increase the response rate to anti-PD-L1 therapy. Therefore, combination therapy holds great potential for promoting the recognition and killing of tumor cells by the immune system. TMZ is a first-line chemotherapy drug for GBM, but the overall survival of GBM patients remains poor. Combined anti-PD-L1 and anti-VEGFR2 therapy is associated with ‘normalization’ of the TME and has the potential to improve the efficacy of TMZ and is a promising therapeutic partner in a combination regimen in GBM patients.

### Limitations of the study

There are two major limitations in this study that could be addressed in future research. First, a detailed understanding of the mechanistic effects of PAK4 regulating CD8+ T cells is required. Specifically, it is necessary to investigate how PAK4 regulates CD8+ T cells in the GBM TME, thereby mediating immune evasion in GBM cells. Second, we need to study the effects of anti-VEGFR2 therapy on other cells in the GBM TME, such as neutrophils, rather than just focusing on GBM cells.

## Conclusions

In conclusion, we elucidated the underlying molecular mechanism by which VEGFR2 binds and phosphorylates STAT3 and subsequently affects PAK4 transcription. Our findings showed that combined anti-PD-L1 and anti-VEGFR2 therapy increased CD8+ cytotoxic T-cell infiltration and that combination therapy was effective in GBM models. Overall, our research provides a scientific and objective theoretical foundation for the clinical utilization of antiangiogenic medications and immunotherapy in the treatment of GBM.

## Materials and methods

### Brain tissue specimens and cell culture lines

Human GBM and brain contusion tissue samples were obtained from the First Affiliated Hospital of Soochow University, Suzhou, China. This study was approved by the Ethics Committee of Soochow University (Approval No. SUDA20221206H03). We used 3 human GBM cell lines, LN229 and U251, which were obtained from the Shanghai Institutes for Biological Sciences. The primary cell line SHG141, which was established in our laboratory, was obtained from the tumor tissue of a GBM patient at the First Affiliated Hospital of Soochow University. The murine glioma cell line GL261 was obtained from the American Type Culture Collection (ATCC). The cells were cultured in DMEM (Gibco, Waltham, MA, USA) supplemented with 10% fetal bovine serum (FBS) and 1% penicillin‒streptomycin (Gibco).

### Bioinformatics analysis

Standardized mRNA expression datasets for glioma from TCGA and CGGA were downloaded to evaluate the expression of PD-L1, VEGFR2, and PAK4 transcripts. Detailed information on all glioma patients, including pathological diagnosis, clinical stage, and survival data, was also downloaded from the TCGA and CGGA databases. The RNA-sequencing expression profiles and corresponding clinical information for GBM were downloaded from the TCGA dataset. The R software ggstatsplot package was used to draw the correlations between gene expression and CD8+ T cells' immune score.

### Antibodies

Anti-VEGFR2 (CST #2479), anti-PD-L1 (CST #13684), anti-p-STAT3 (CST #9145), anti-STAT3 (CST #12640), anti-β-tubulin (CST #2146), anti-cleaved PARP (CST #5625), anti-PARP (CST #9542), anti-cleaved caspase-3 (CST #9664), anti-caspase-3 (CST #14220), anti-CD8 (CST #98941), and anti-rabbit IgG (CST #2729) antibodies were purchased from CST (Danvers, MA, USA); anti-PAK4 (Affinity #DF7078) and anti-CD31 (Affinity #AF6191) antibodies were purchased from Affinity Biosciences (Cincinnati, OH, USA).

### Immunohistochemistry

The tumor tissue sections were deparaffinized in xylene and rehydrated with graded concentrations of ethanol and distilled water. Then, 0.3% hydrogen peroxide was used to quench the endogenous peroxidase activity, and the strong antigen recovery solution was heated to 37 °C to recover the antigen. Nonspecific proteins were blocked with 5% goat serum (Solarbio Life Science, Beijing, China). The primary antibody was incubated at 4 °C overnight, and the secondary antibody was incubated at room temperature for 60 min. Then, the slides were incubated with ABC peroxidase and diaminobenzidine (ZSGBBio, Beijing, China) and counterstained with Mayer hematoxylin solution (Solarbio, Beijing, China) for nuclear staining.

### Lentiviral transfection and drug treatment

Lentiviral shRNAs against the VEGFR2, PAK4, and STAT3 genes and PAK4 overexpression vector were produced by GeneChem (China) (Supplementary Table S1). Following the manufacturer’s recommendation, the constructed lentiviral vector was transfected into GBM cells, infection was achieved using 100 pmol lentiviral vector and 5 μL Lipofectamine 3000 in 2 × 10^5^ cells/mL. After transfection for 24 h, the infected cells were subsequently treated with puromycin. Then, we investigated the infected cells.

In a cell-based assay, we treated GBM cells with anti-PD-L1 (5.0 µg/mL, MSB2311) and anti-VEGFR2 (5.0 µg/mL, MSB0254) synthesized by Mabspace Biosciences (Suzhou, China) Co., Ltd, and assessed the effects after 48 h of treatment.

### Western blotting

After the cell protein samples were extracted, a BCA (Beyotime, Shanghai, China) kit was used to measure the protein concentration, which was adjusted so that it was equal for all samples; loading buffer was added at a 1:3 ratio for high-temperature (100 °C) denaturing for 5 min. The proteins were separated by 8%–12% SDS‒PAGE, and the proteins were transferred to polyvinylidene fluoride (PVDF) membranes. The membranes were incubated with primary antibody overnight and then incubated with secondary antibody at 37 °C for 1 h.

### Flow cytometric analysis

For the cell cycle, GBM cells and shVEGFR2 GBM cells were inoculated in a 6-well plate. Then, the cells were fixed in 70% ethanol overnight at 4 °C and labeled with 0.5 mL/test PI/RNAse staining buffer (BD Biosciences, San Jose, CA, USA, cat #550825) for 30 min in the dark. The cells were assessed on a Beckman Coulter FC500 (Beckman Coulter, Inc., CA, USA) and analyzed with ModFit LT (version 5, Verity Software House, Inc., Topsham, ME, USA).

To assess cell apoptosis, an apoptosis detection kit (BD Biosciences, San Jose, CA, USA) was used according to the manufacturer’s protocol. GBM cells in different experimental groups were collected and then suspended in Annexin V-FITC and PI binding buffer. The cells were assessed on a Beckman Coulter FC500 and analyzed with Kaluza Analysis 2.1 software (Beckman Coulter).

For functional analysis of CD8+ T cells, the T-cells were collected, washed with cell staining buffer (eBioscience), then centrifuged at 300 × *g* for 5 min. Subsequently, the T-cells were stained with the indicated antibodies. Antibodies: FITC anti-human CD8 (eBioscience); PE anti-human PD-1 (eBioscience).

### Enzyme-linked immunosorbent assay (ELISA)

The GBM cells and shVEGFR2 GBM cells were cultured separately for 48 h, the culture medium was collected. According to the manufacturer’s instructions, ELISA kits were used to measure the concentrations of IL-7 (Thermo Fisher, EHIL7) and IL-12 (Thermo Fisher, BMS238) in the obtained medium. In the coculture system, activated CD8+ T cells were collected and cocultured with GBM cells at a 1:2 ratio, and were placed in a 24-well culture plate for 48 h. At the end of coculture, culture supernatants were collected after experimental treatment. ELISA kits were used to measure the concentrations of IFN-γ (Thermo Fisher, KHC4021), GZMB (Thermo Fisher, BMS2027-2), and perforin (Thermo Fisher, BMS2306) in the obtained medium.

### Immunofluorescence

The cells were fixed with 4% paraformaldehyde, permeabilized with 0.5% Triton, and blocked with 5% BSA for 30 min. Subsequently, the membranes were incubated with the primary antibody at 4 °C overnight. The next day, the cells were washed three times with PBS for 15 min each, incubated with the fluorescent secondary antibody at room temperature for 1 h, and washed three times with PBS for 5 min each. Finally, the nuclei were labeled with DAPI, and then the cells were observed under a fluorescence microscope.

Paraffin sections of the brain were dried and sequentially immersed in xylene, 95% ethanol, and 90% ethanol twice for 5 min each. Then, the sections were placed in hot sodium citrate, boiled for 5 min, and cooled to room temperature with cold sodium citrate. Next, the sections were incubated in 0.2% Triton for 15 min and then blocked with 5% bovine serum albumin (BSA) for 1 h. The sections were incubated with the primary antibody at 4 °C overnight and with a fluorescent secondary antibody at 37 °C for 1 h. Finally, the specimens were sealed with DAPI, and images were acquired via a fluorescence microscope.

### CD8+ T cells isolation and coculture experiments

CD8+ T cells were isolated from fresh peripheral blood mononuclear cells (PBMCs) collected from healthy volunteers. The research protocol was approved by the Ethics Committee of Soochow University (Approval No. SUDA20221206H03). The blood was diluted with PBS 1:1, and was added into a 15 mL separation tube, and the tube was centrifuged at 400 × *g* for 30 min at room temperature; then, the upper plasma was removed before proceeding. PBMCs were collected in a new centrifuge tube, resuspended, washed with PBS, and centrifuged at 400 × *g* for 10 min at room temperature, and this was repeated twice. We used magnetic beads (Miltenyi Biotec, Germany) to sort live CD8+ monocytes from the obtained PBMCs. The obtained cells were cultured in RPMI 1640 (Gibco, USA) medium. Then, we used complete RPMI 1640 medium supplemented with 10% FBS and 10 IU/mL interleukin-2 (IL-2) (PeproTech) to cultivate CD8+ T cells in vitro and activated them with CD3/CD28 Dynabeads (Thermo Fisher, 11456D).

Activated CD8+ T cells were collected and cocultured with GBM cells at a 1:2 ratio. After 48 h of coculture, the CD8+ T cells and GBM cells were separately collected for flow cytometry (Gallios, Beckman Coulter).

### Coimmunoprecipitation (Co-IP)

After cell lysates were obtained, the samples were processed using a homogenizer, followed by centrifugation at 4 °C at 12,000 × *g* for 15 min to collect the supernatant. To reduce non-specific binding of proteins, the supernatant was incubated with protein A/G beads and centrifuged at 3000 × *g* for 1 h at 4 °C, and the supernatant was collected. After which, the supernatant was incubated with protein A/G beads and anti-VEGFR2, anti-STAT3, IgG overnight in a cold room. The next day, the sample was centrifuged at 3000 × *g* for 15 min at 4 °C. The immunoprecipitates were washed three times using western blotting and IP cell lysis buffer, resuspended in sample loading buffer, and heated at 100 °C for 5 min. Finally, the samples were centrifuged to obtain the supernatant, which was subjected to further western blotting.

### Quantitative reverse transcription polymerase chain reaction (qRT‒PCR)

Total RNA was extracted from the cells using the Trizol assay, and the RevertAid First Strand cDNA Synthesis Kit (Fermentas; Thermo Fisher Scientific) was used to synthesize complementary DNA (cDNA). According to the manufacturer’s instructions, cDNA aliquots were subjected to qRT‒PCR. The sequences of primers used for the detection of PAK4 mRNA were as follows:

5′-ATCTGGTCGCTGGGGATAATG-3′ (forward) and

5′-CAGGTTGTCCCGAATCATCTTC-3′ (reverse).

### Chromatin immunoprecipitation followed by quantitative PCR (ChIP-qPCR)

Briefly, LN229 cell and shSTAT3 LN229 cell lysates were subjected to crosslinking in 1% formaldehyde at 37 °C for 10 min, and the reaction was terminated with glycine (125 mM) for 5 min. Then, a Bioruptor (Diagenode) was used for chromatin fragmentation, and the samples were incubated overnight with antibody. The samples were divided into three aliquots for qPCR analysis. The Ct value of each ChIP DNA fragment was standardized to the Ct value of the input DNA fragment detected in the same qPCR assay (ΔCt) to illustrate the difference in chromatin sample preparation. The %Input for each ChIP fraction was calculated as 2ˆ [Ct (input) – Ct (ChIP)] × Fd × 100% (Fd is the input dilution factor, equal to 100/5 = 20), and anti-IgG fold enrichment was evaluated. The Supplementary Table S2 showed the binding site of p-STAT3 on the promoter region of PAK4.

### Animal experiment

The animal experiment was approved by the Animal Experiment Ethics Committee of Soochow University (Approval No. SUDA20221206A02). GL261 cells were used to establish a model of intracranial tumor in C57BL/6J mice. 8-week-old female C57BL/6J mice were purchased from the Shanghai Experimental Animal Center of the Chinese Academy of Sciences (Shanghai, China). GL261 cells (5 × 10^5^) were stereotactically injected into the mice. The mice were randomly divided into five groups with 10 mice in each group. Seven days after cell inoculation, the mice were treated with drugs. To closely simulate clinical administration, drug experimental groups of mice were injected with 20 mg/kg temozolomide (TMZ) every 3 days. The mice in the first group were injected intraperitoneally with physiological saline as a control group. The mice in the second group were intraperitoneally injected with TMZ. The mice in the third group were intraperitoneally injected with TMZ and anti-mouse PD-L1 (10 mg/kg, twice a week; Bio X cell, #BE0101). The mice in the fourth group were intraperitoneally injected with TMZ combined with anti-mouse VEGFR2 (10 mg/kg, once a week; Mabspace Biosciences, MSB0254m). The mice in the fifth group were intraperitoneally injected with anti-PD-L1, anti-VEGFR2, and TMZ. On days 7, 14, and 28, intracranial tumor size was assessed using an IVIS spectral real-time imaging system (Blandford, USA). The animal research was conducted in accordance with internationally recognized norms and national regulations. Mouse brains were removed, fixed in 4% paraformaldehyde, and embedded in paraffin for H&E staining, immunohistochemistry, and immunofluorescence.

### Hematoxylin and eosin

For H&E staining, the slides were deparaffinized, rehydrated, and counterstained using an H&E kit (Solarbio Life Science, Beijing, China) after nuclear staining. Images were acquired using an inverted microscope (Olympus, Tokyo, Japan).

### Statistical analysis

All experimental data in this study were analyzed using GraphPad Prism 8.0 (GraphPad Software Inc., San Diego, CA, USA) and were replicated at least three times. For most experiments, Student’s *t*-test was used for statistical analysis. One-way analysis of variance followed by Tukey’s post hoc test was used to assess the differences between the groups. Kaplan–Meier survival analysis was used to assess mouse survival time. The significance of differences according to the p value is indicated as follows: ^ns^*P* > 0.05, *p < 0.05, **p < 0.01, ***p < 0.001, and ****p < 0.0001.

## Supplementary information

Supplementary Table S1

Supplemental Table S2

Supplemental legends

Supplemental Fig 1

Supplemental Fig 2

Supplemental Fig 3

Original WB

## Data Availability

All data generated or analyzed during this study are included in this published article or available from the corresponding author on reasonable request.
