# Supplementary material for: Combined anti-PD-L1 and anti-VEGFR2 therapy promotes the antitumor immune response in GBM by reprogramming tumor microenvironment
Source: Cell Death Discov. 2025 Apr 3;11:136. doi: 10.1038/s41420-025-02427-7 (PMC11968841; doi:10.1038/s41420-025-02427-7)
Supplement: Supplementary file 1 — Supplementary Table S1 [file 41420_2025_2427_MOESM1_ESM.docx]

**Supplementary Table S1** The detail gene shRNA sequence

| Gene | Sequence |
| --- | --- |
| shVEGFR2 (5’ to 3’) | AGGCTAATACAACTCTTCAAA |
| shPAK4-1 (5’ to 3’) | GCGAGTATCCCATGAGCAGTT |
| shPAK4-2 (5’ to 3’) | GCGAGTATCCCATGAGCAGTT |
| shPAK4-3 (5’ to 3’) | GGGTGAAGCTGTCAGACTTTG |
| oePAK4 (5’ to 3’) | ATGTTTGGGAAGAGGAAGAAGCGGGTGGAGATCTCCGCGCCGTCCAACTTCGAGCACCGCGTGCACACGGGCTTCGACCAGCACGAGCAGAAGTTCACGGGGCTGCCCCGCCAGTGGCAGAGCCTGATCGAGGAGTCGGCTCGCCGGCCCAAGCCCCTCGTCGACCCCGCCTGCATCACCTCCATCCAGCCCGGGGCCCCCAAGACCATCGTGCGGGGCAGCAAAGGTGCCAAAGATGGGGCCCTCACGCTGCTGCTGGACGAGTTTGAGAACATGTCGGTGACACGCTCCAACTCCCTGCGGAGAGACAGCCCGCCGCCGCCCGCCCGTGCCCGCCAGGAAAATGGGATGCCAGAGGAGCCGGCCACCACGGCCAGAGGGGGCCCAGGGAAGGCAGGCAGCCGAGGCCGGTTCGCCGGTCACAGCGAGGCGGGTGGCGGCAGTGGTGACAGGCGACGGGCGGGGCCAGAGAAGAGGCCCAAGTCTTCCAGGGAGGGCTCAGGGGGTCCCCAGGAGTCCTCCCGGGACAAACGCCCCCTCTCCGGGCCTGATGTCGGCACCCCCCAGCCTGCTGGTCTGGCCAGTGGGGCGAAACTGGCAGCTGGCCGGCCCTTTAACACCTACCCGAGGGCTGACACGGACCACCCATCCCGGGGTGCCCAGGGGGAGCCTCATGACGTGGCCCCTAACGGGCCATCAGCGGGGGGCCTGGCCATCCCCCAGTCCTCCTCCTCCTCCTCCCGGCCTCCCACCCGAGCCCGAGGTGCCCCCAGCCCTGGAGTGCTGGGACCCCACGCCTCAGAGCCCCAGCTGGCCCCTCCAGCCTGCACCCCCGCCGCCCCTGCTGTTCCTGGGCCCCCTGGCCCCCGCTCACCACAGCGGGAGCCACAGCGAGTATCCCATGAGCAGTTCCGGGCTGCCCTGCAGCTGGTGGTGGACCCAGGCGACCCCCGCTCCTACCTGGACAACTTCATCAAGATTGGCGAGGGCTCCACGGGCATCGTGTGCATCGCCACCGTGCGCAGCTCGGGCAAGCTGGTGGCCGTCAAGAAGATGGACCTGCGCAAGCAGCAGAGGCGCGAGCTGCTCTTCAACGAGGTGGTAATCATGAGGGACTACCAGCACGAGAATGTGGTGGAGATGTACAACAGCTACCTGGTGGGGGACGAGCTCTGGGTGGTCATGGAGTTCCTGGAAGGAGGCGCCCTCACCGACATCGTCACCCACACCAGGATGAACGAGGAGCAGATCGCGGCCGTGTGCCTTGCAGTGCTGCAGGCCCTGTCGGTGCTCCACGCCCAGGGCGTCATCCACCGGGACATCAAGAGCGACTCGATCCTGCTGACCCATGATGGCAGGGTGAAGCTGTCAGACTTTGGGTTCTGCGCCCAGGTGAGCAAGGAAGTGCCCCGAAGGAAGTCGCTGGTCGGCACGCCCTACTGGATGGCCCCAGAGCTCATCTCCCGCCTTCCCTACGGGCCAGAGGTAGACATCTGGTCGCTGGGGATAATGGTGATTGAGATGGTGGACGGAGAGCCCCCCTACTTCAACGAGCCACCCCTCAAAGCCATGAAGATGATTCGGGACAACCTGCCACCCCGACTGAAGAACCTGCACAAGGTGTCGCCATCCCTGAAGGGCTTCCTGGACCGCCTGCTGGTGCGAGACCCTGCCCAGCGGGCCACGGCAGCCGAGCTGCTGAAGCACCCATTCCTGGCCAAGGCAGGGCCGCCTGCCAGCATCGTGCCCCTCATGCGCCAGAACCGCACCAGATGA |
| shSTAT3(5’ to 3’) | GCCATTGGCCGGAATTAGCGAACGGT |
