# Supplementary material for: Combined anti-PD-L1 and anti-VEGFR2 therapy promotes the antitumor immune response in GBM by reprogramming tumor microenvironment
Source: Cell Death Discov. 2025 Apr 3;11:136. doi: 10.1038/s41420-025-02427-7 (PMC11968841; doi:10.1038/s41420-025-02427-7)
Supplement: Supplementary file 2 — Supplemental Table S2 [file 41420_2025_2427_MOESM2_ESM.docx]

**Supplementary Table S2** The binding site of p-STAT3 on the promoter region of PAK4

| **Gene** | **Rrimer sequence** | **product length (bp)** |
| --- | --- | --- |
| PAK4-1 | F:5’ GAGGAAAGAAAGAAGGAAGGAA 3’  R:5’ GCTGGGACTATAGGCATAAGC3’ | 112 |
| PAK4-2 | F:5’ GAGACAACCAACAAGCAATAACTAC3’  R:5’ ATTCCCTCGCTCACCCTAAG3’ | 116 |
| PAK4-3 | F:5’ AGCGAAACTCTATCTCAAGACAAA3’  R:5’ ATTGAAAACAAACAGCAGACATC3’ | 87 |
| PAK4-4 | F:5’ GGGTTGTATATGCAGCGAGAA3’  R:5’ TTCGTGTGATACCGATTTTCTAAC3’ | 157 |
| PAK4-5 | F:5’ GGATGTTCGTTGGGGATTCA3’  R:5’ GCCTAACTGCTCGACCTGGA3’ | 109 |
