# Supplementary material for: Combined anti-PD-L1 and anti-VEGFR2 therapy promotes the antitumor immune response in GBM by reprogramming tumor microenvironment
Source: Cell Death Discov. 2025 Apr 3;11:136. doi: 10.1038/s41420-025-02427-7 (PMC11968841; doi:10.1038/s41420-025-02427-7)
Supplement: Supplementary file 3 — Supplemental legends [file 41420_2025_2427_MOESM3_ESM.docx]

**Supplemental Fig 1 A, B** Gene Ontology analysis of downregulated genes after VEGFR2 knockdown. **C, D** KEGG pathway analysis of downregulated genes after VEGFR2 knockdown.

**Supplemental Fig 2 A, B** Gene Ontology analysis of genes upregulated after VEGFR2 knockdown. **C, D** KEGG pathway analysis of genes upregulated after VEGFR2 knockdown.

**Supplemental Fig 3** The expression of PAK4, STAT3, p-STAT3 in each treatment group was detected by western blotting.
