# Supplementary figures and images for: Combined anti-PD-L1 and anti-VEGFR2 therapy promotes the antitumor immune response in GBM by reprogramming tumor microenvironment
Source: Cell Death Discov. 2025 Apr 3;11:136. doi: 10.1038/s41420-025-02427-7 (PMC11968841; doi:10.1038/s41420-025-02427-7)

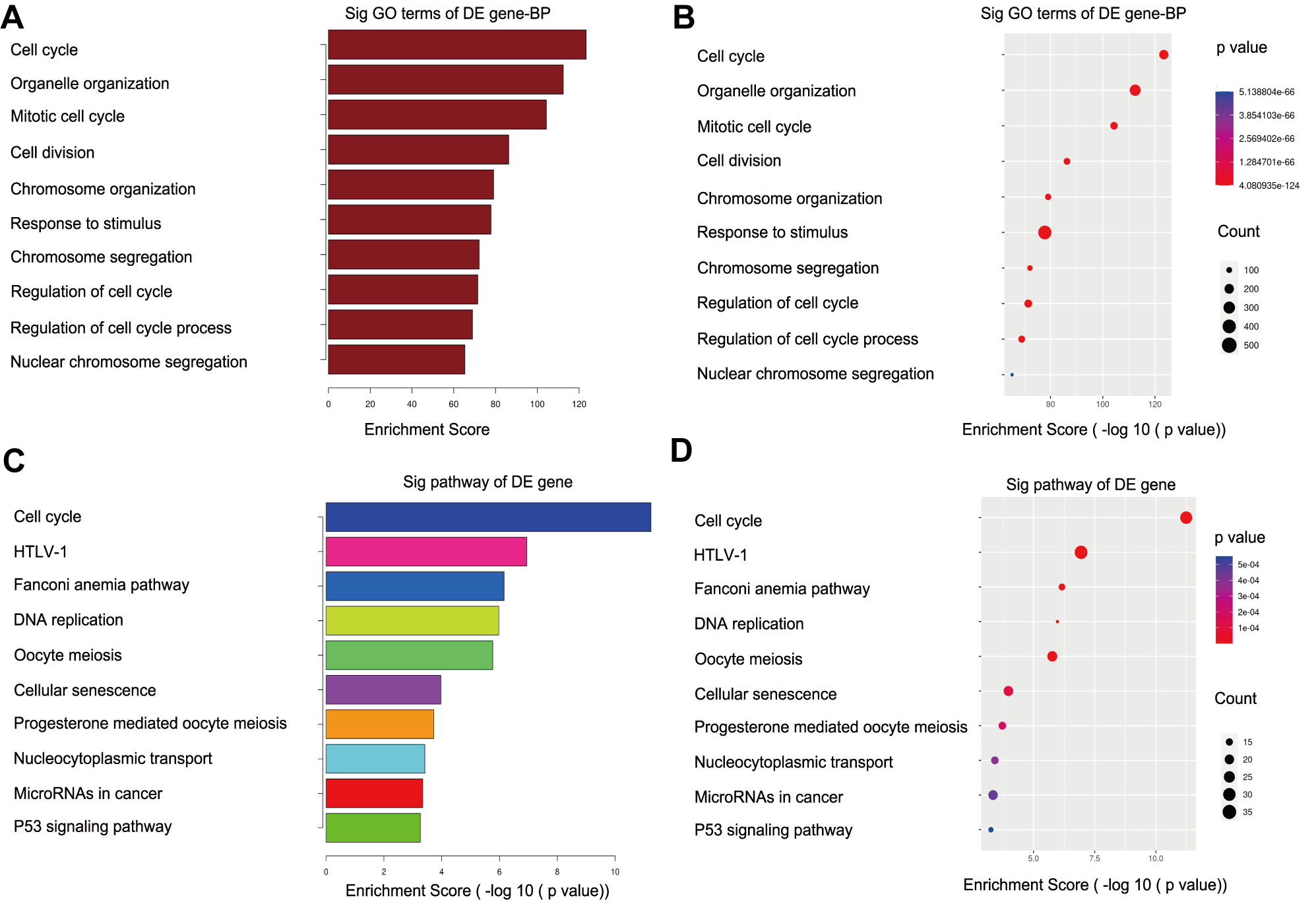

Supplement: Supplementary file 4 — Supplemental Fig 1 [file 41420_2025_2427_MOESM4_ESM.tif]

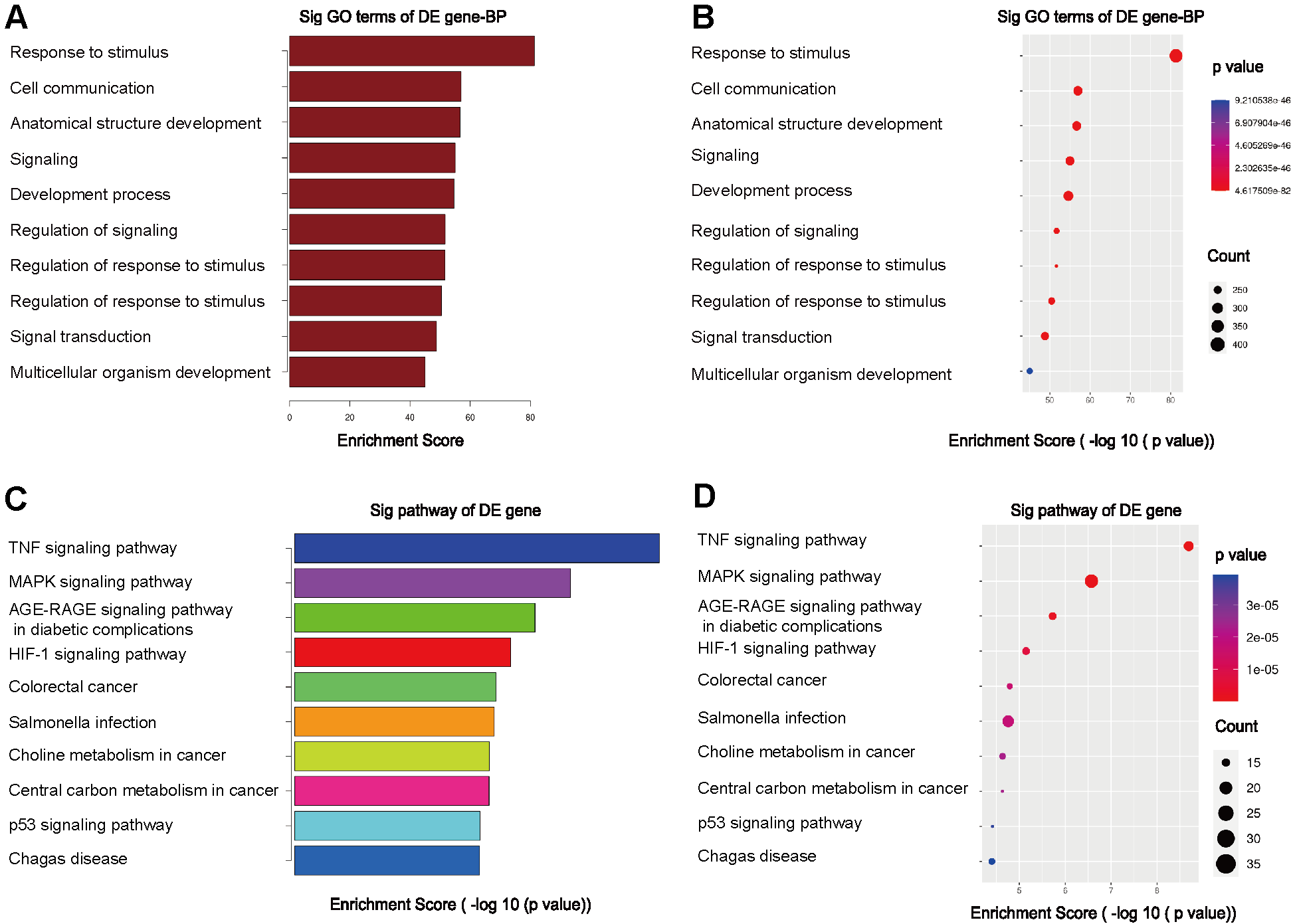

Supplement: Supplementary file 5 — Supplemental Fig 2 [file 41420_2025_2427_MOESM5_ESM.tif]

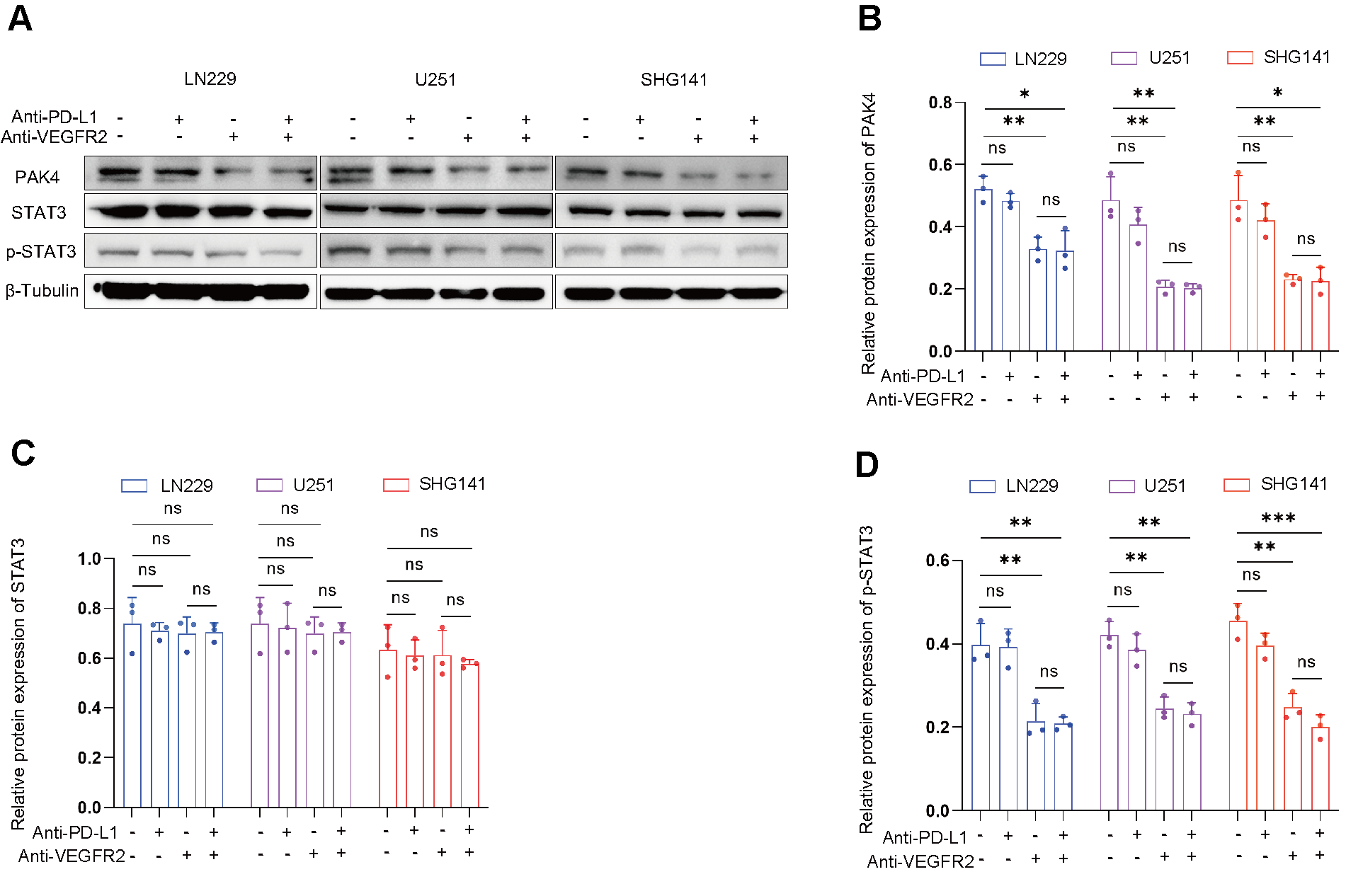

Supplement: Supplementary file 6 — Supplemental Fig 3 [file 41420_2025_2427_MOESM6_ESM.tif]
