# Supplementary material for: Combined anti-PD-L1 and anti-VEGFR2 therapy promotes the antitumor immune response in GBM by reprogramming tumor microenvironment
Source: Cell Death Discov. 2025 Apr 3;11:136. doi: 10.1038/s41420-025-02427-7 (PMC11968841; doi:10.1038/s41420-025-02427-7)
Supplement: Supplementary file 7 — Original WB [file 41420_2025_2427_MOESM7_ESM.pdf]

Fig 2H LN229 tubulin

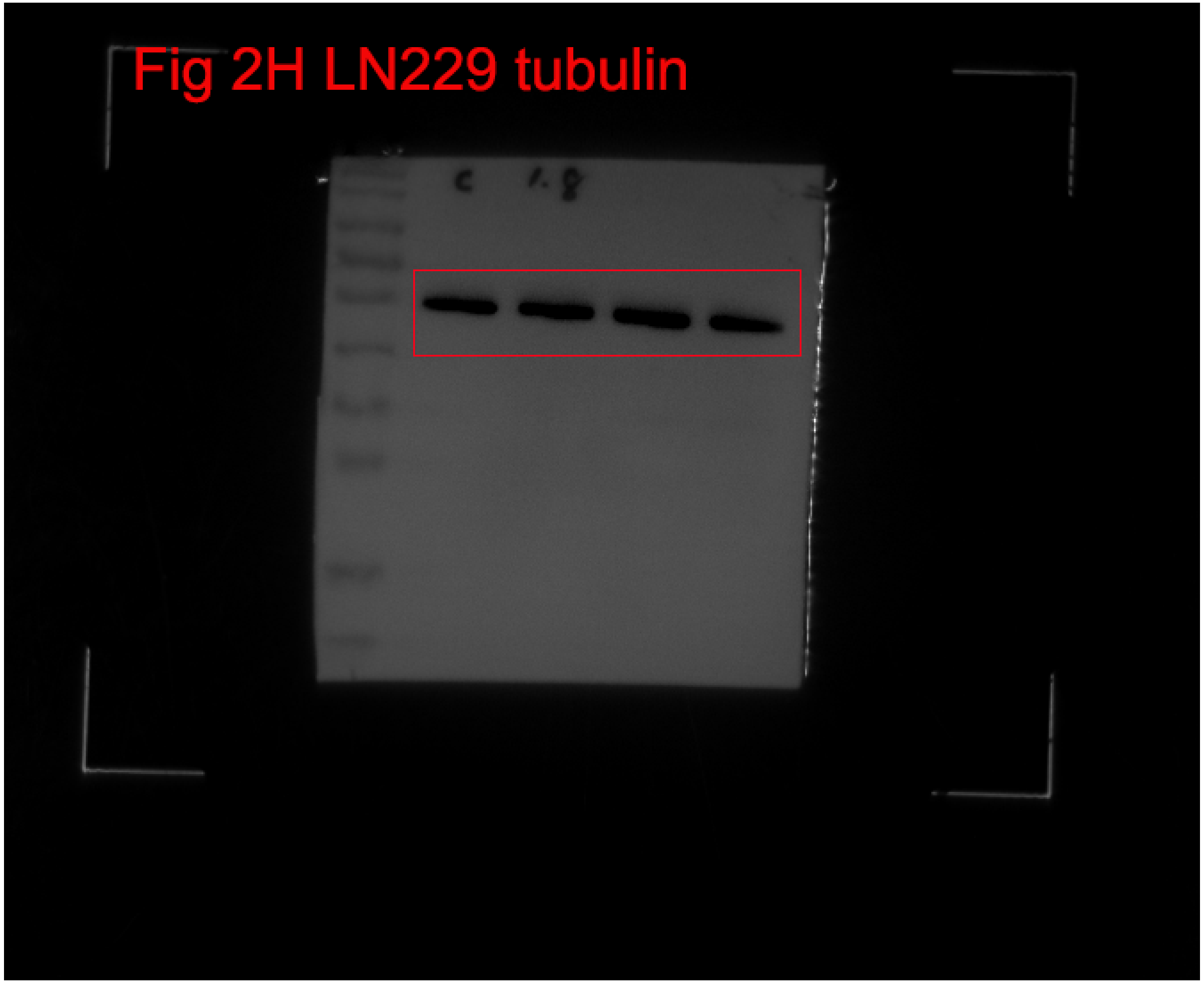

Fig 2H LN229 PARP and Cleaved PARP

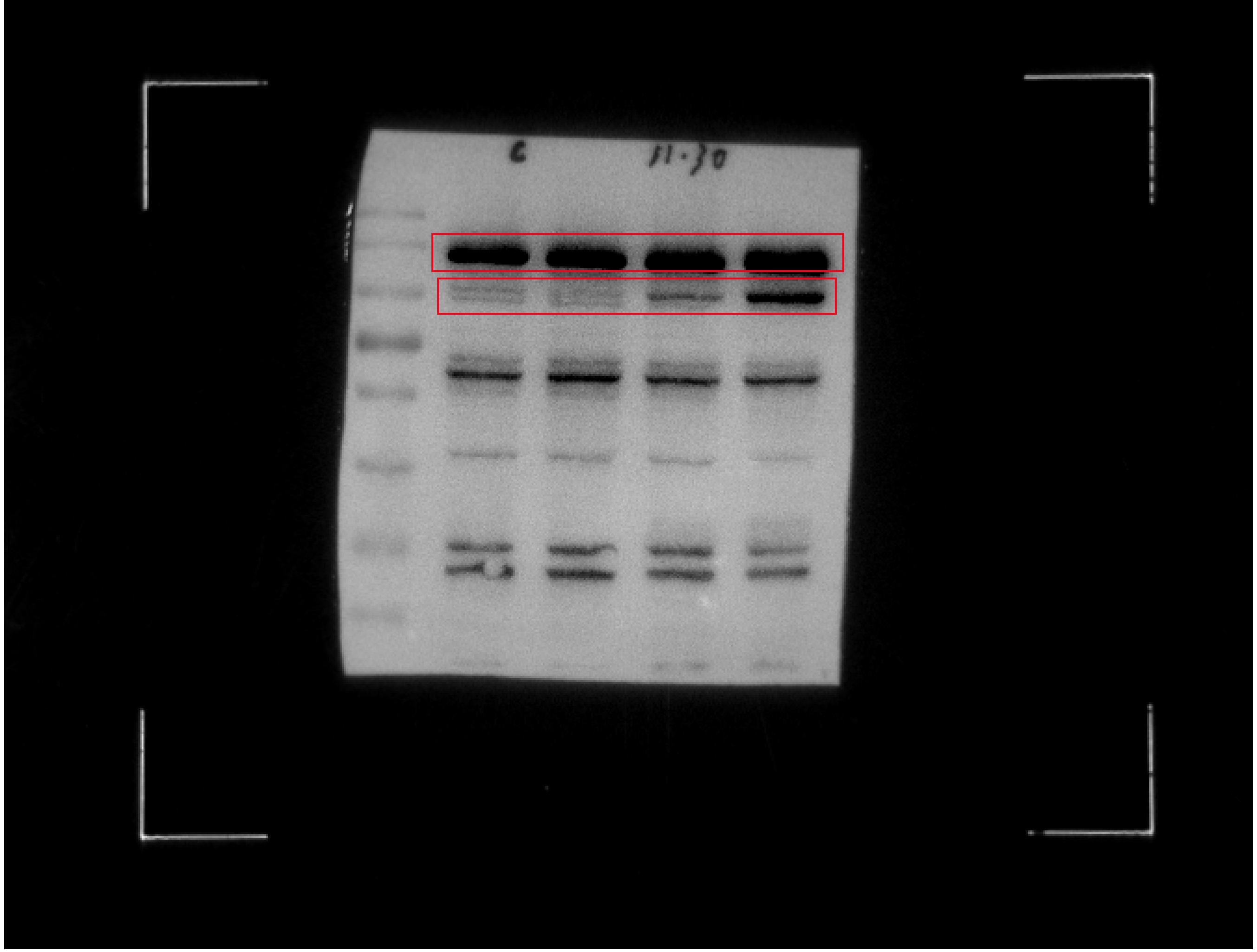

Fig 2H LN229 CC3 and Cleaved CC3

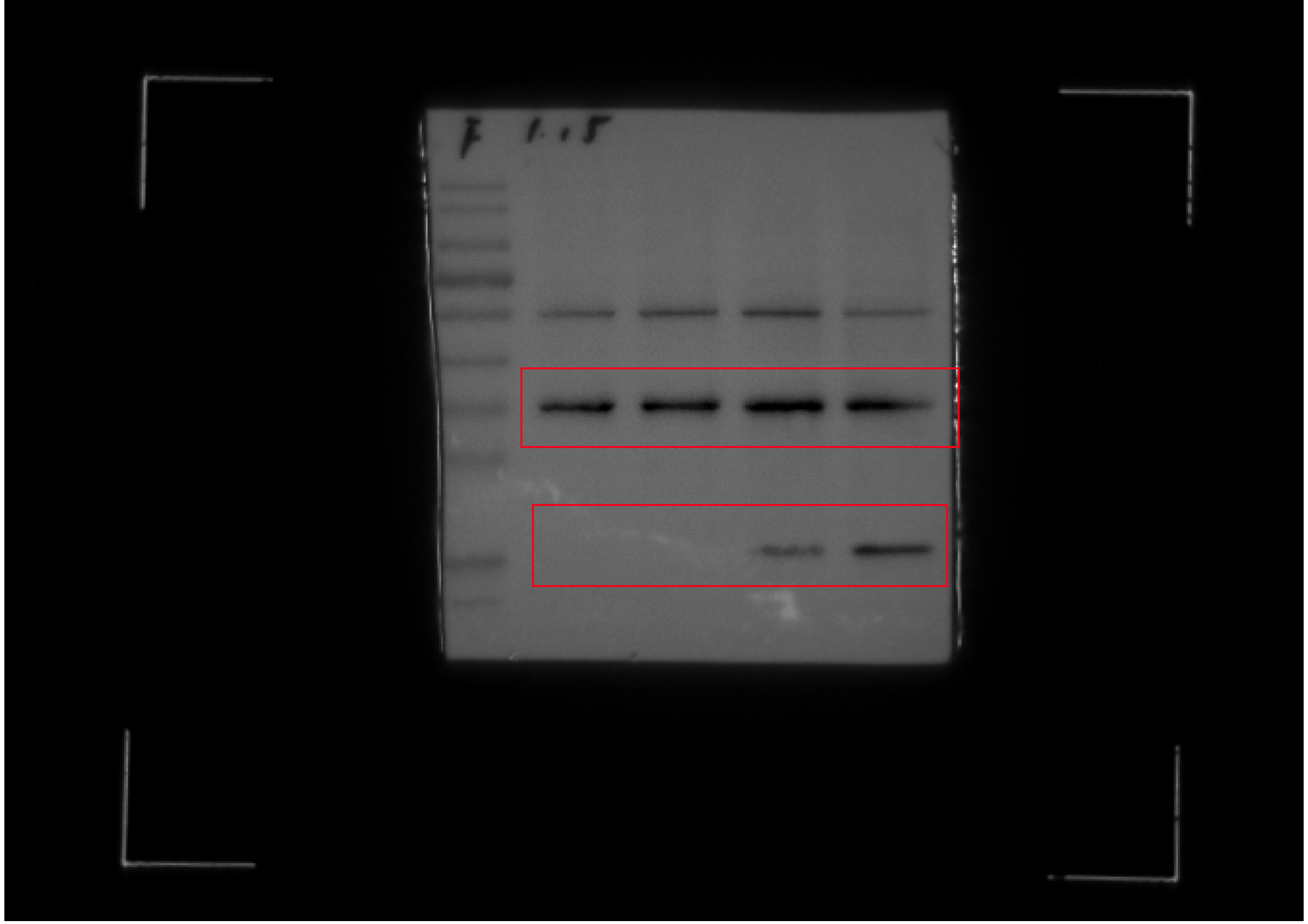

Fig 2H SHG141 PARP and Cleaved PARP

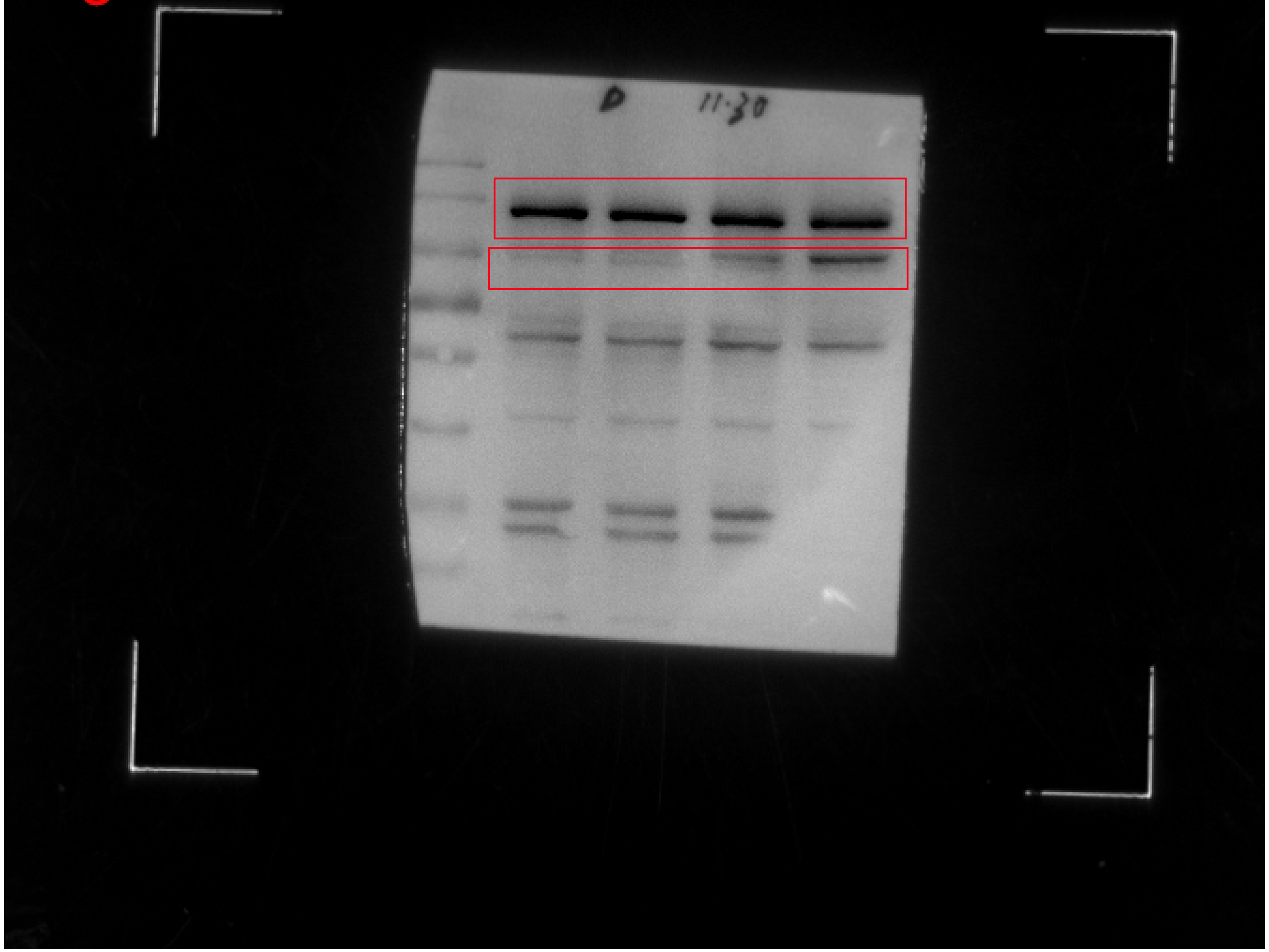

Fig 2H SHG141 tubulin

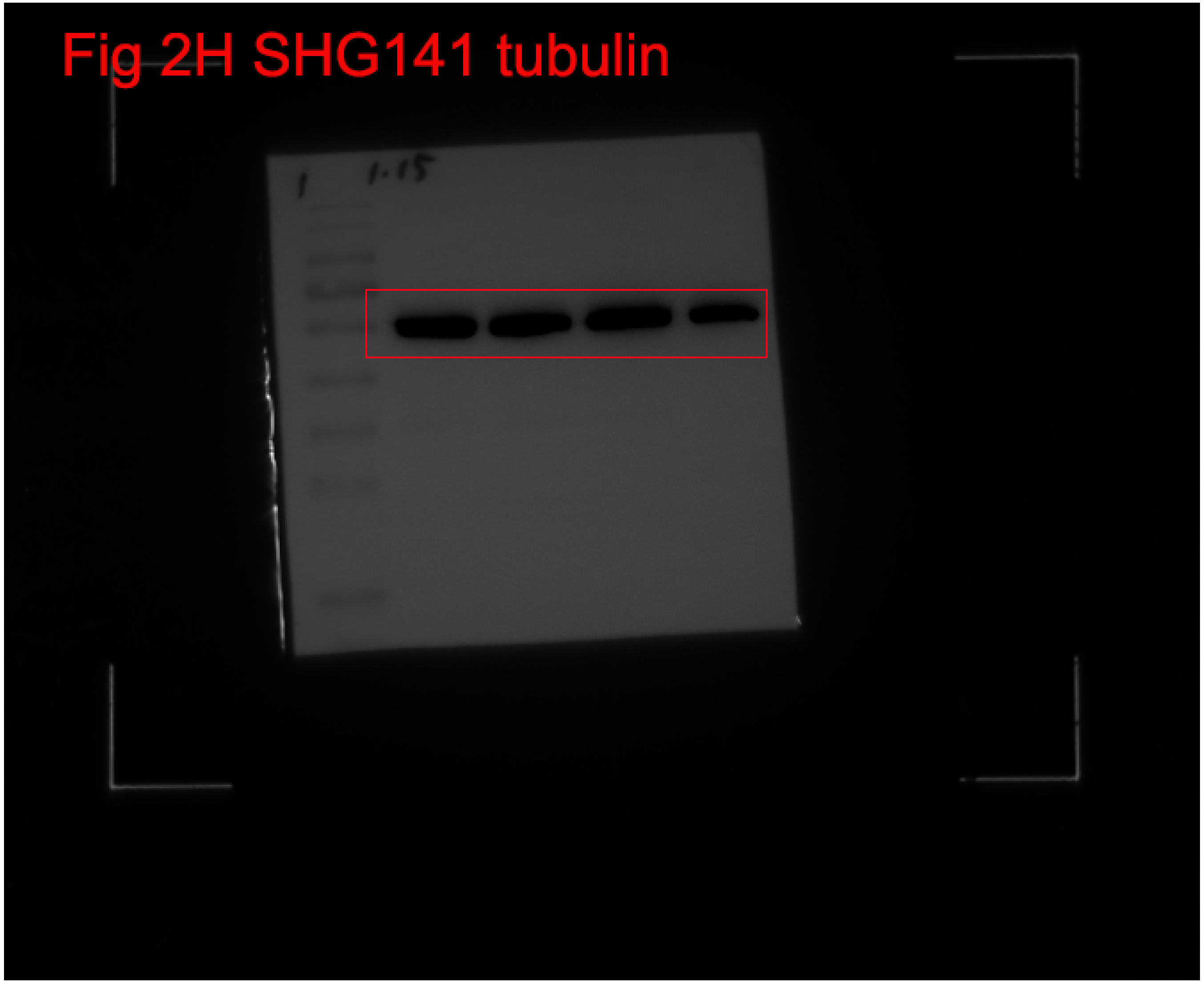

Fig 2H SHG141 CC3 and Cleaved CC3

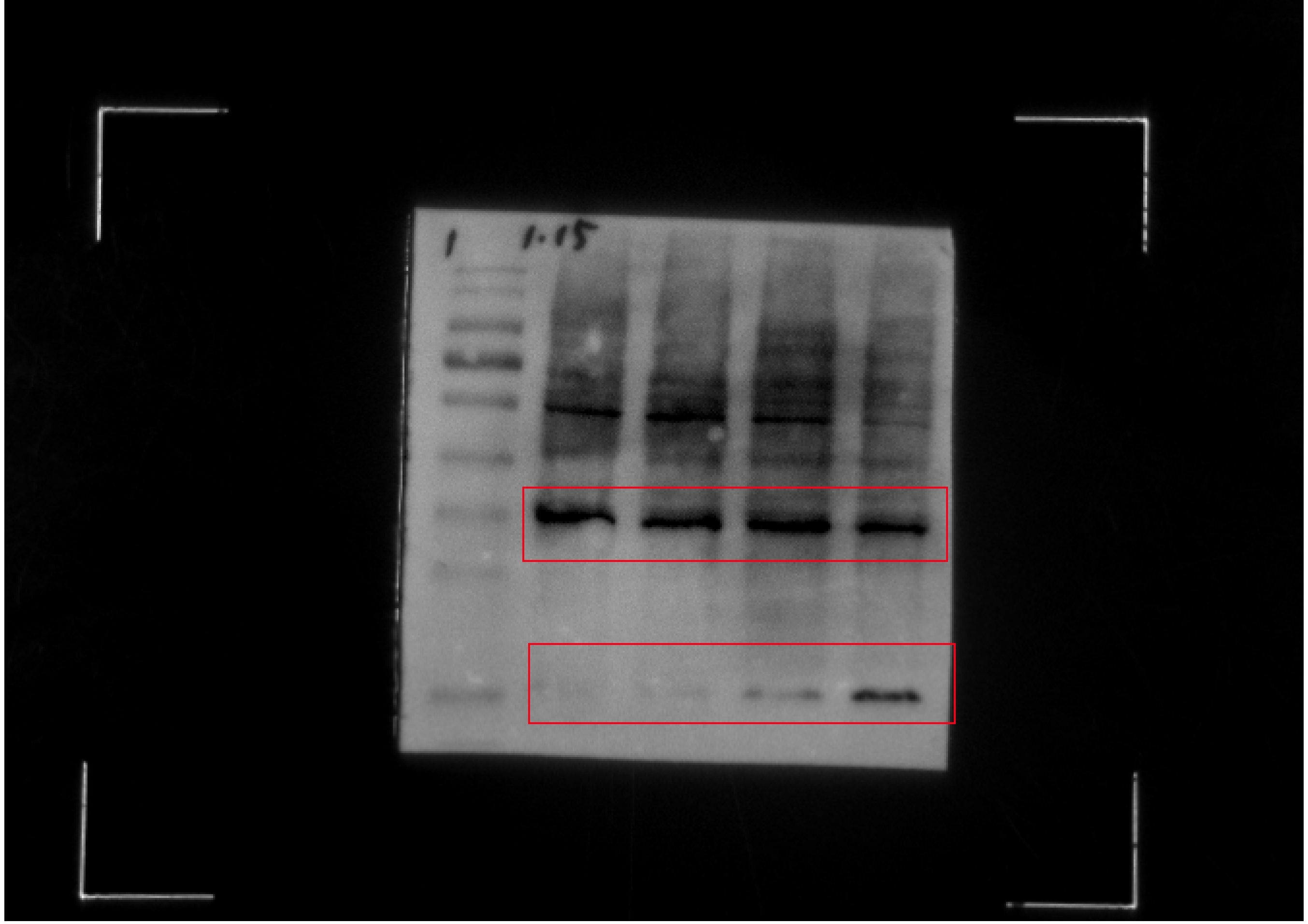

Fig 2H U251 PARP and Cleaved PARP

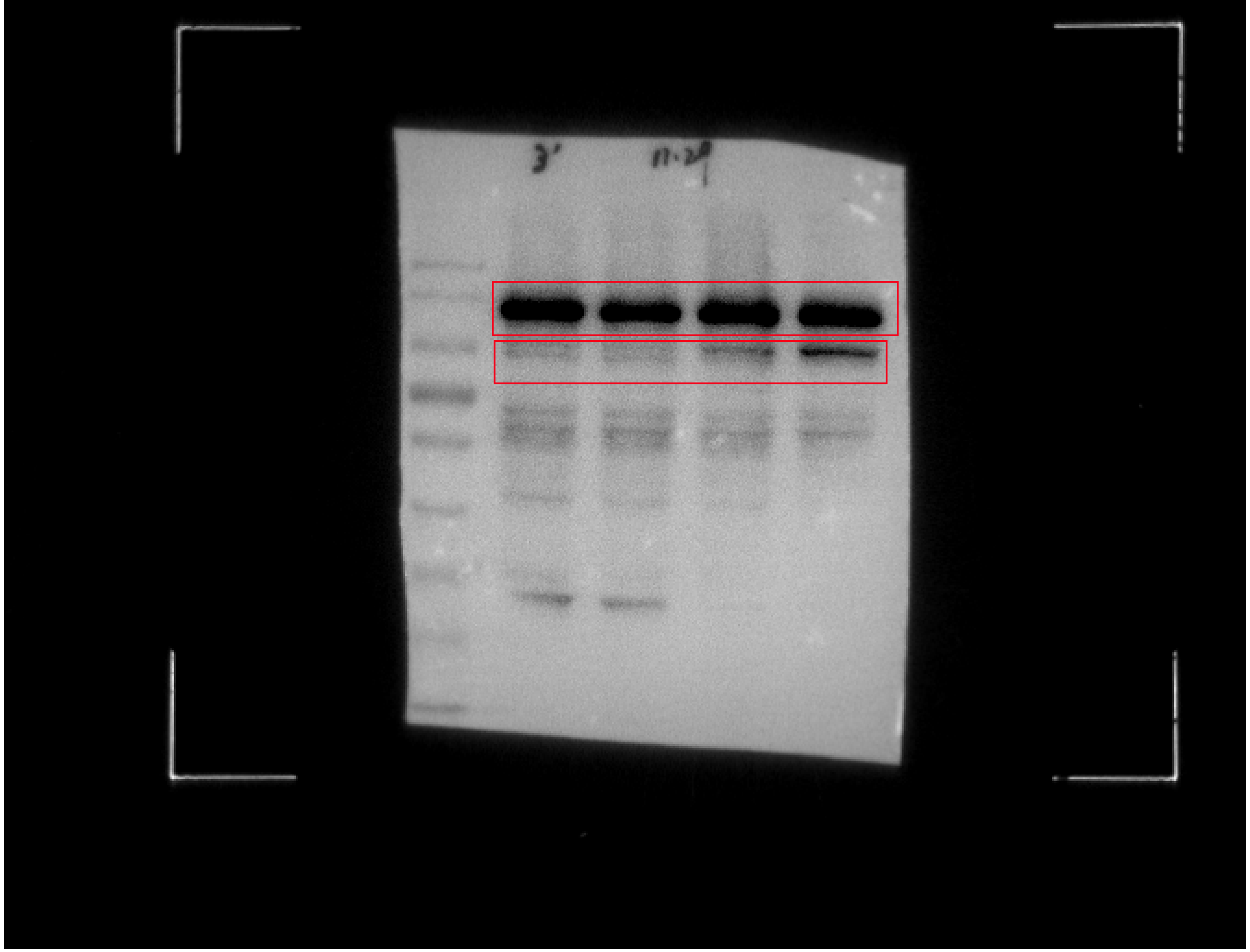

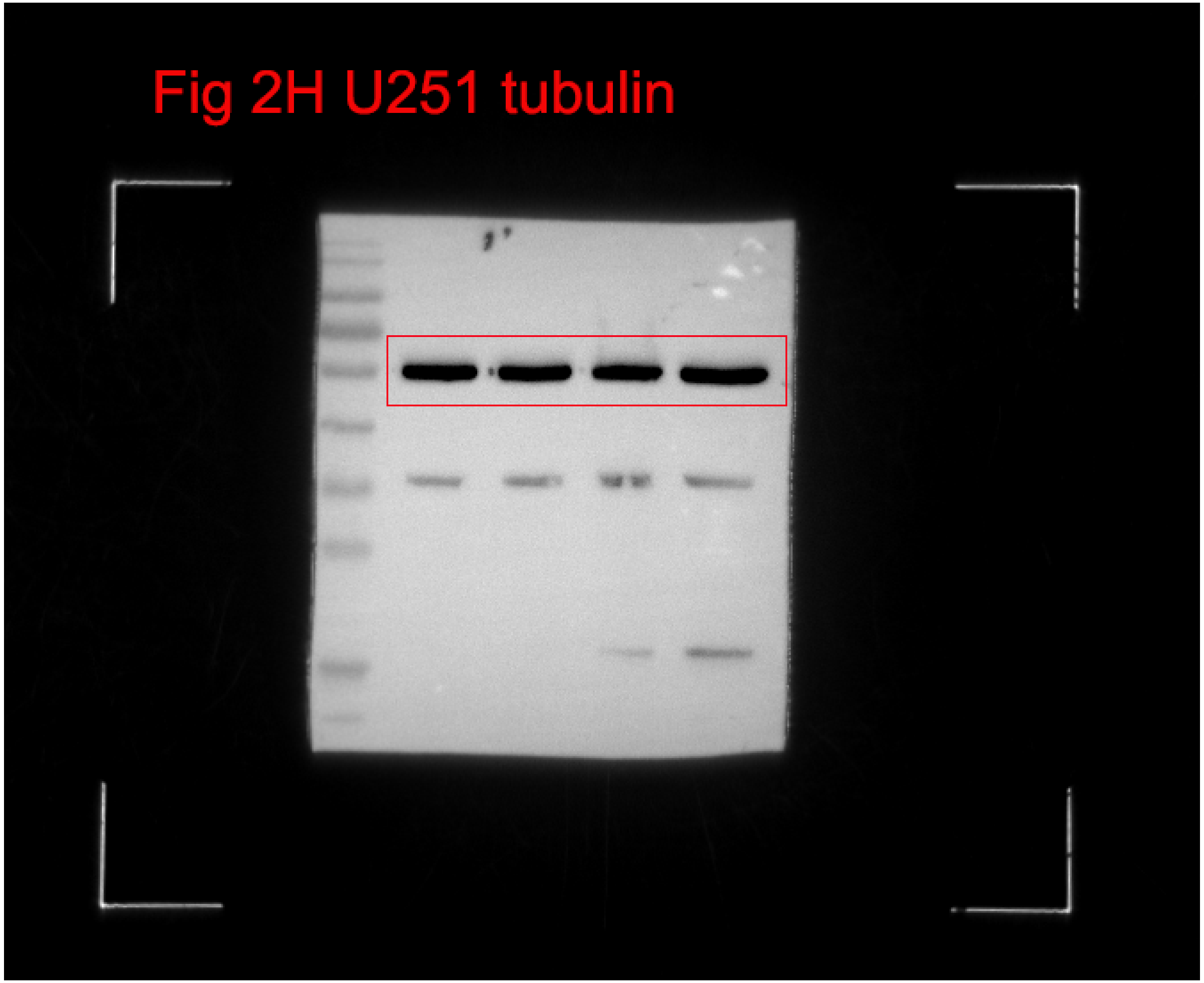
Fig 2H U251 tubulin

Fig 2H U251 CC3 and Cleaved CC3

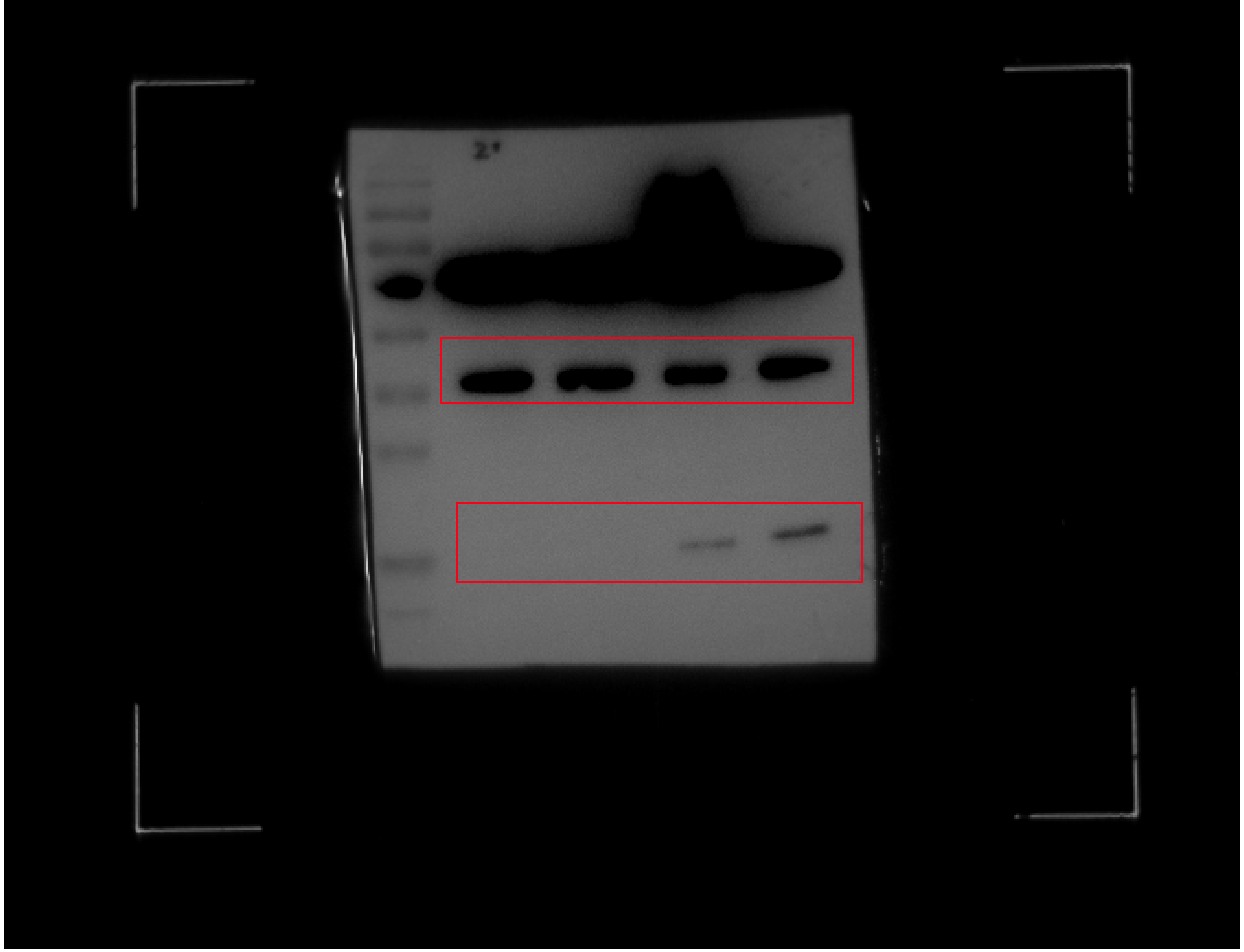

Fig 2H LN229 Cleaved CC3

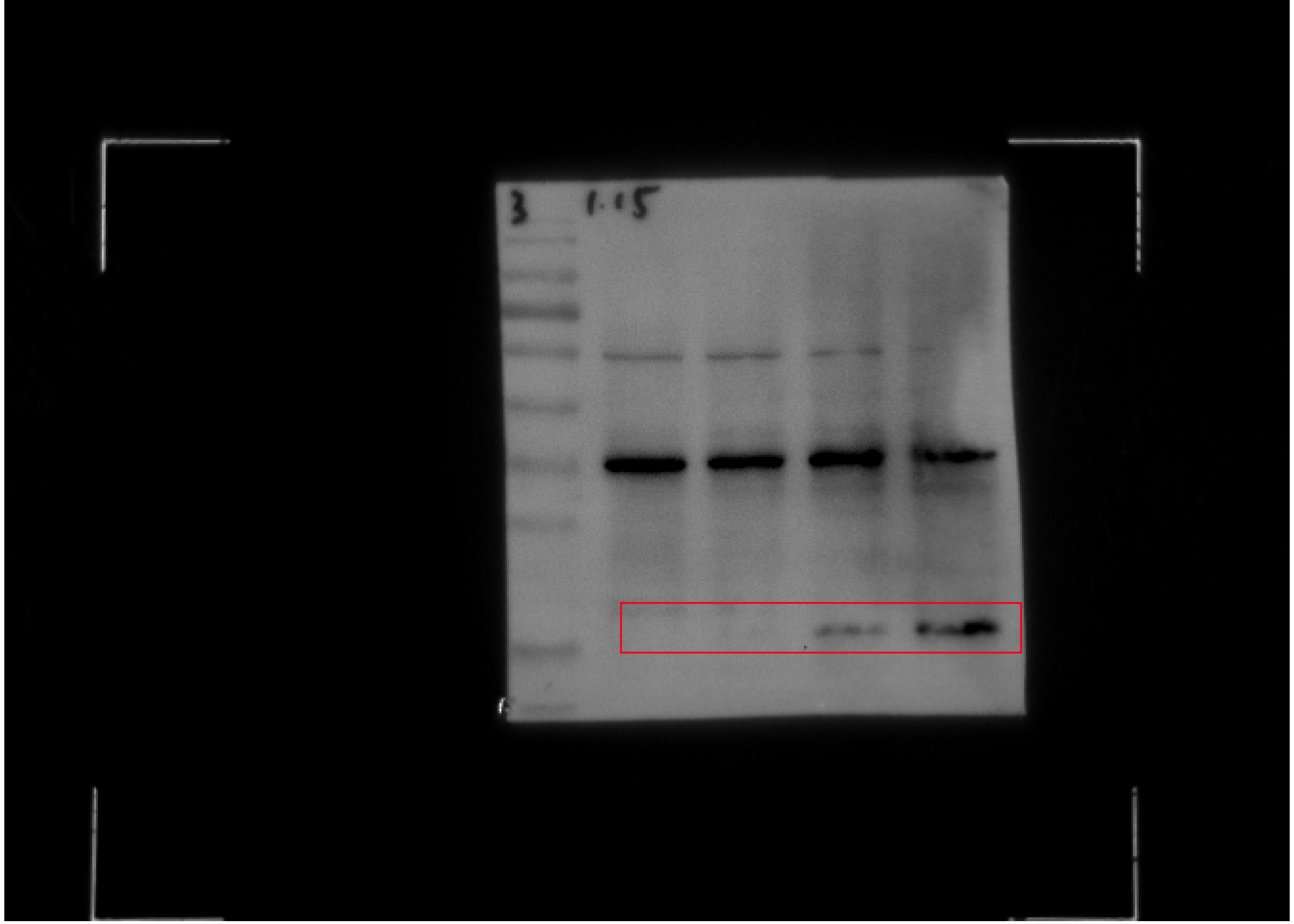

Fig 2M LN229 tubulin

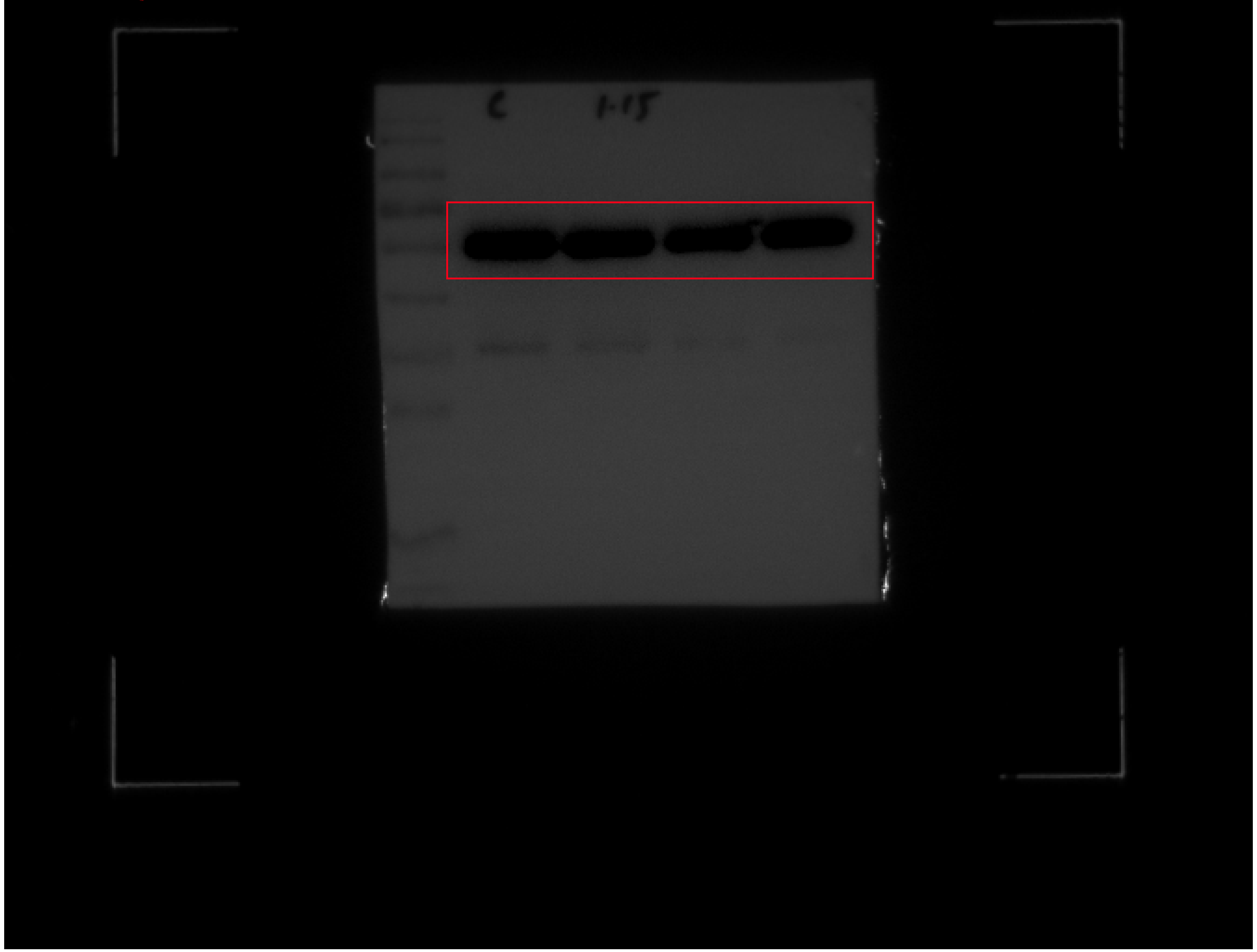

Fig 2M LN229 CC3

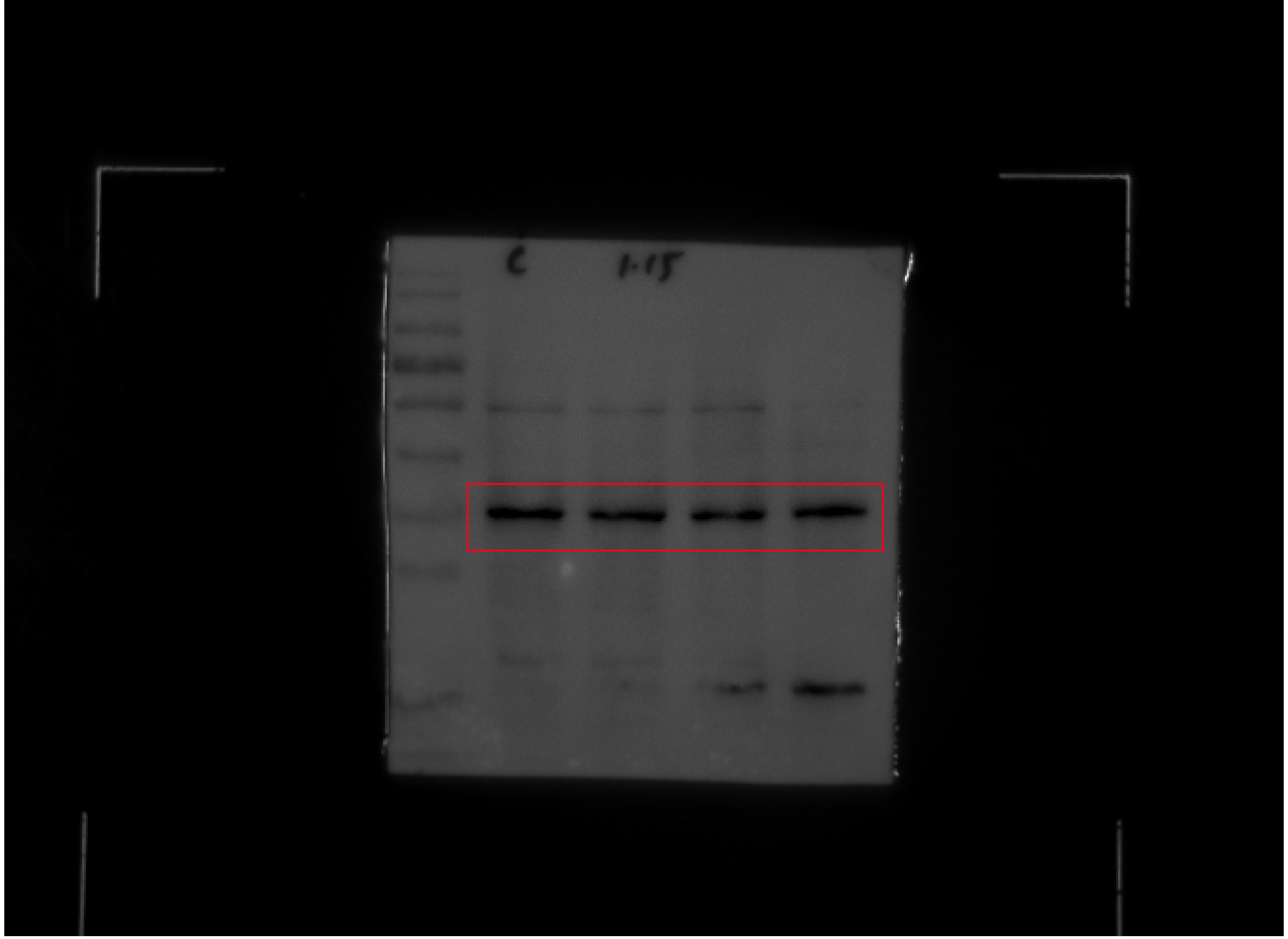

Fig 2M SHG141 Cleaved CC3

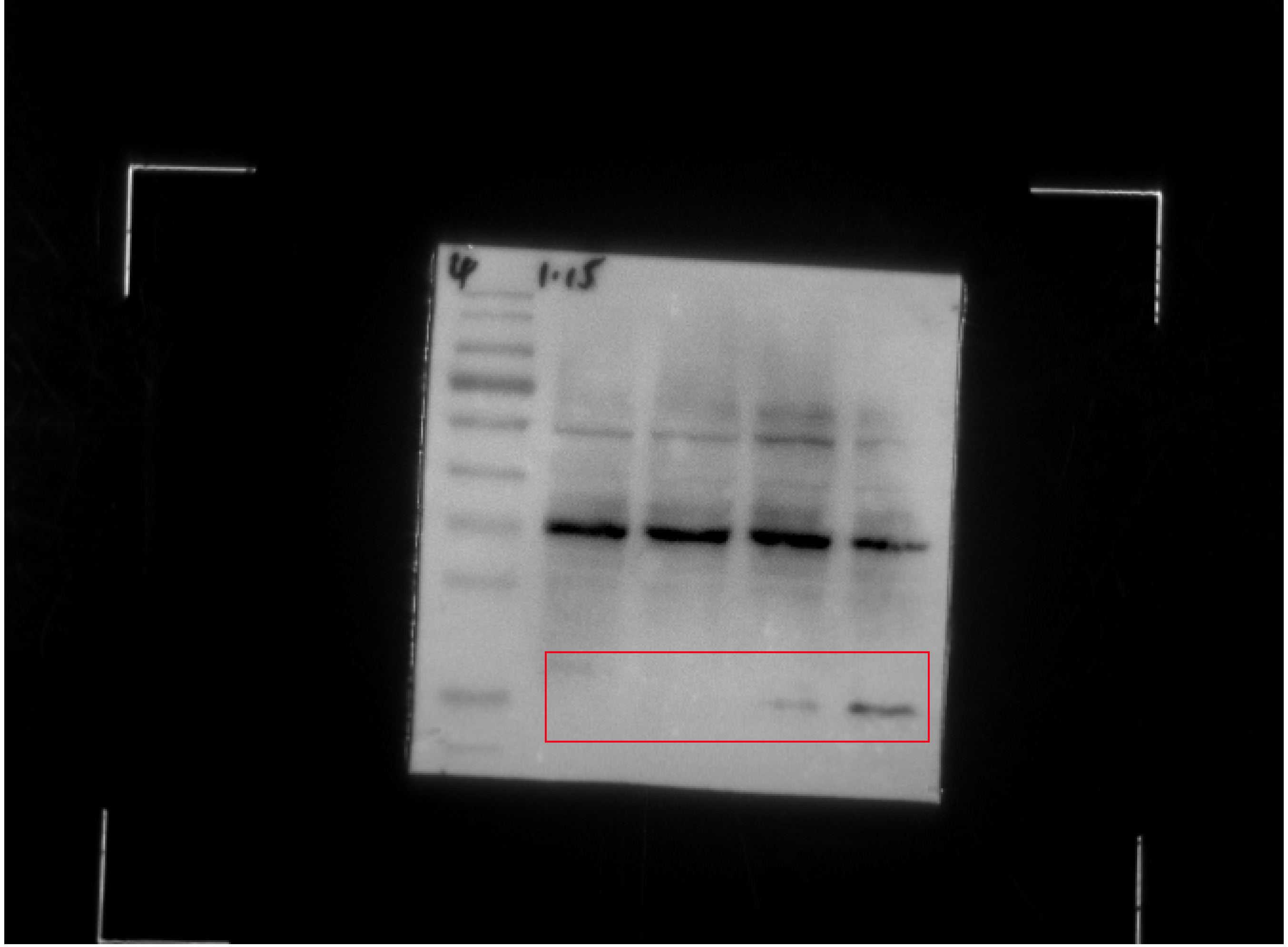

Fig 2M SHG141 tubulin

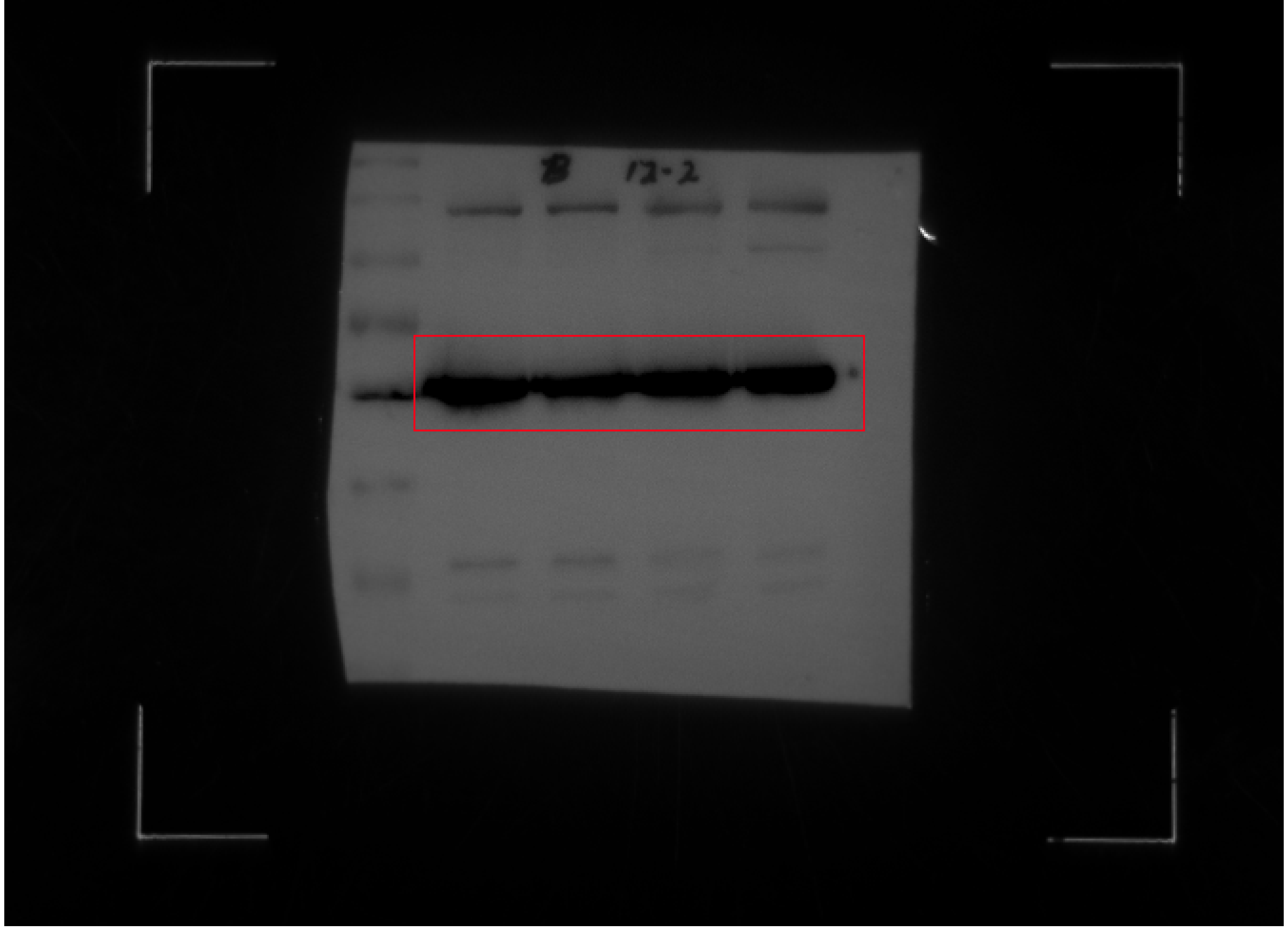

Fig 2M SHG141 CC3

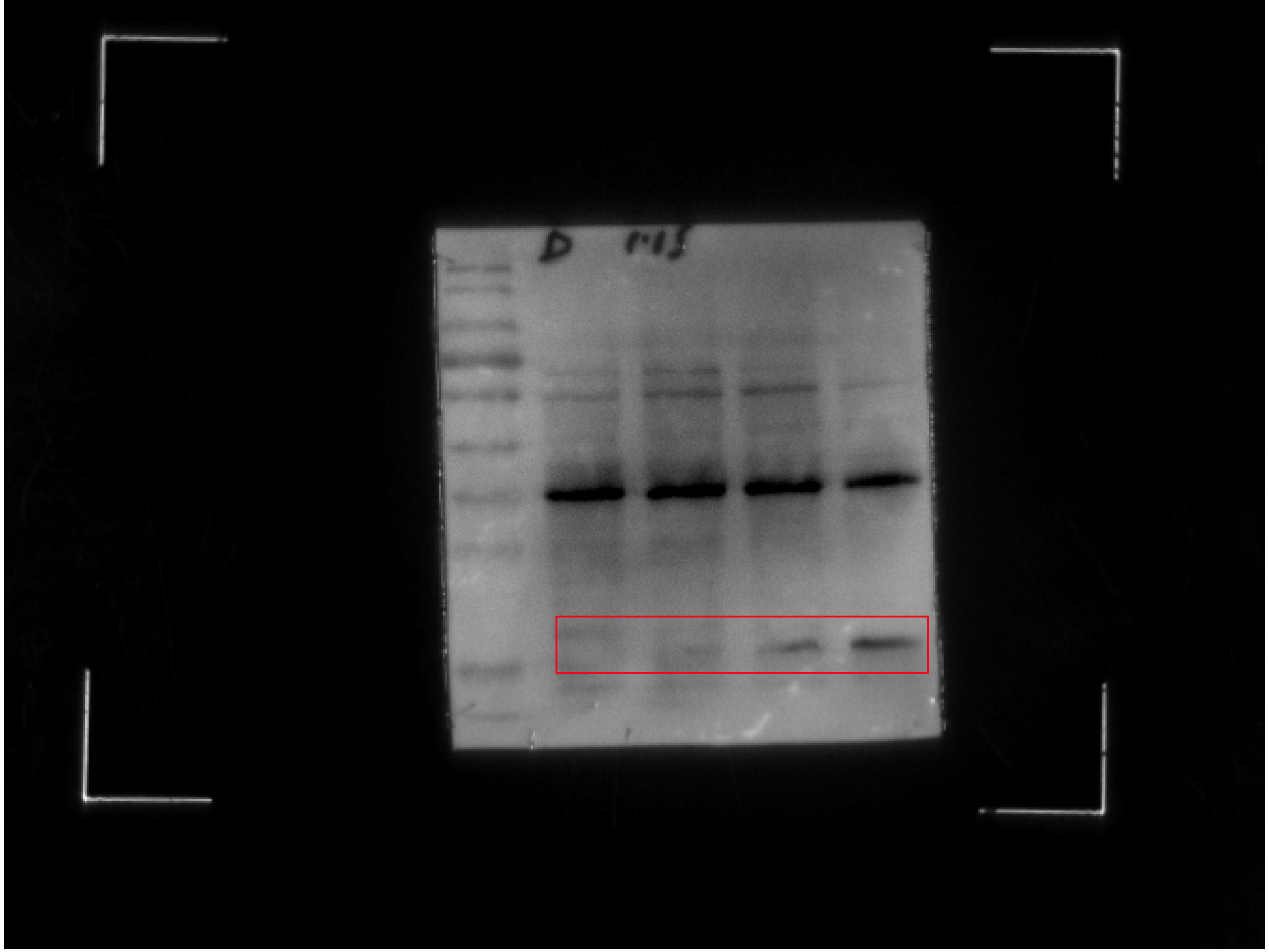

Fig 2M U251 Cleaved CC3

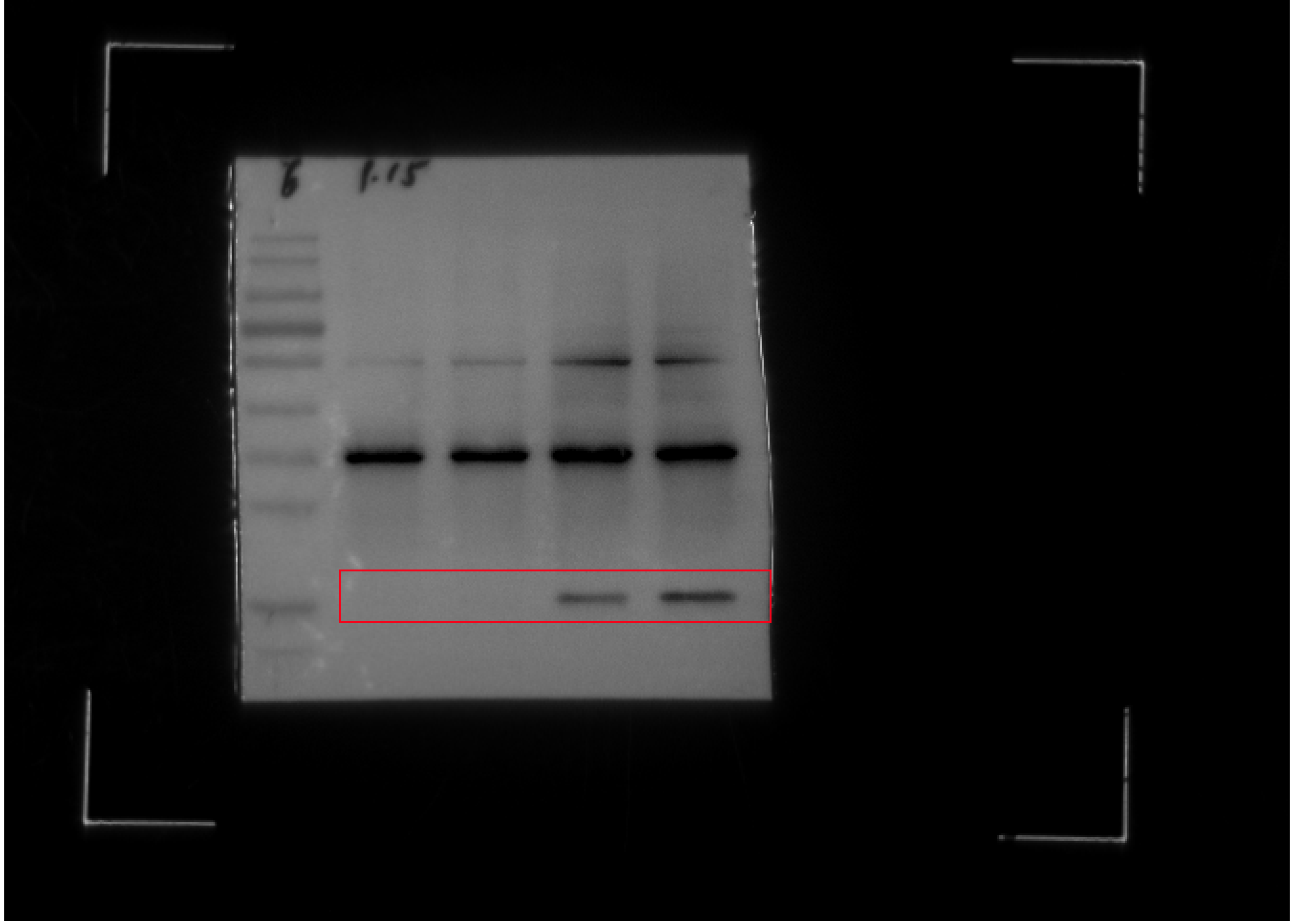

Fig 2M U251 tubulin

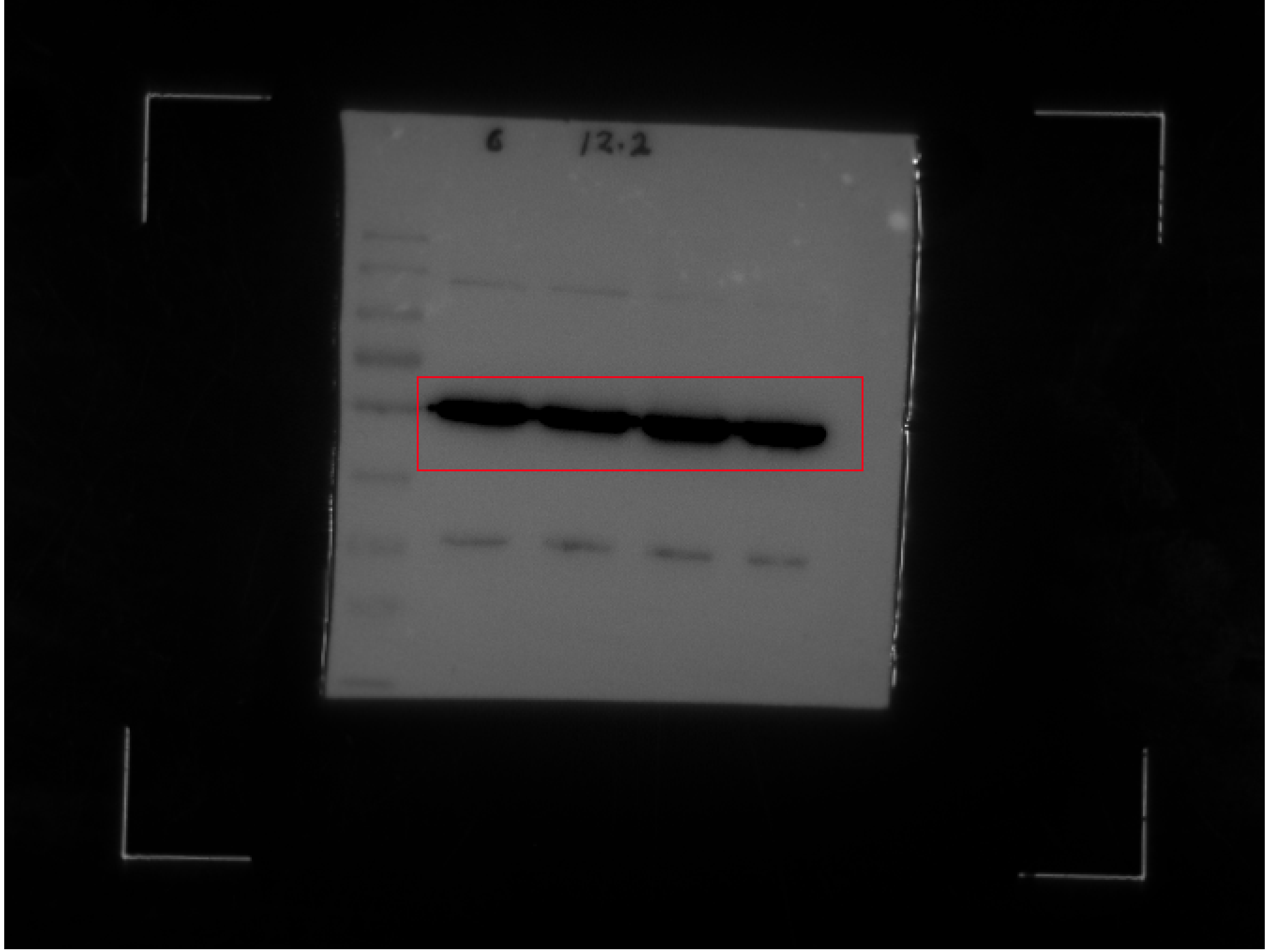

Fig 2M U251 CC3

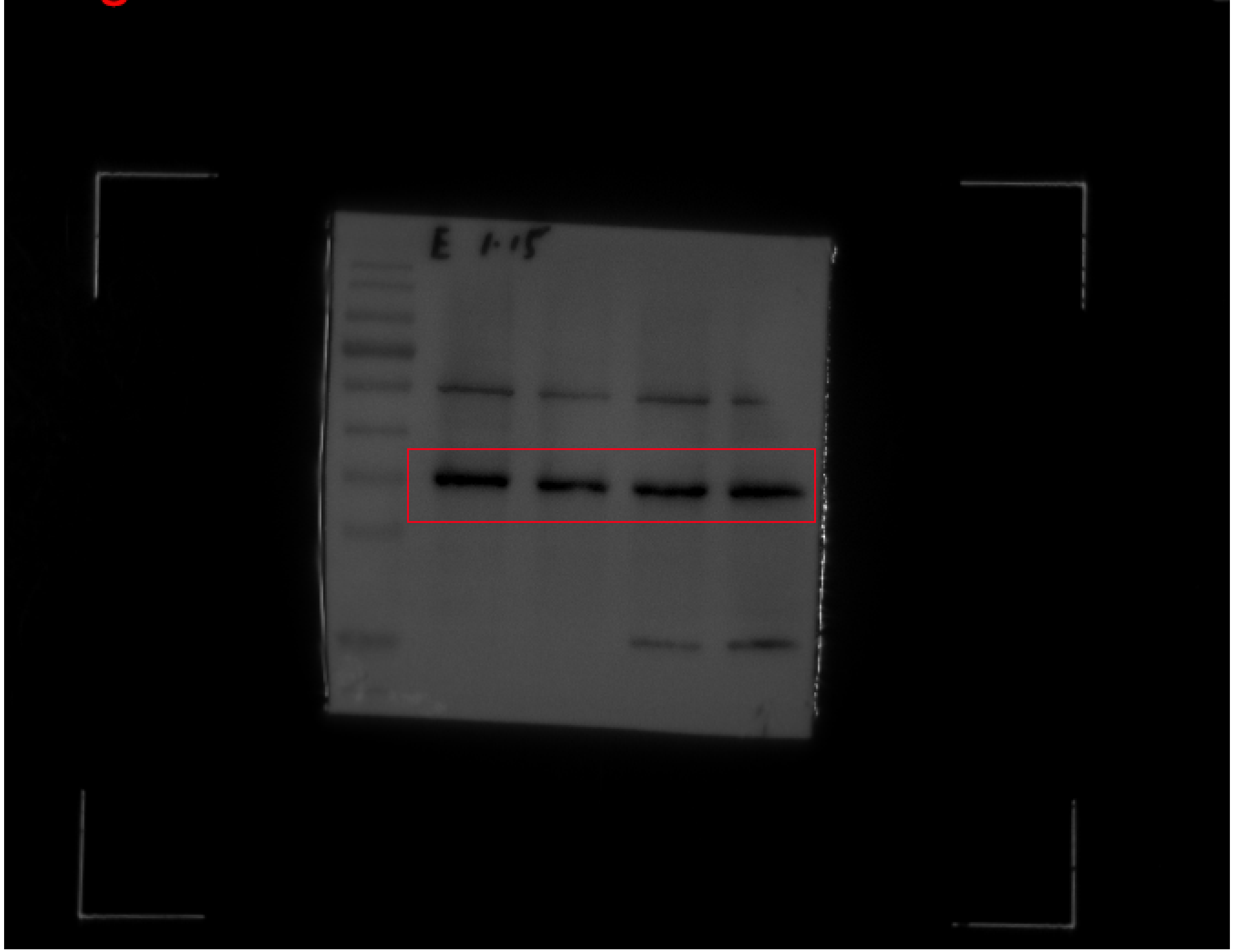

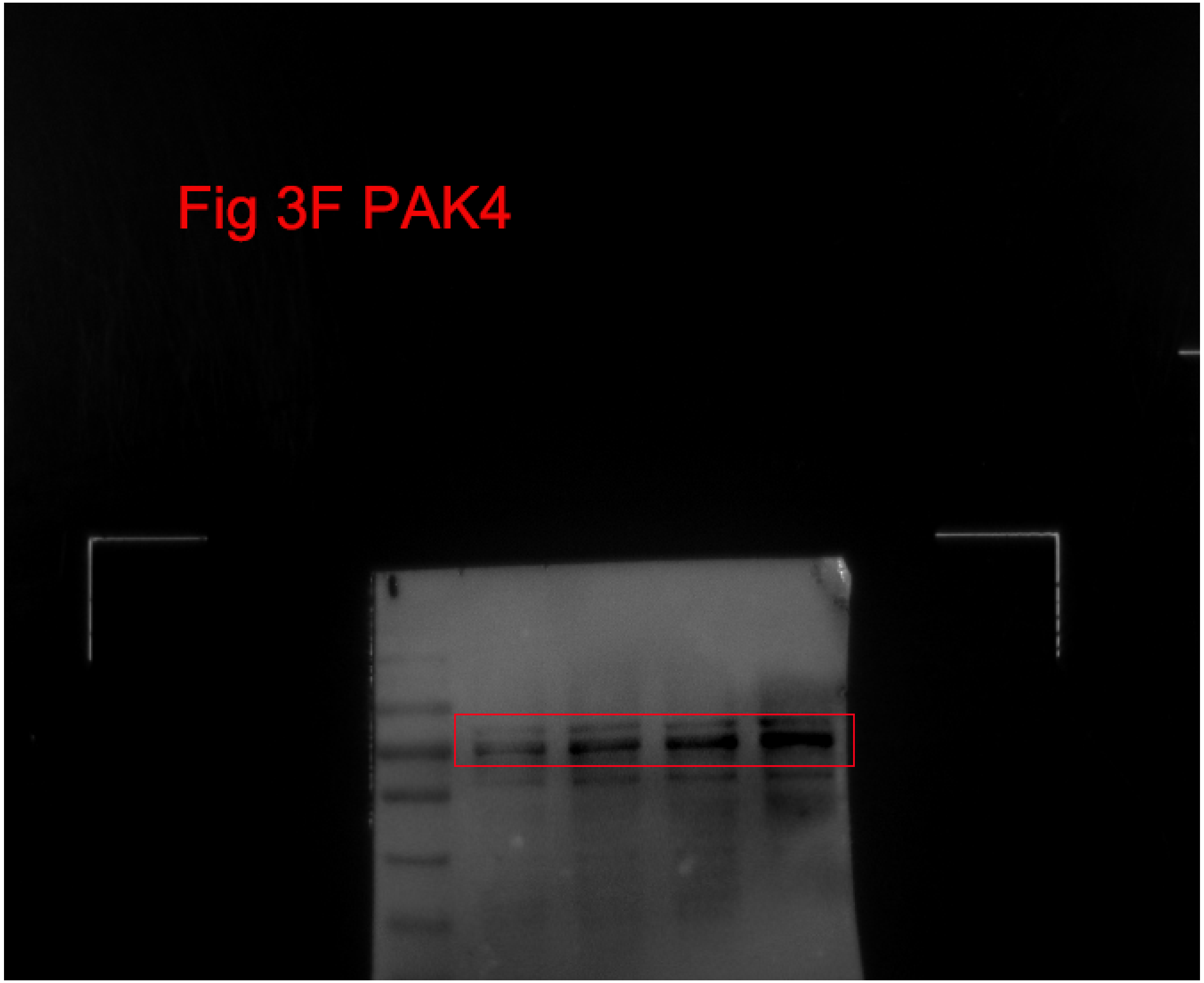
Fig 3F PAK4

Fig 3F tubulin

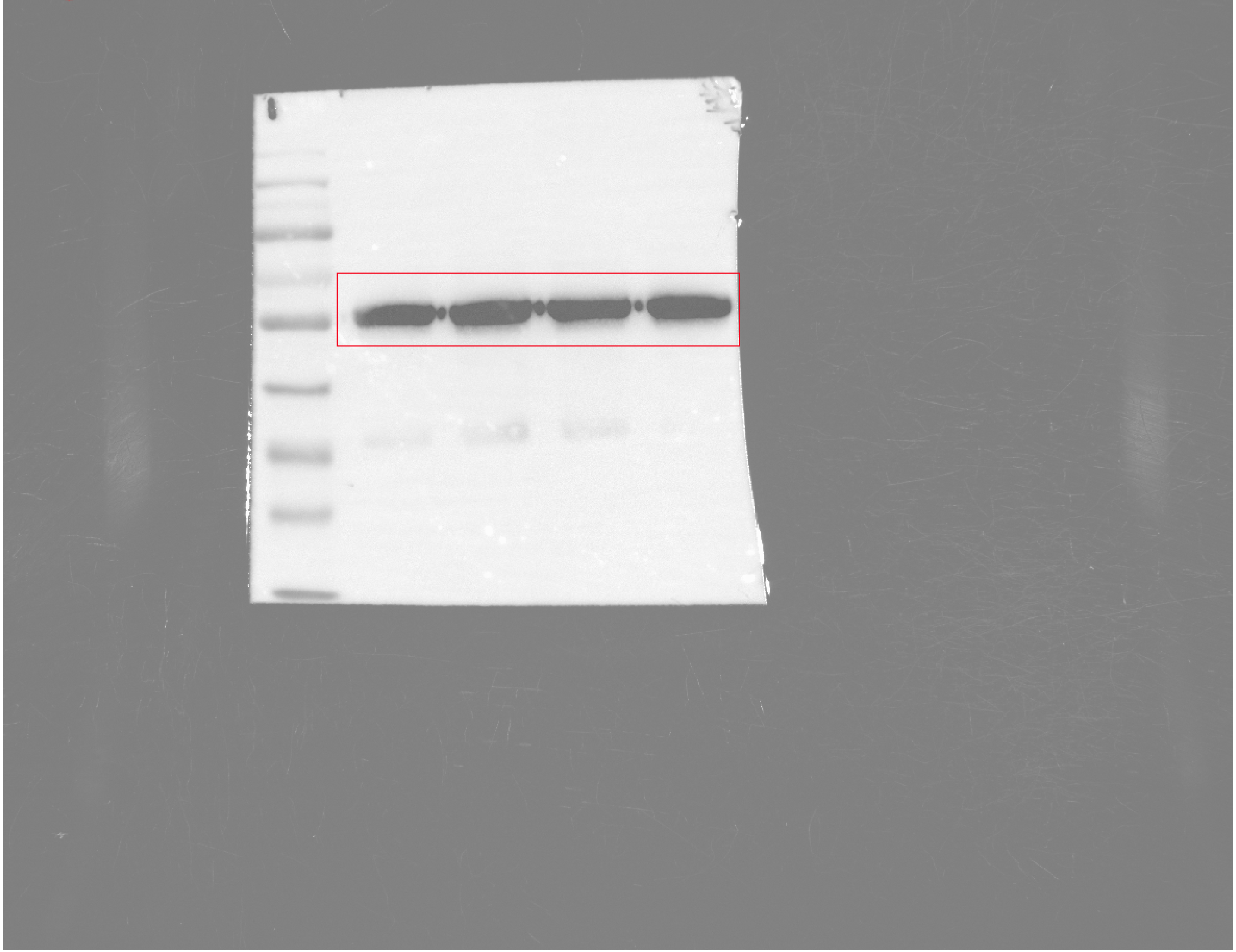

Fig 4B SHG141

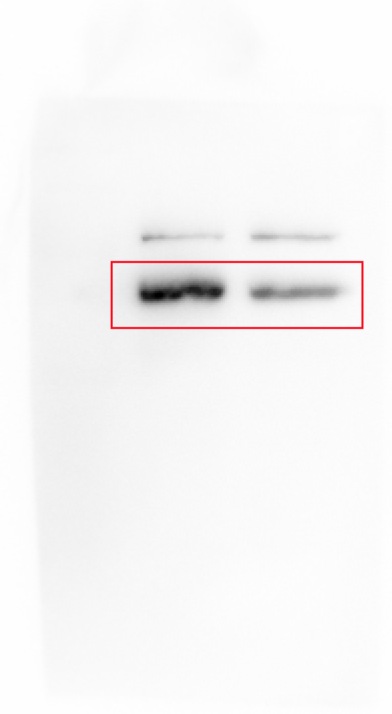

Fig 4B U251 PAK4

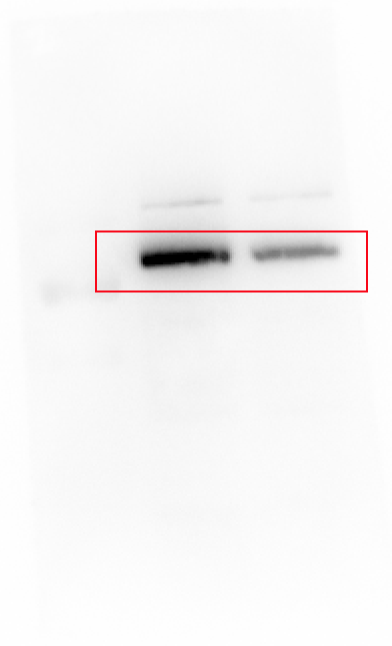

Fig 4B LN229

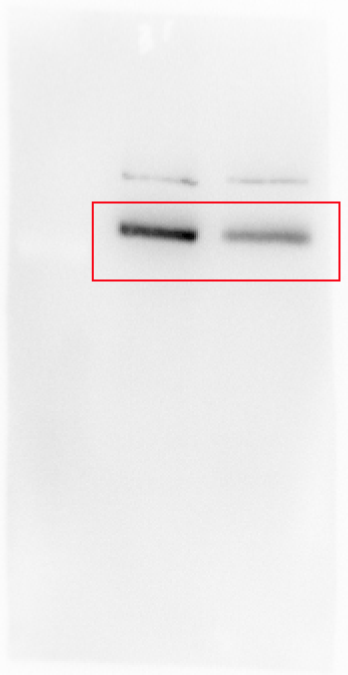

Fig 4B SHG141 p-STAT3

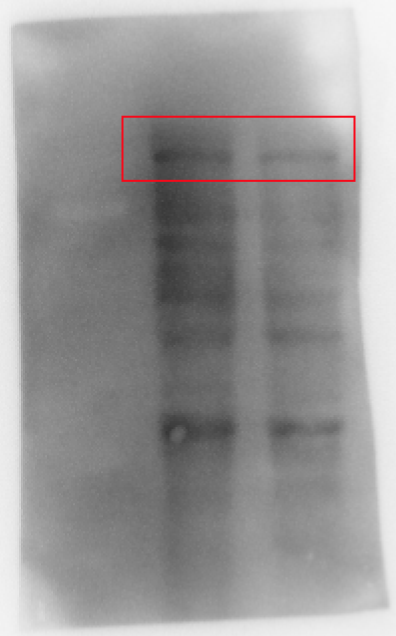

Fig 4B U251 p-STAT3

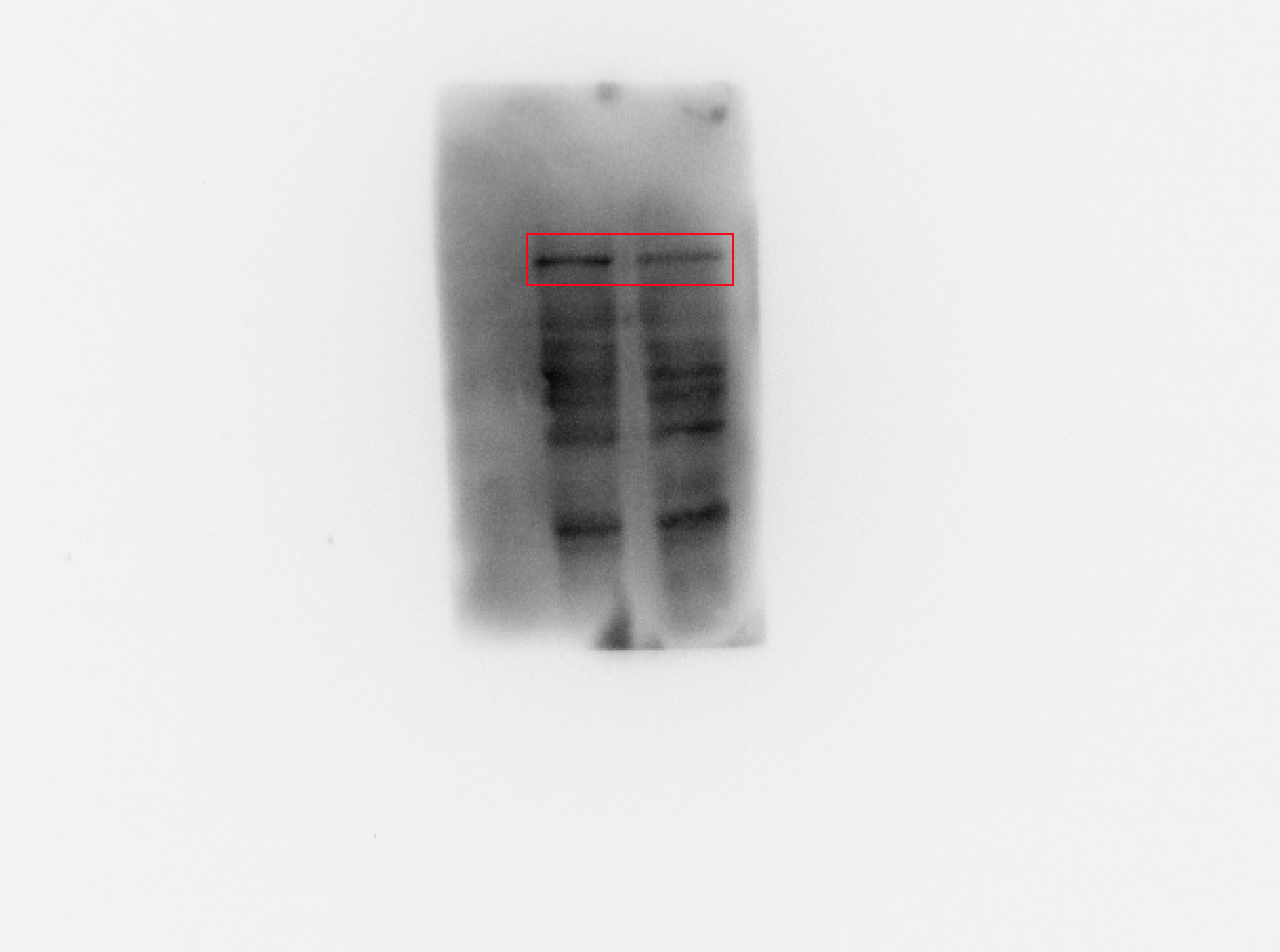

Fig 4B LN229 p-STAT3

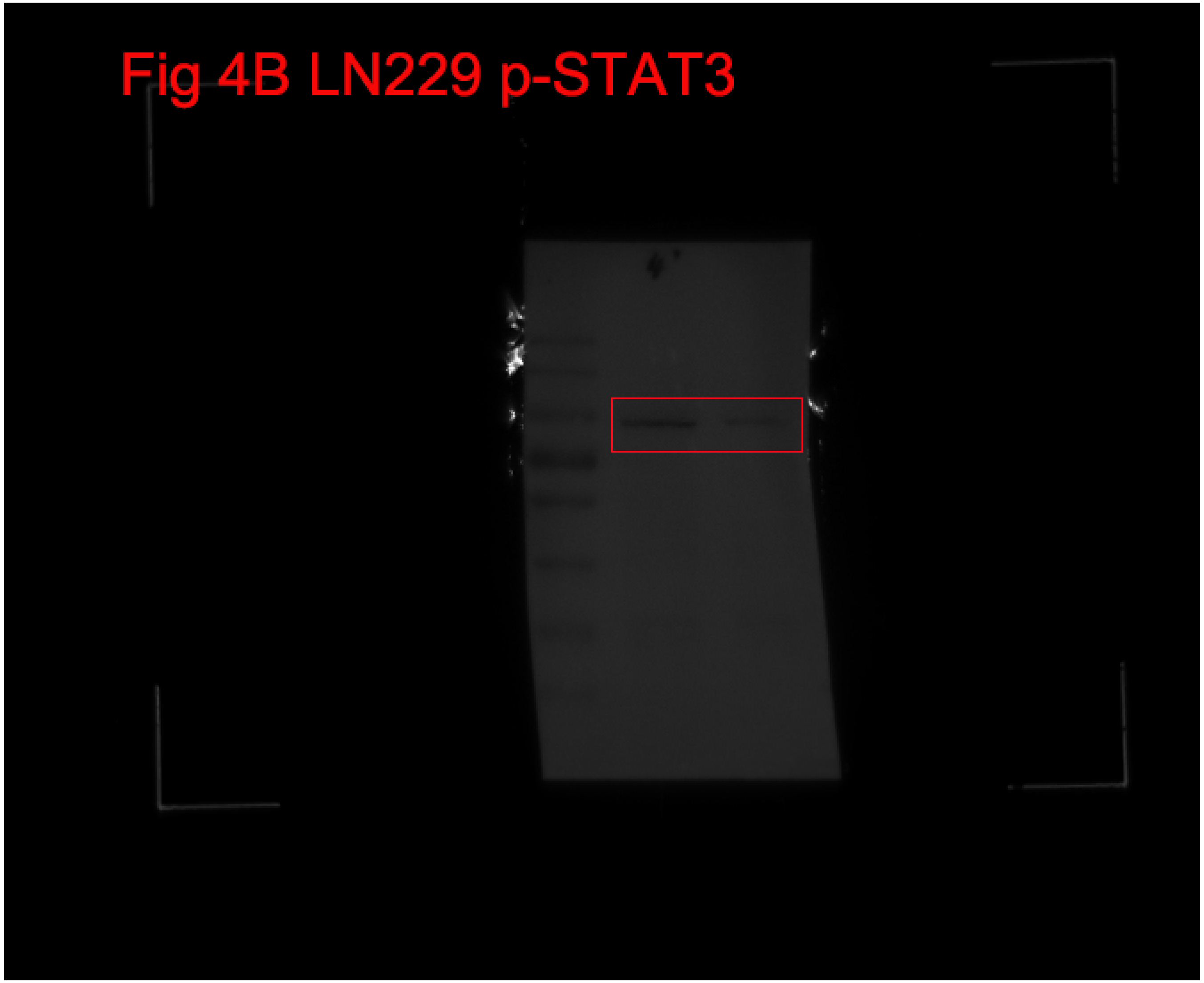

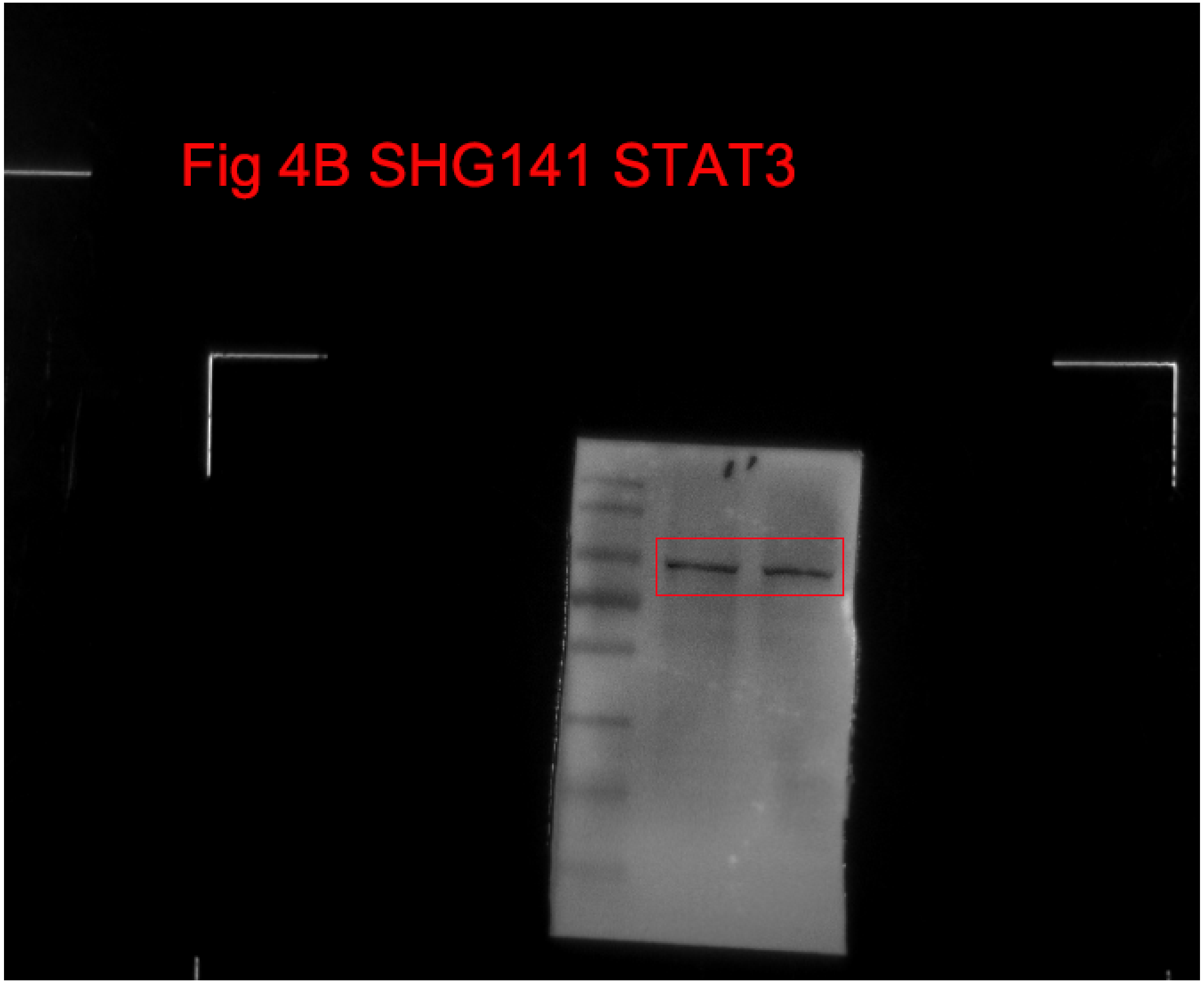

Fig 4B SHG141 STAT3

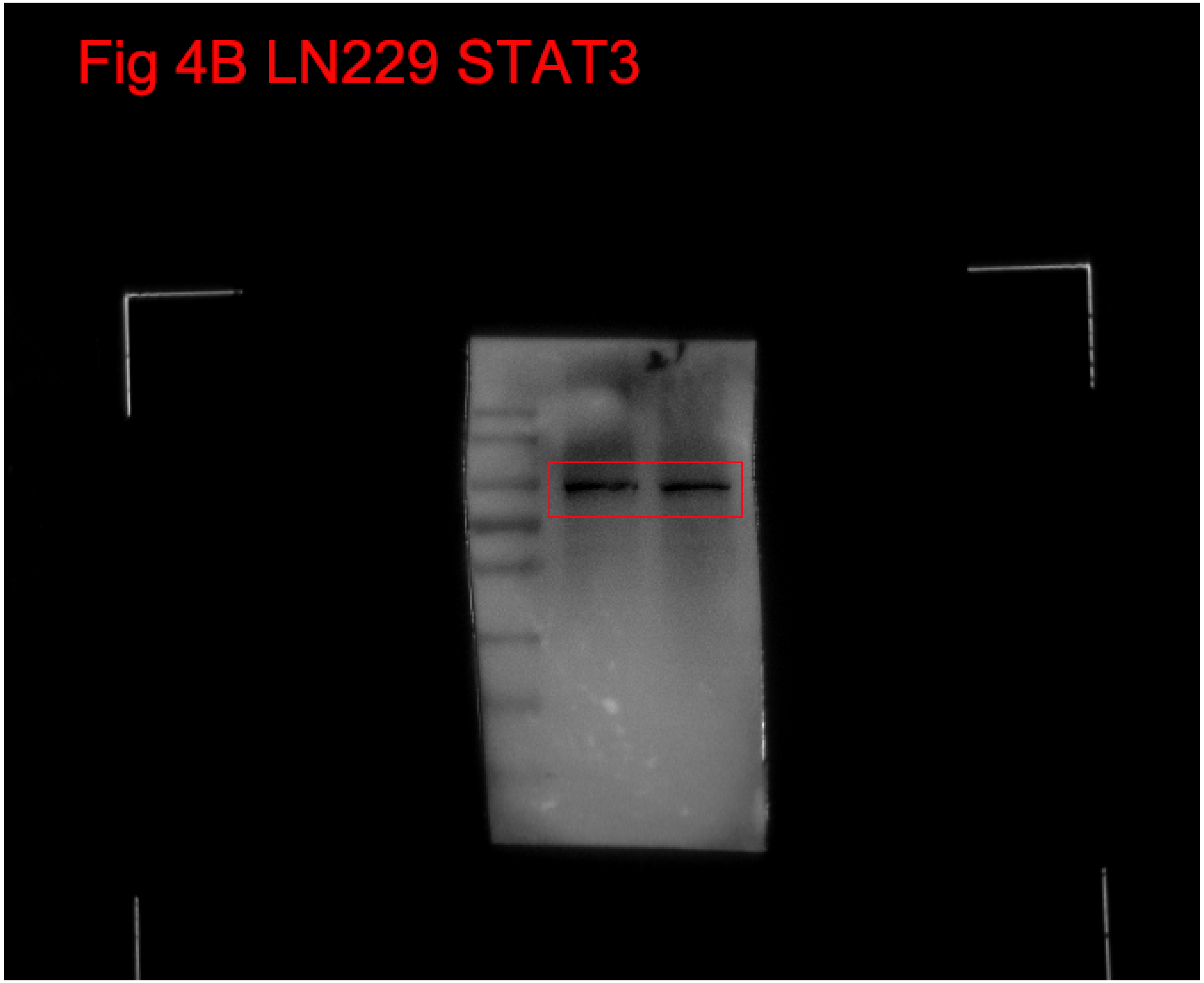
Fig 4B LN229 STAT3

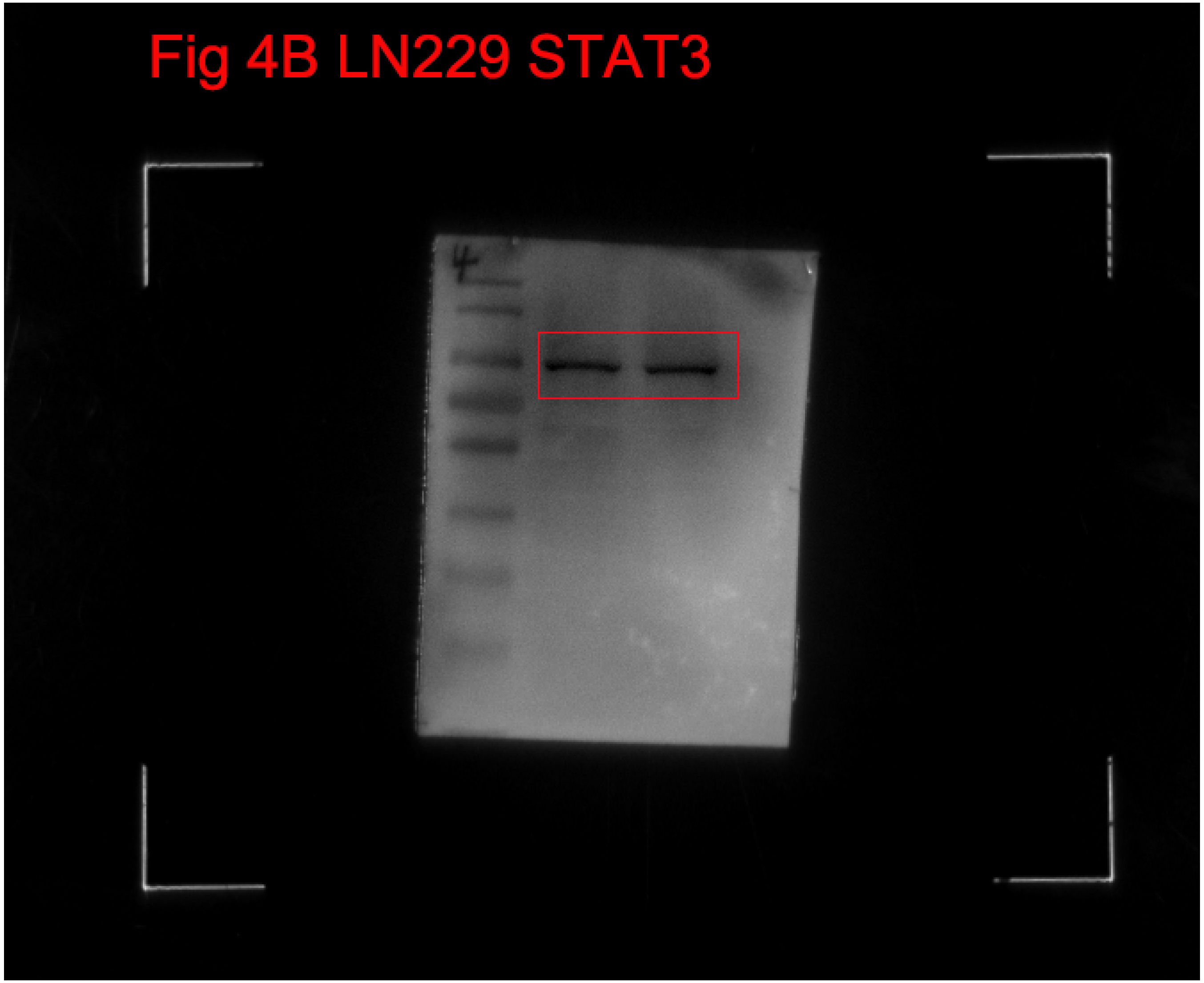
Fig 4B LN229 STAT3

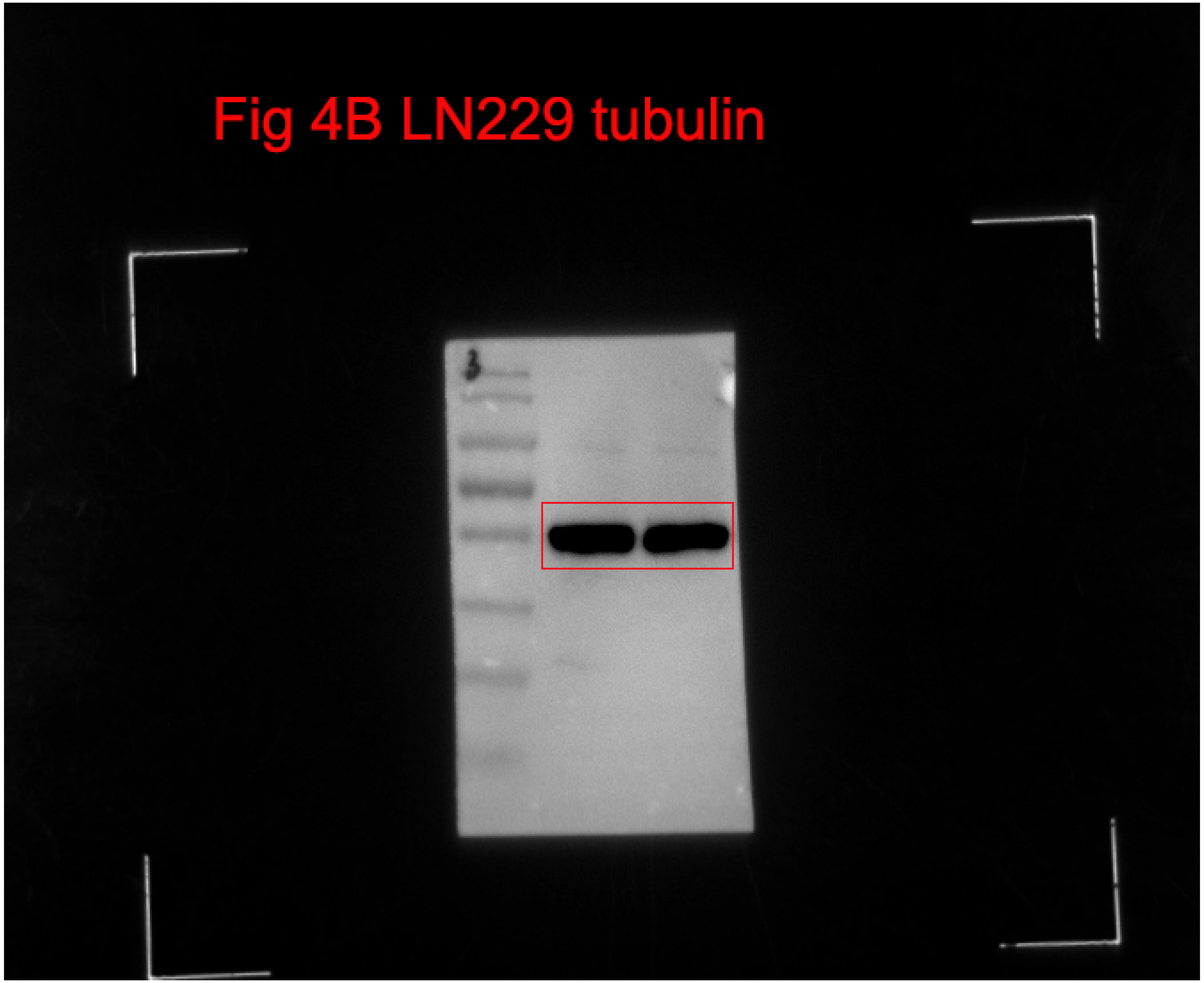
Fig 4B LN229 tubulin

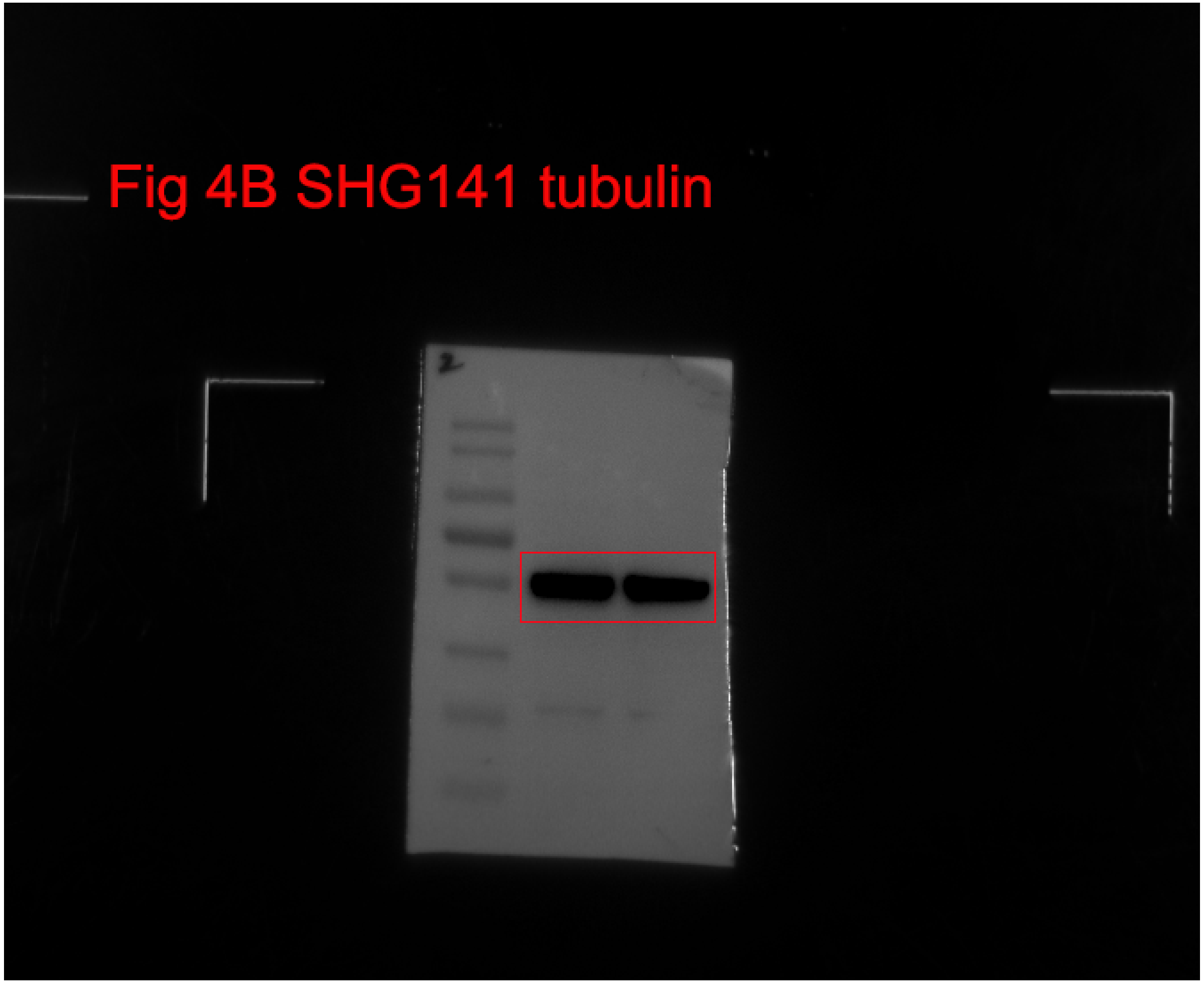
Fig 4B SHG141 tubulin

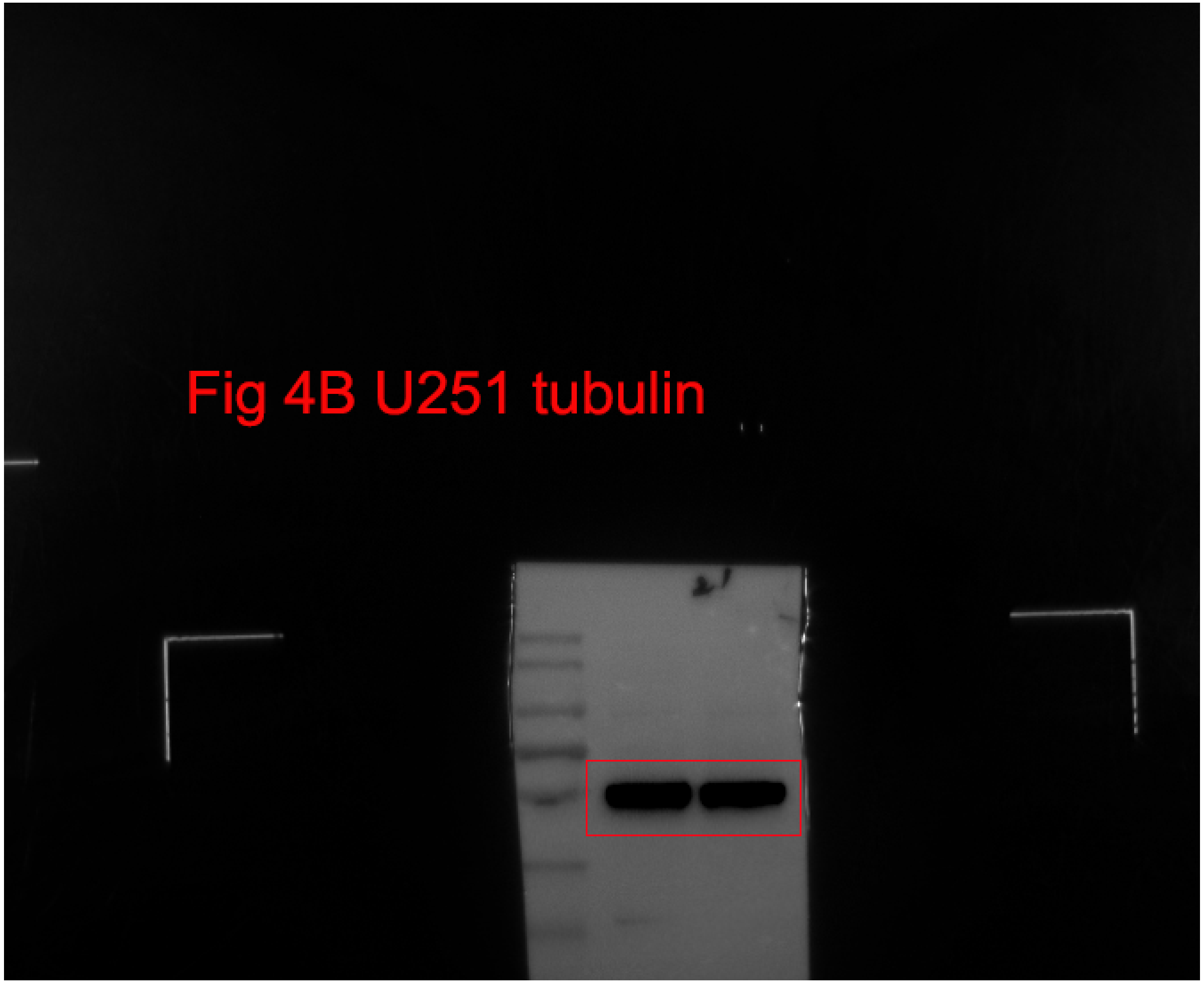
Fig 4B U251 tubulin

Fig 4B SHG141 VEGFR2

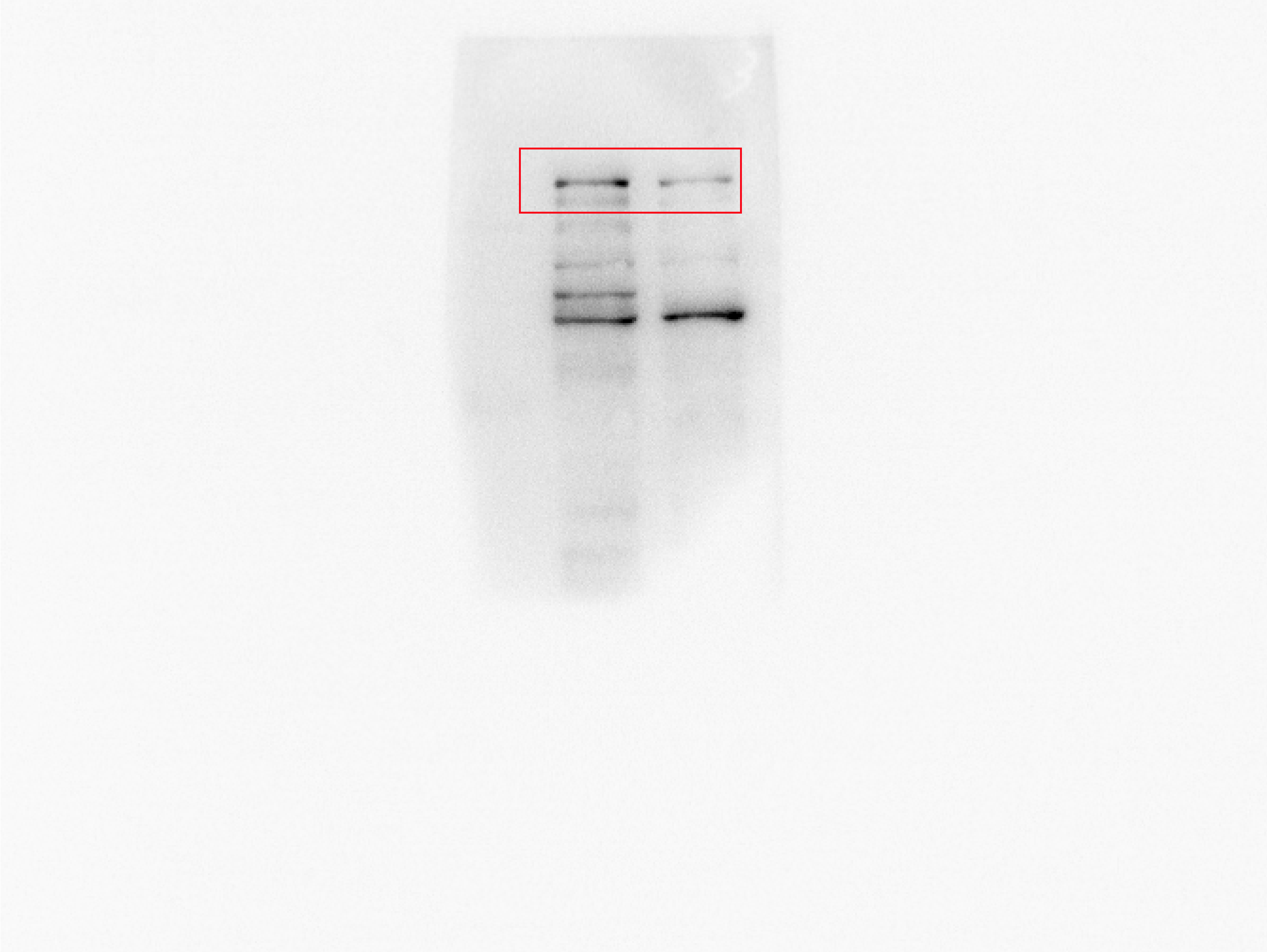

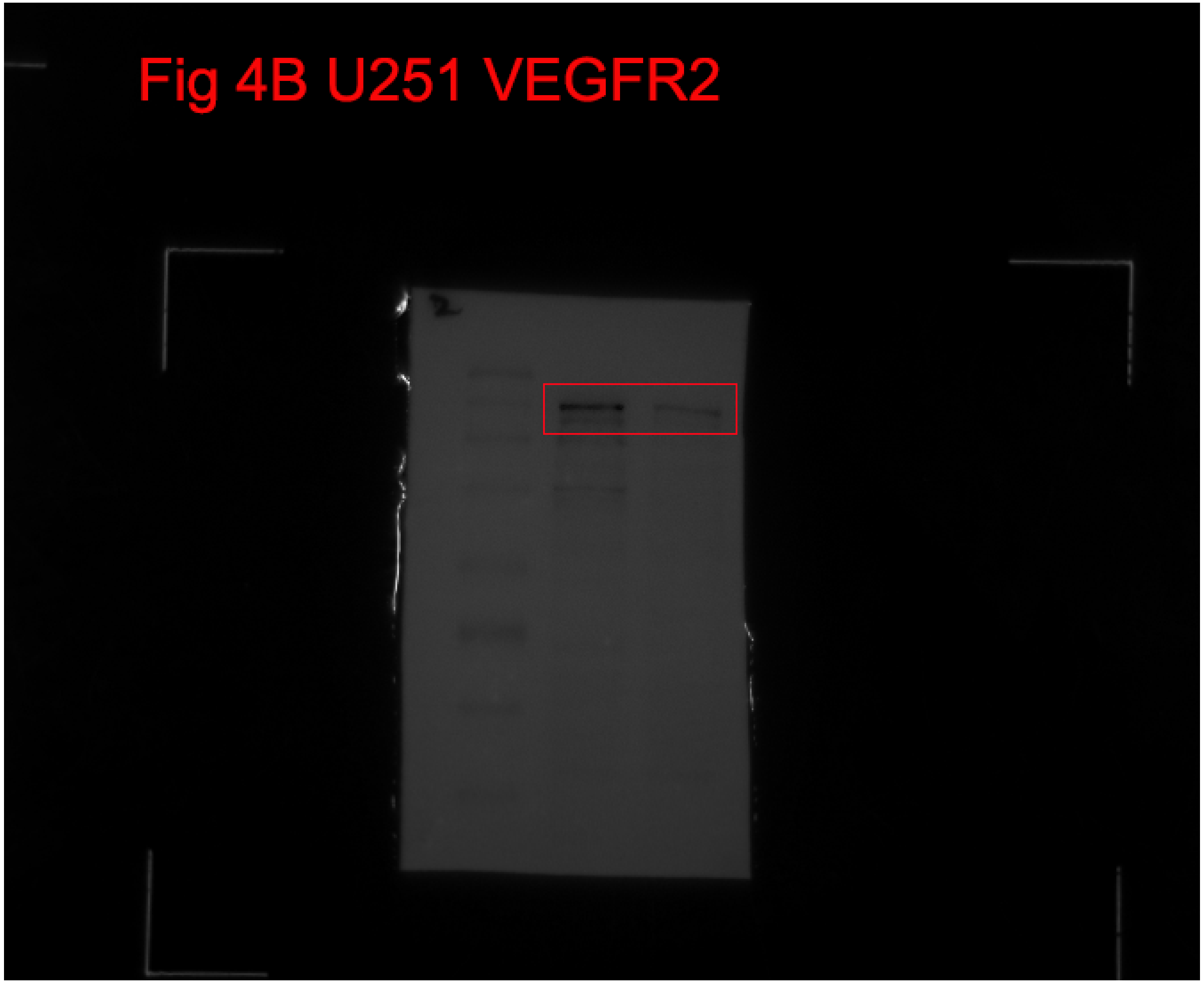
Fig 4B U251 VEGFR2

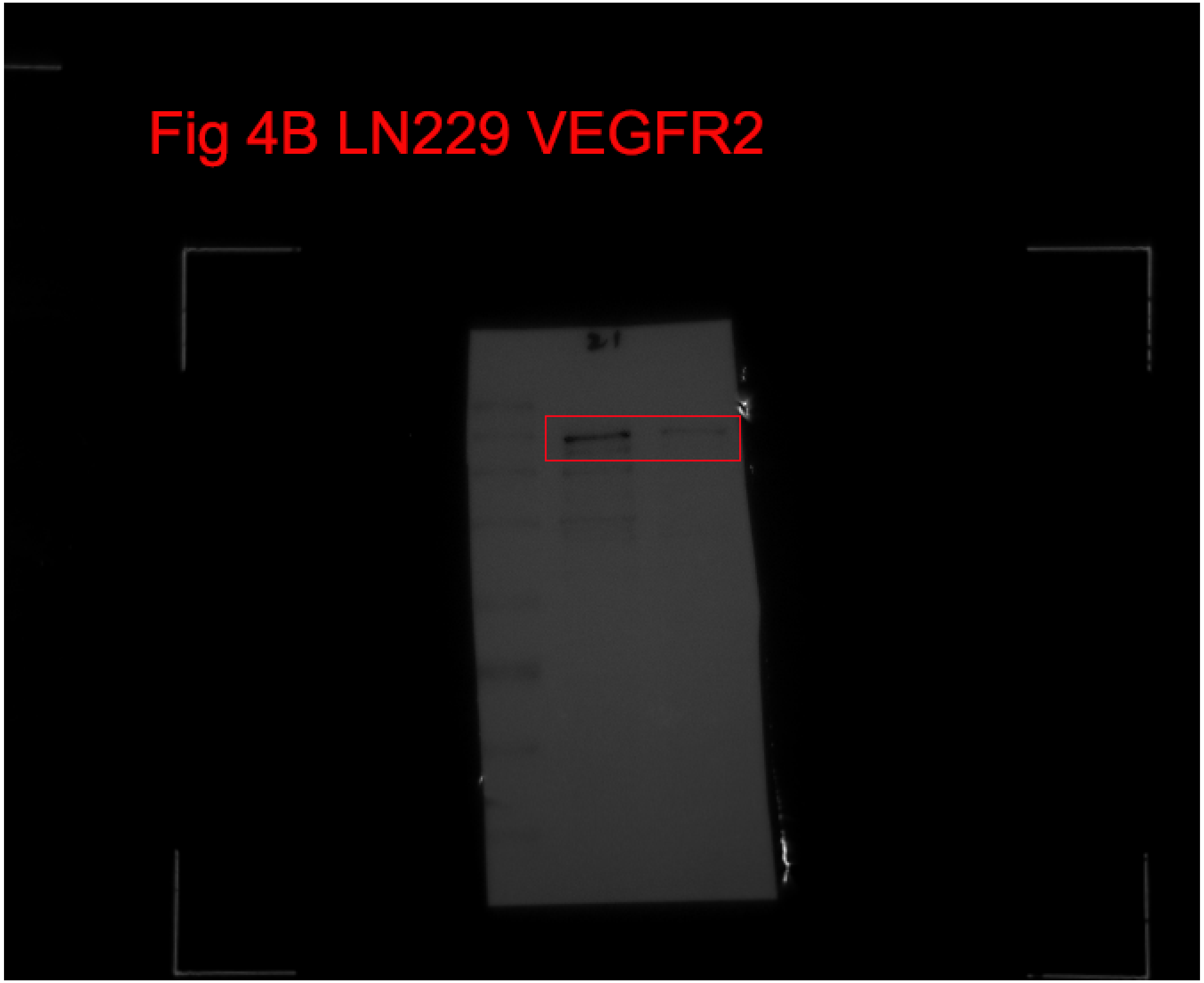
Fig 4B LN229 VEGFR2

Fig 4G LN 229 IP:STAT3

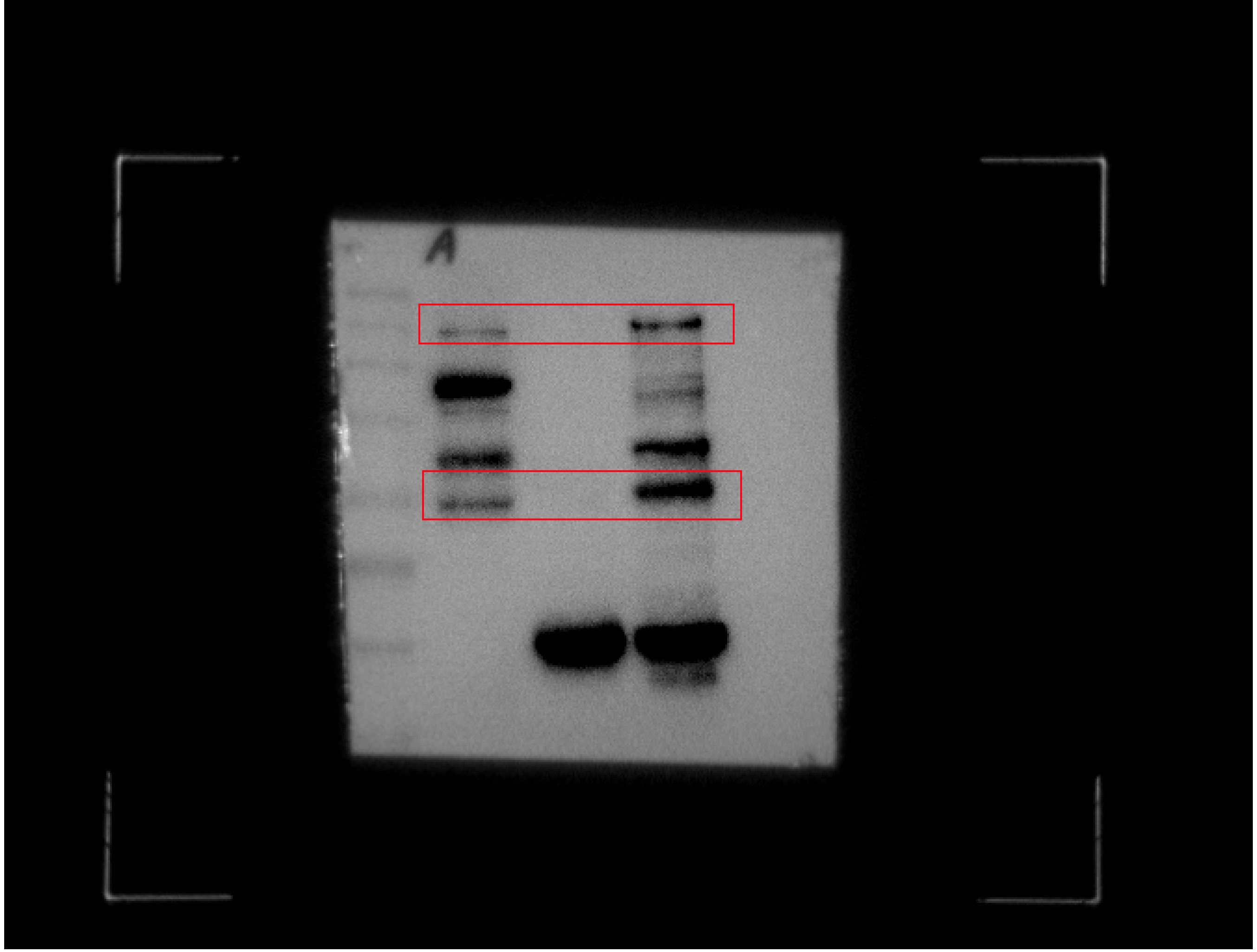

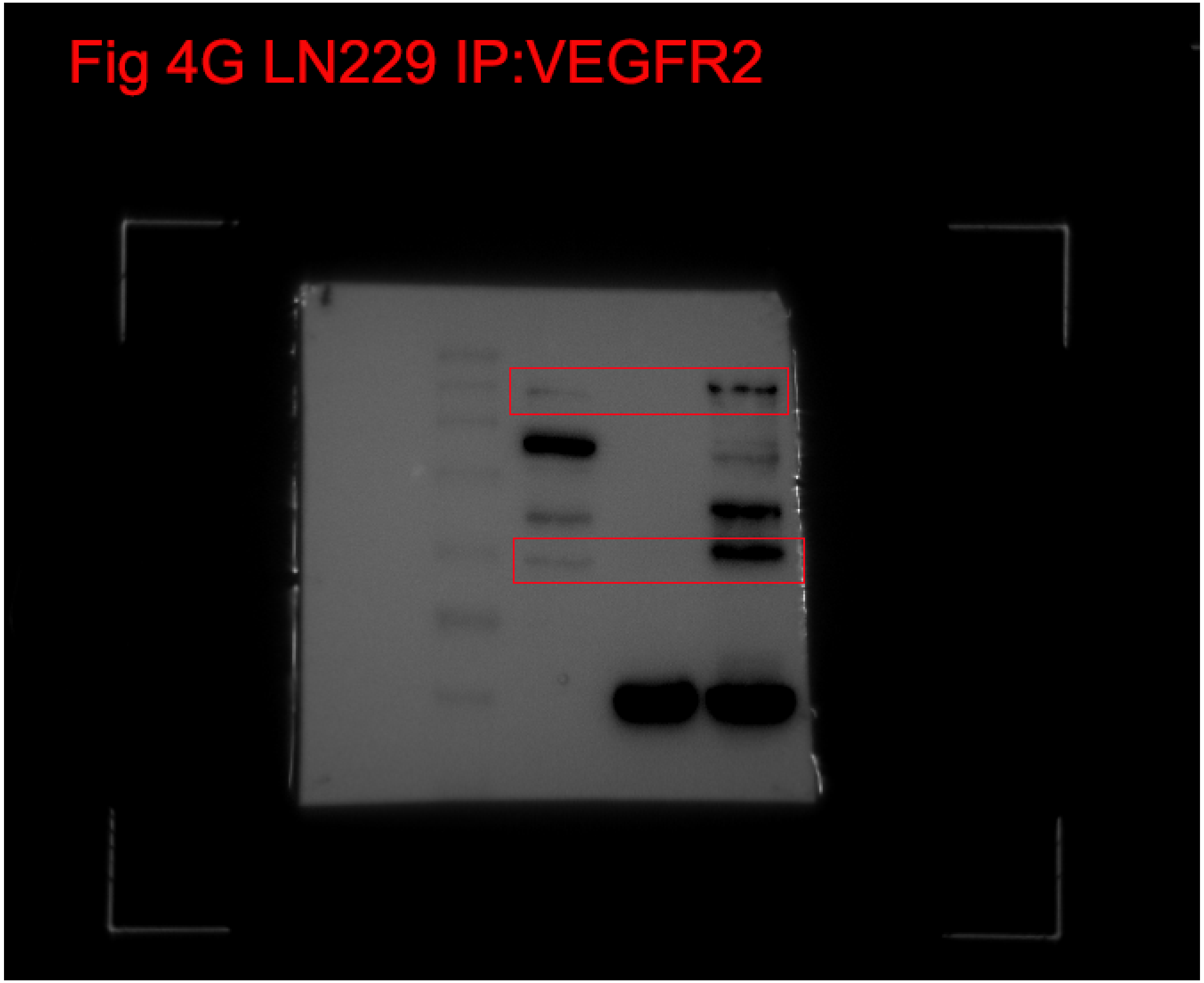
Fig 4G LN229 IP:VEGFR2

Fig 4G LN229 IP:VEGFR2

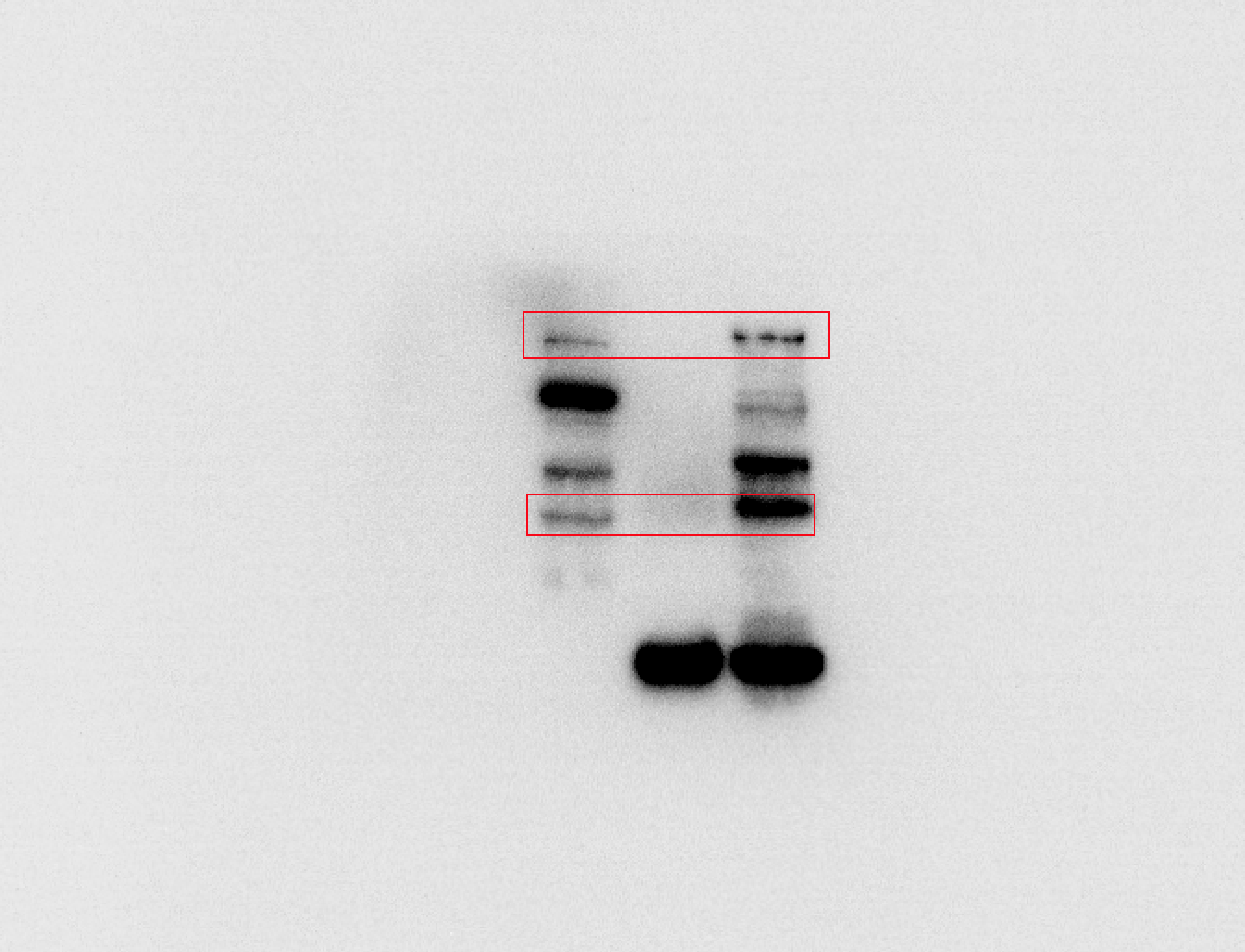

Fig 4G SHG141 IP:VEGFR2

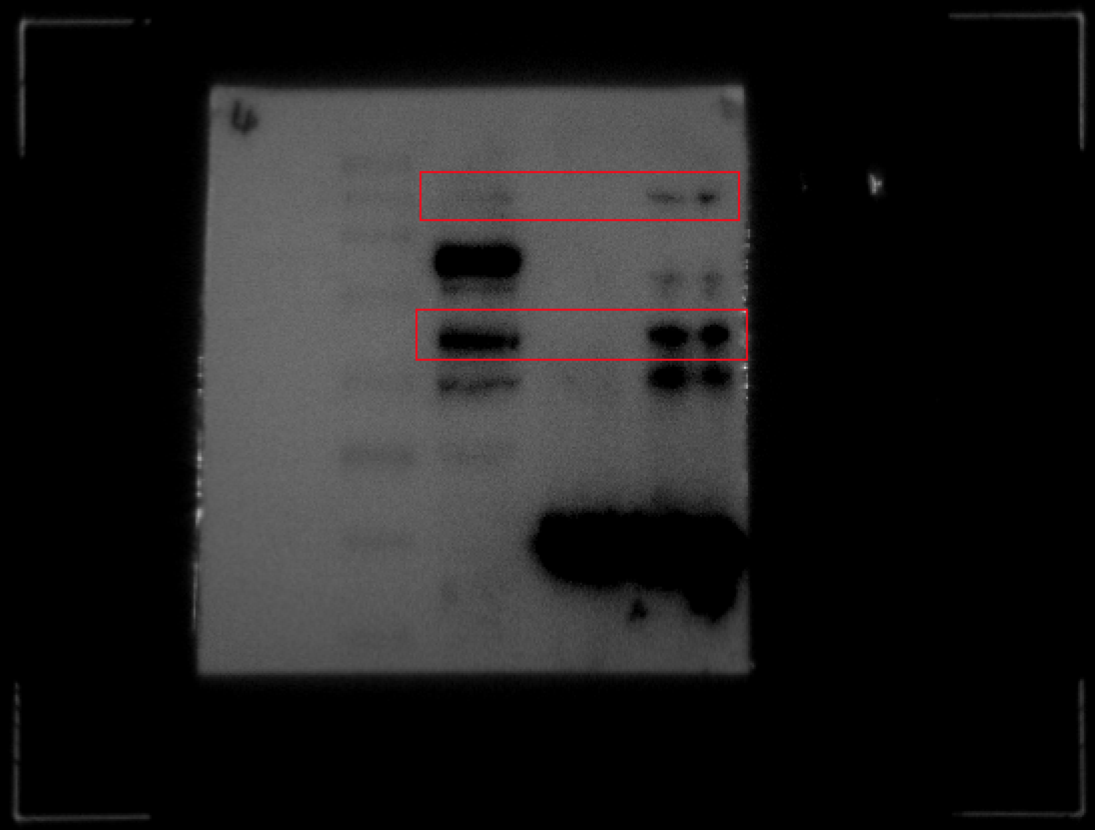

Fig 4G SHG141 IP:STAT3

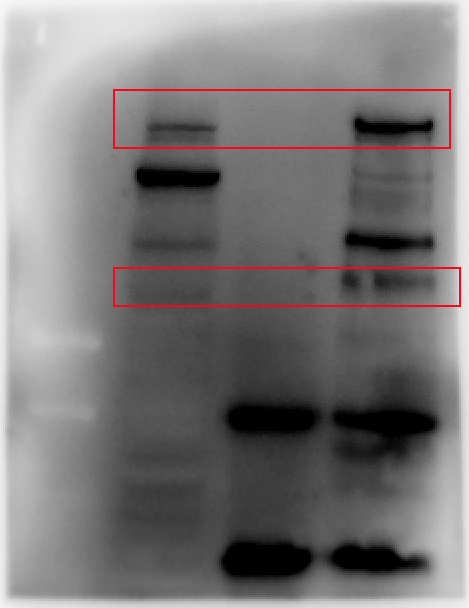

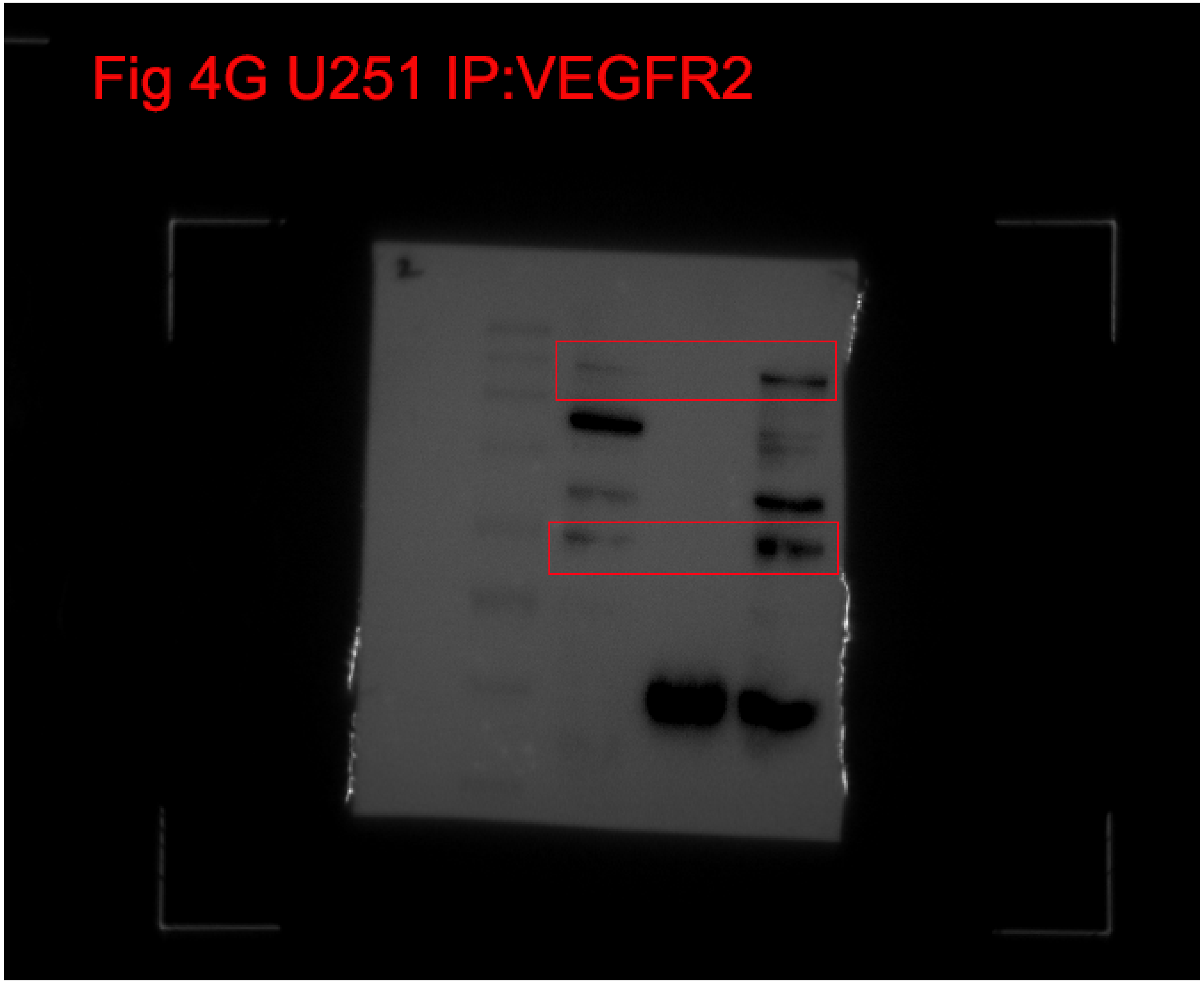
Fig 4G U251 IP:VEGFR2

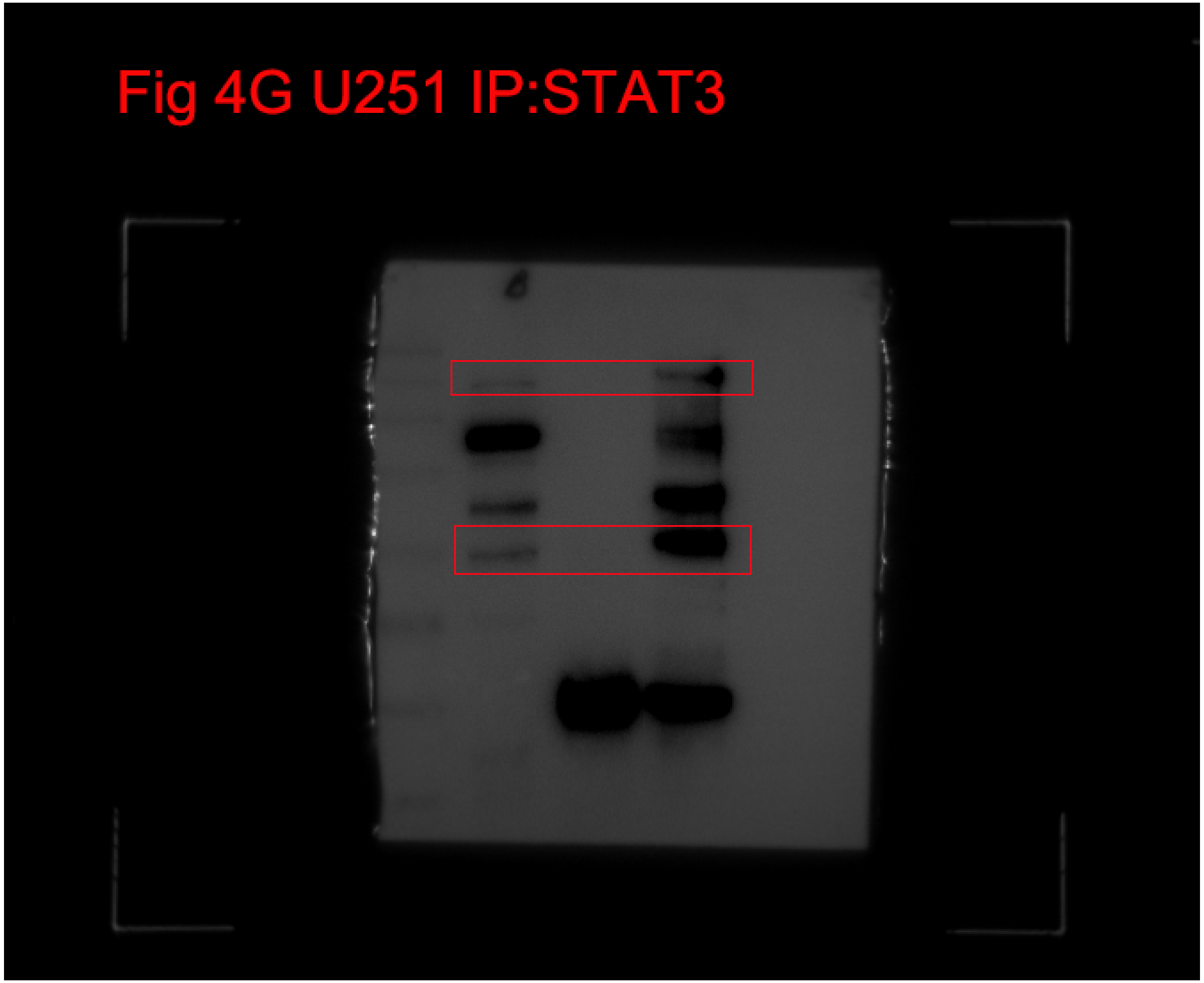
Fig 4G U251 IP:STAT3

Fig 5C LN229 Cleaved CC3

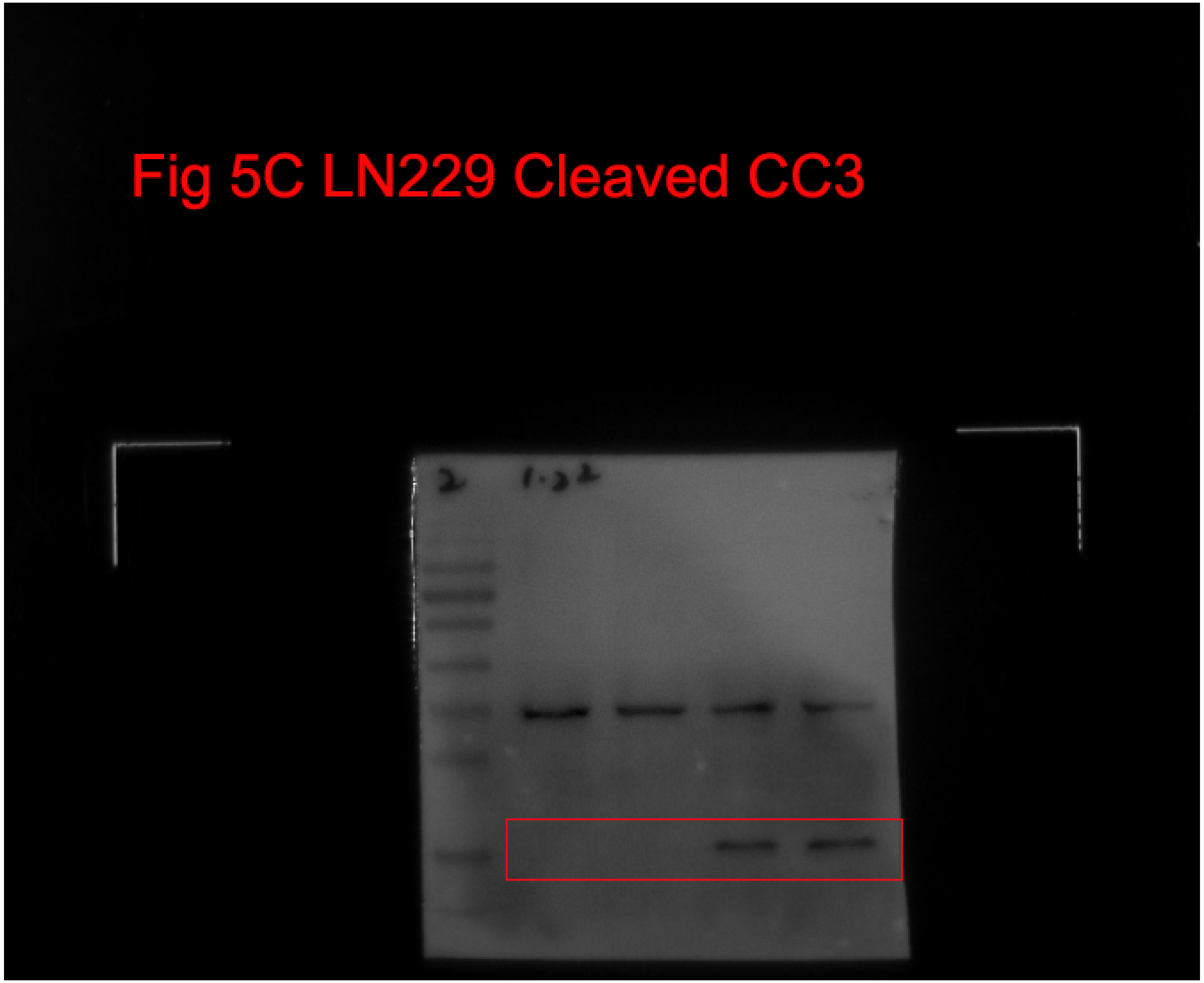

Fig 5C LN229 tubulin

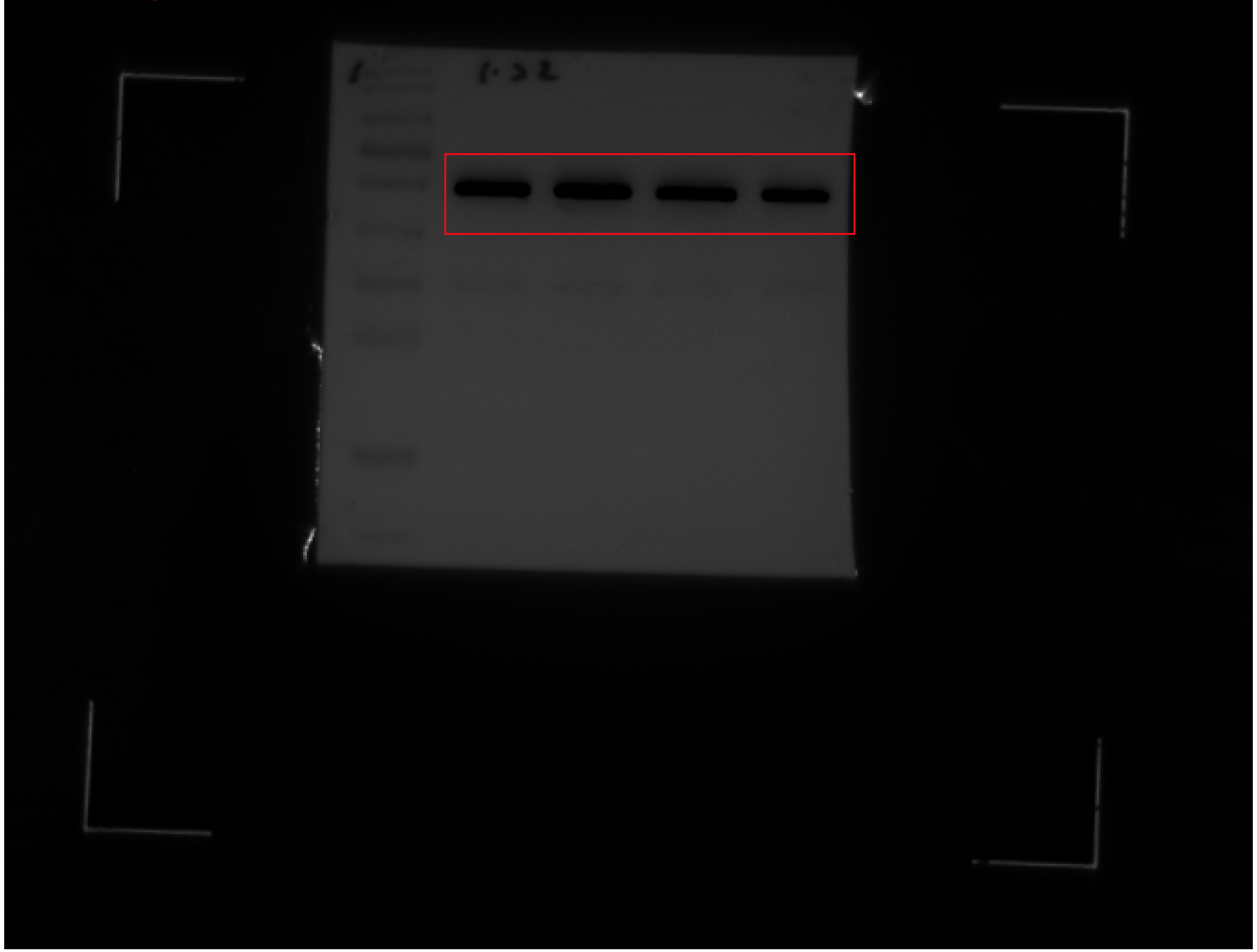

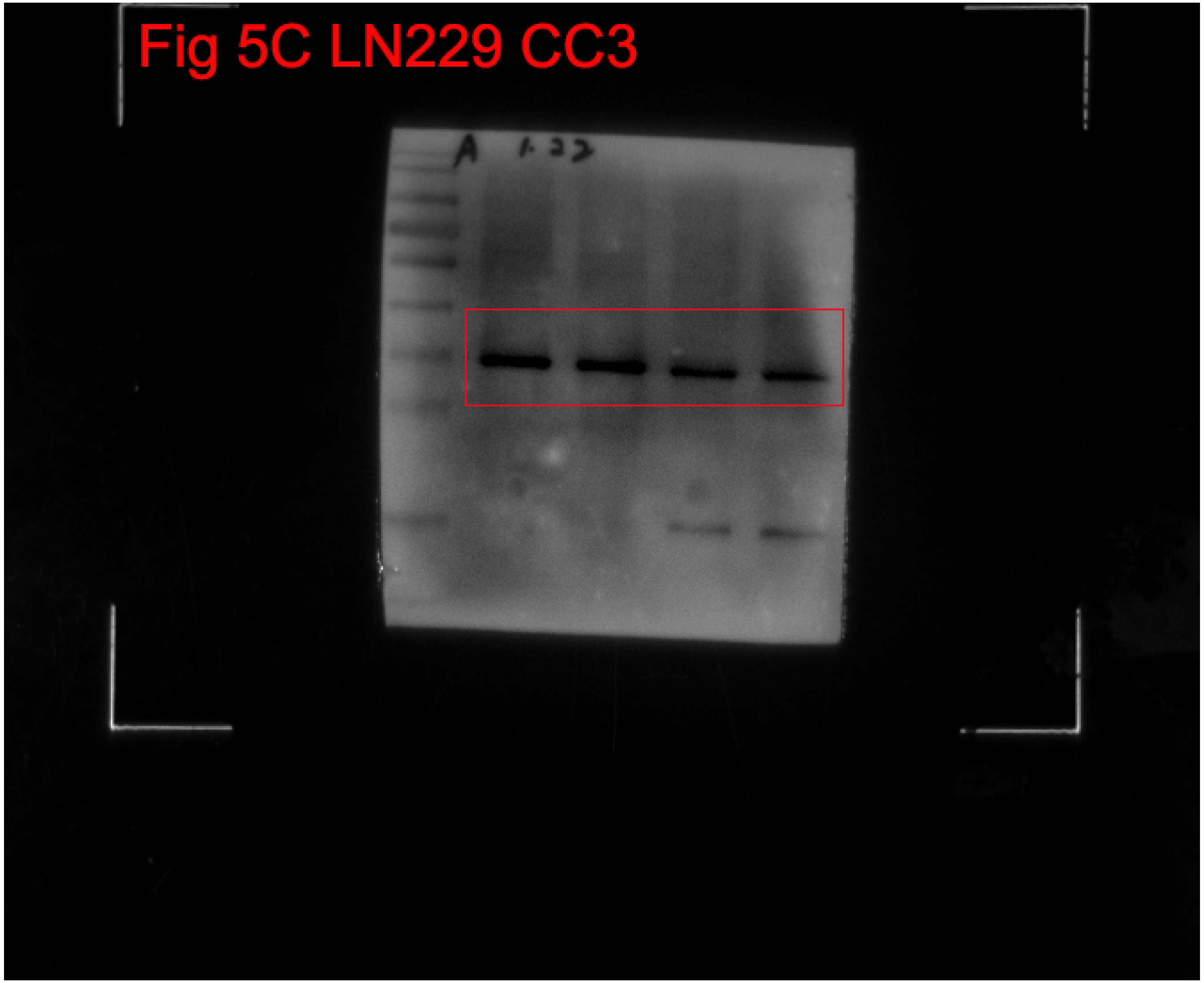
Fig 5C LN229 CC3

Fig 5C SHG141 Cleaved CC3

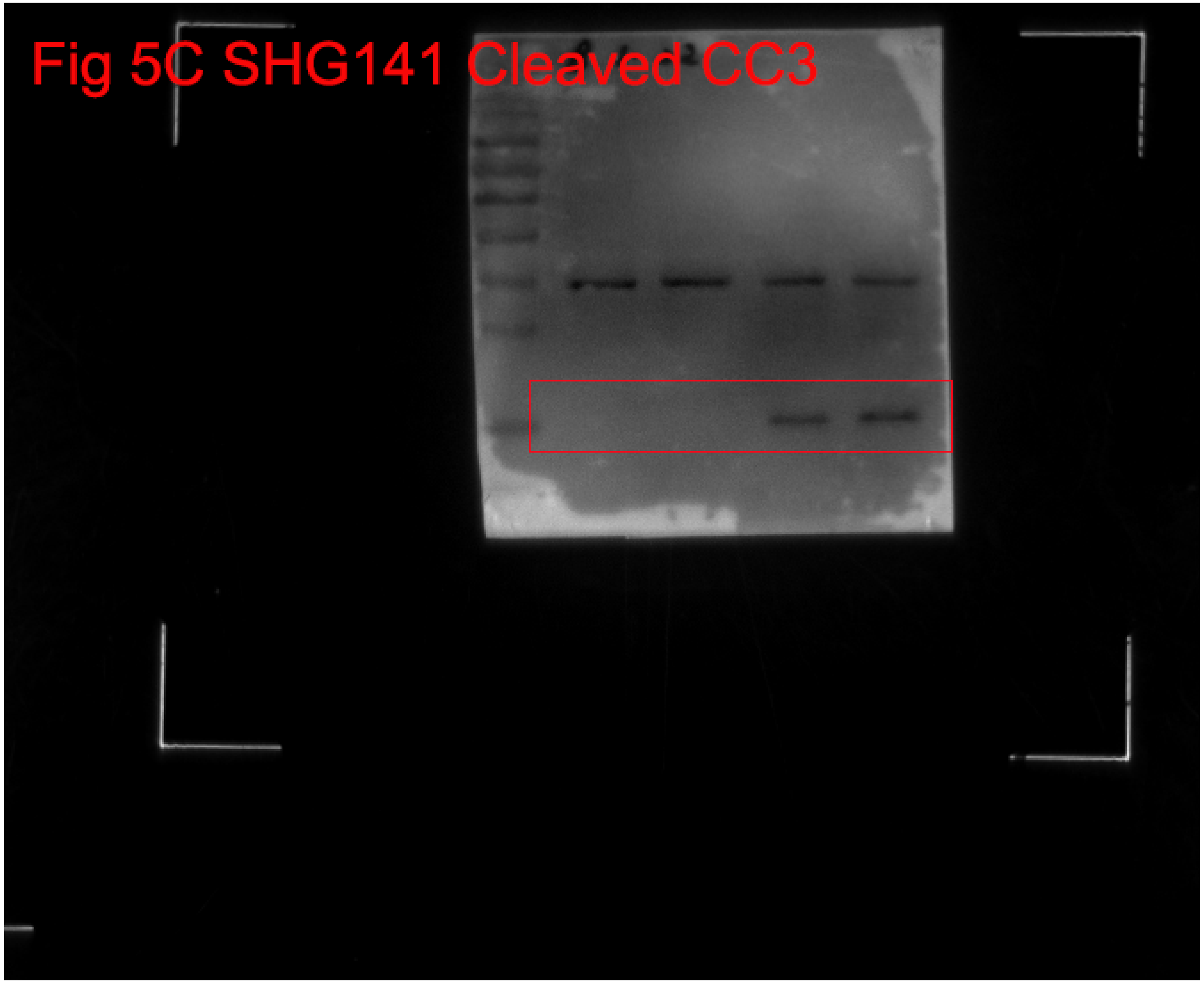

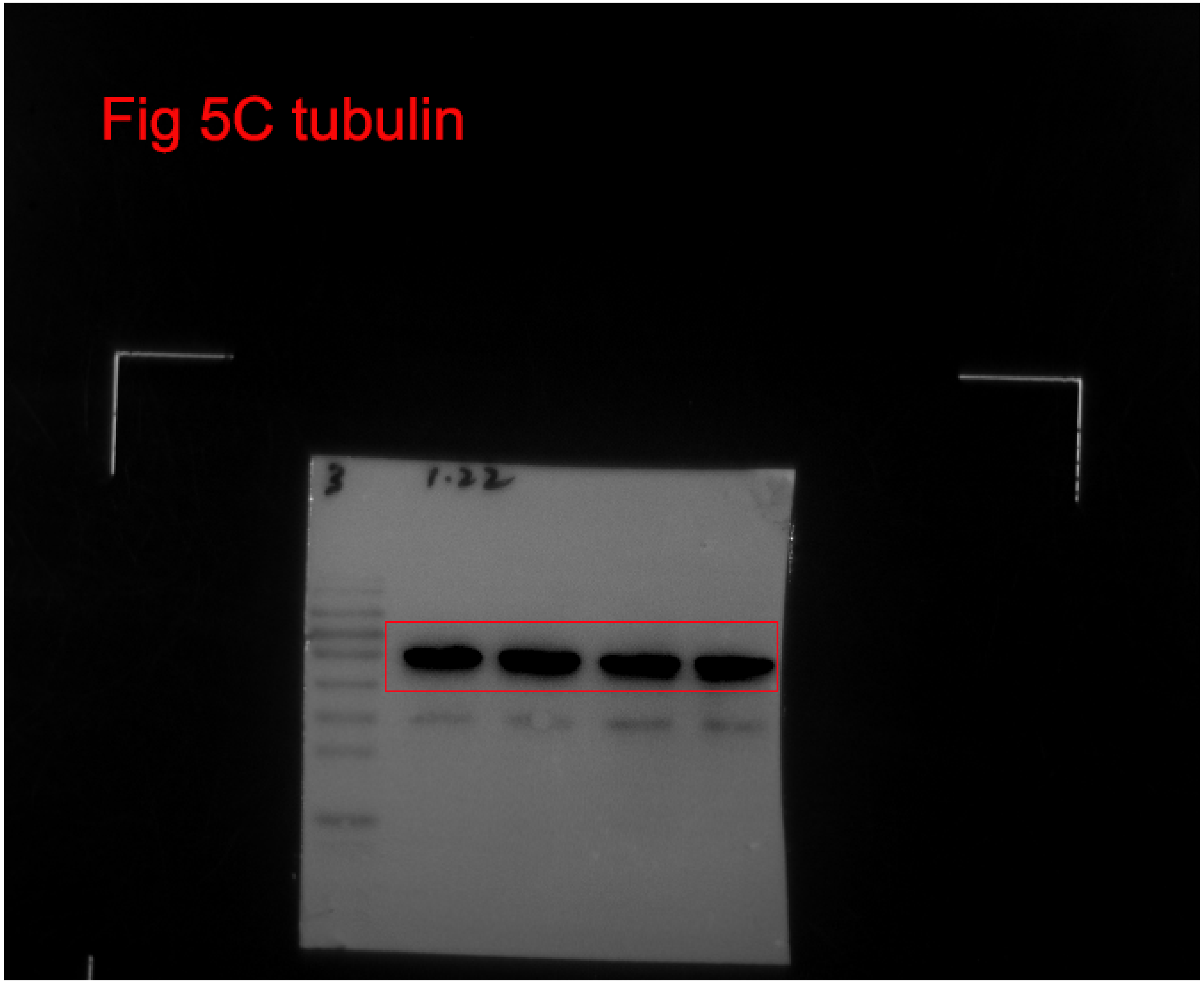
Fig 5C tubulin
3
1.22

Fig 5C SHG141 CC3

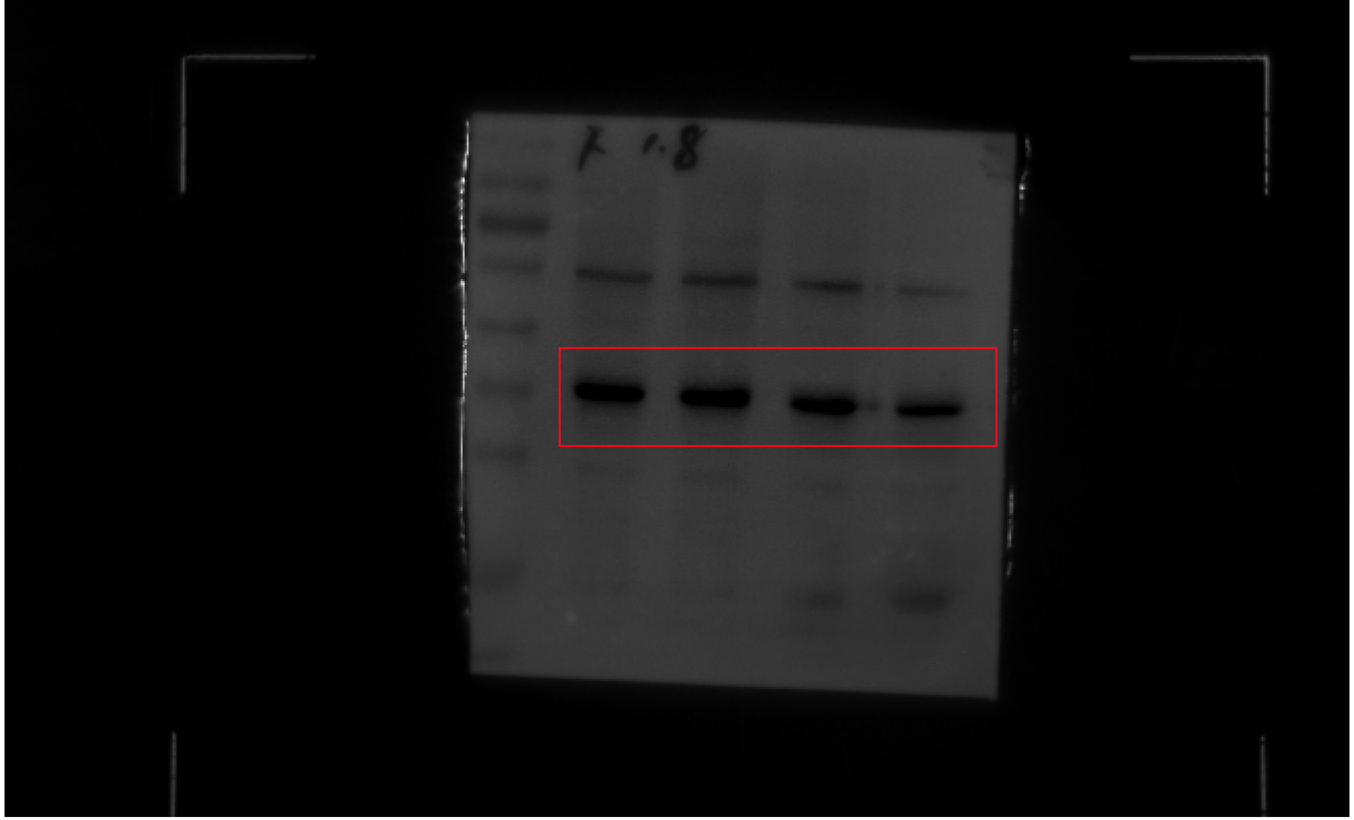

Fig 5C U251 Cleaved CC3

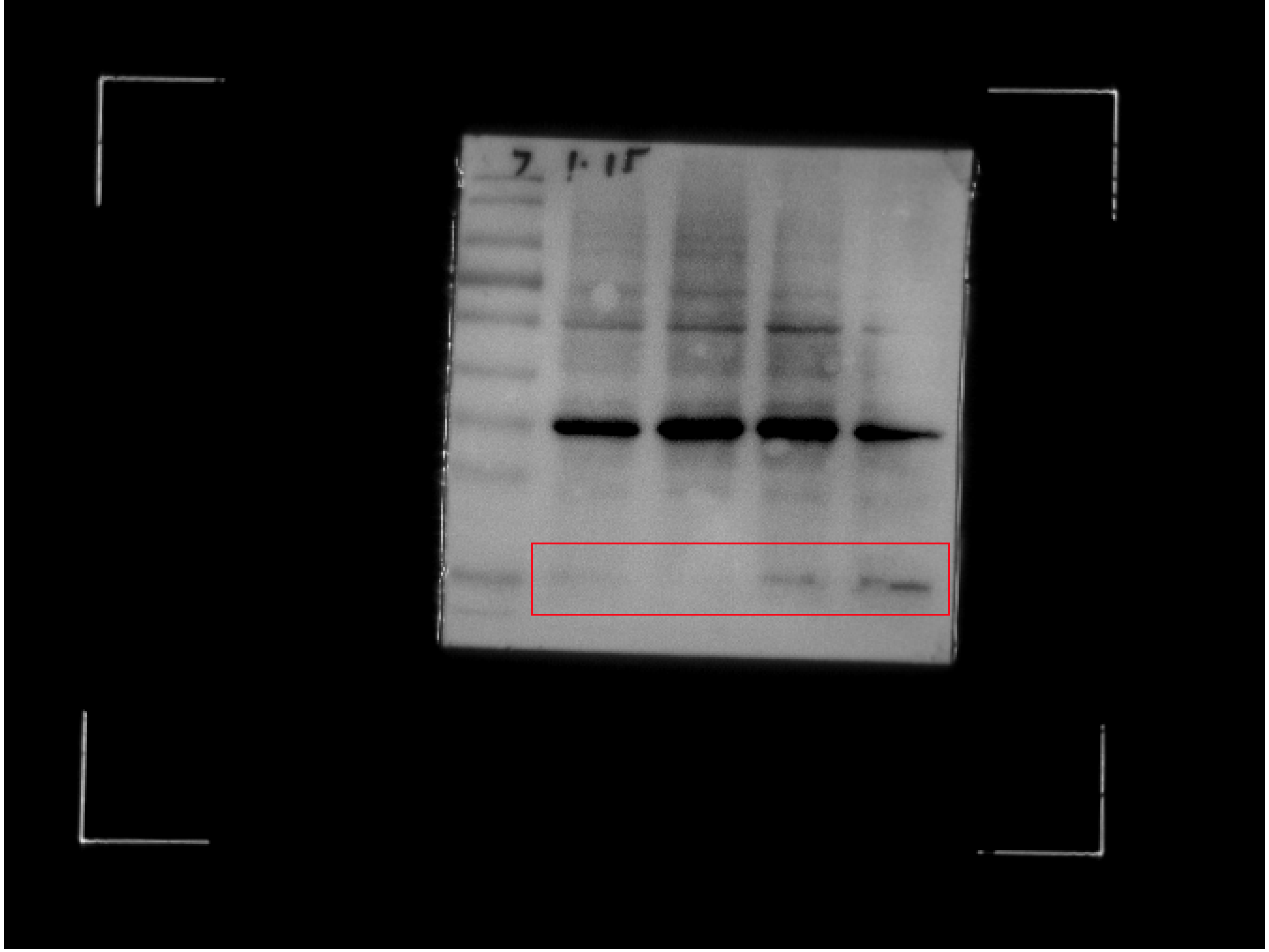

Fig 5C U251 tubulin

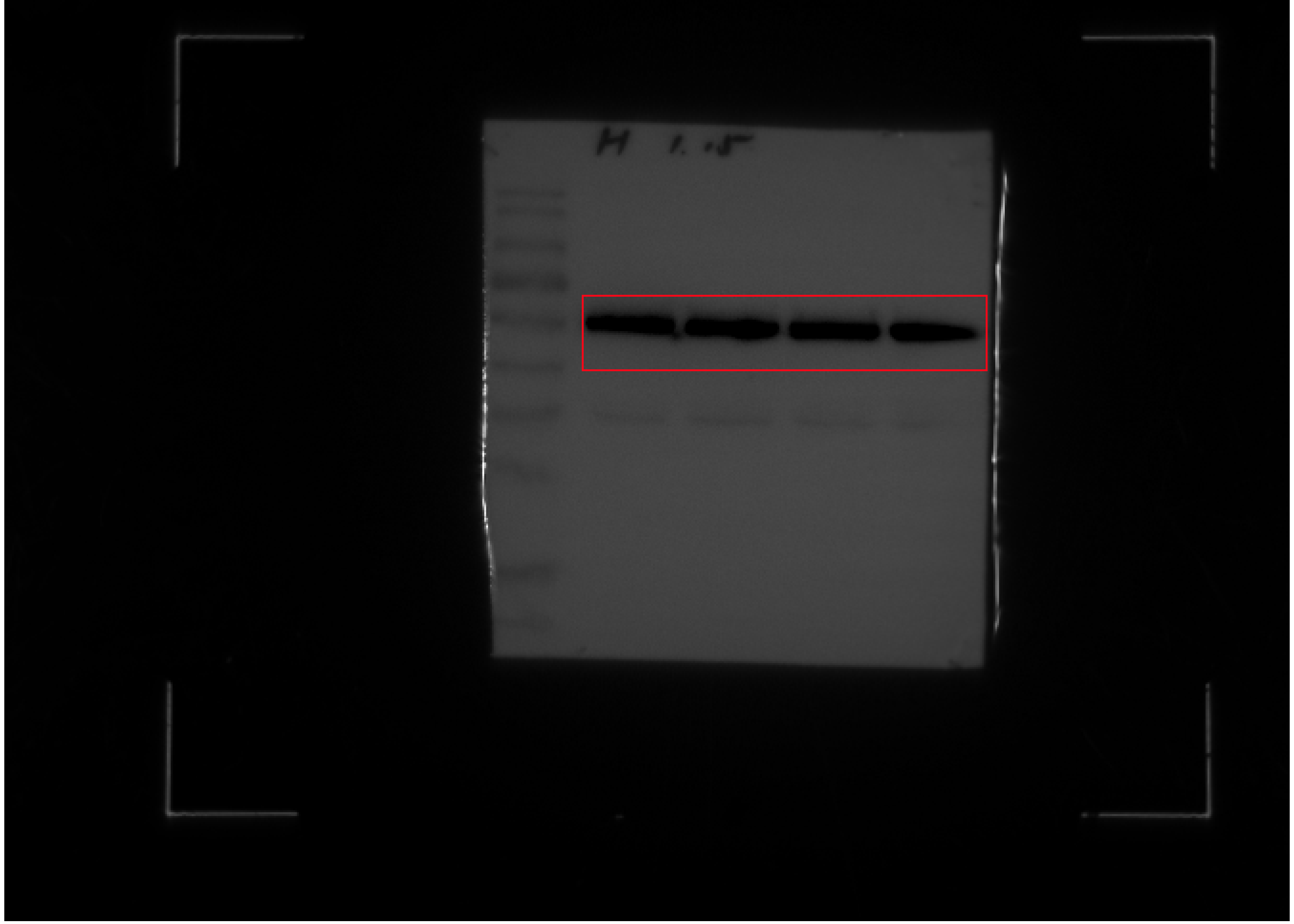

Fig 5C U251 CC3

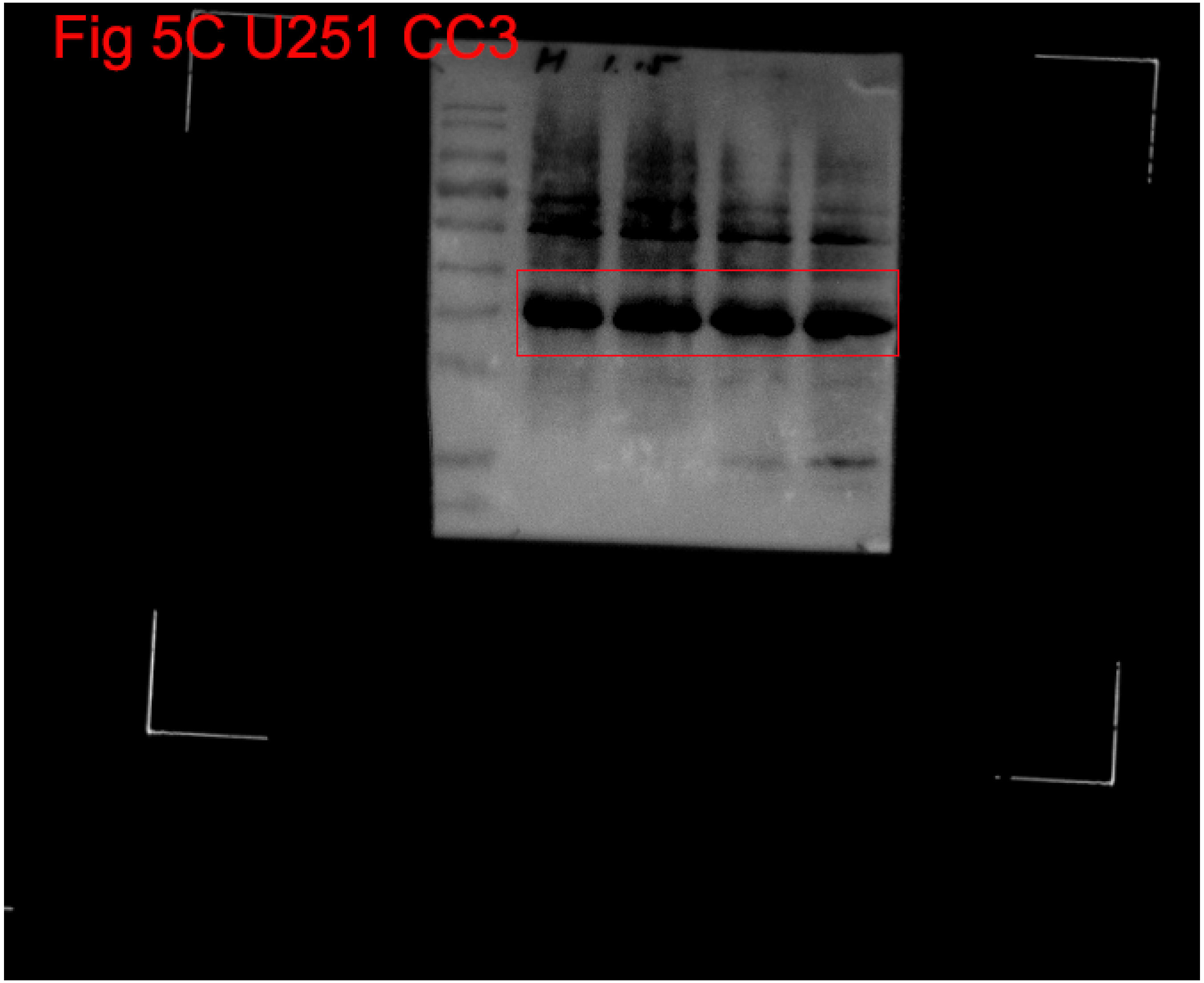

Fig 5H Cleaved PARP and PARP

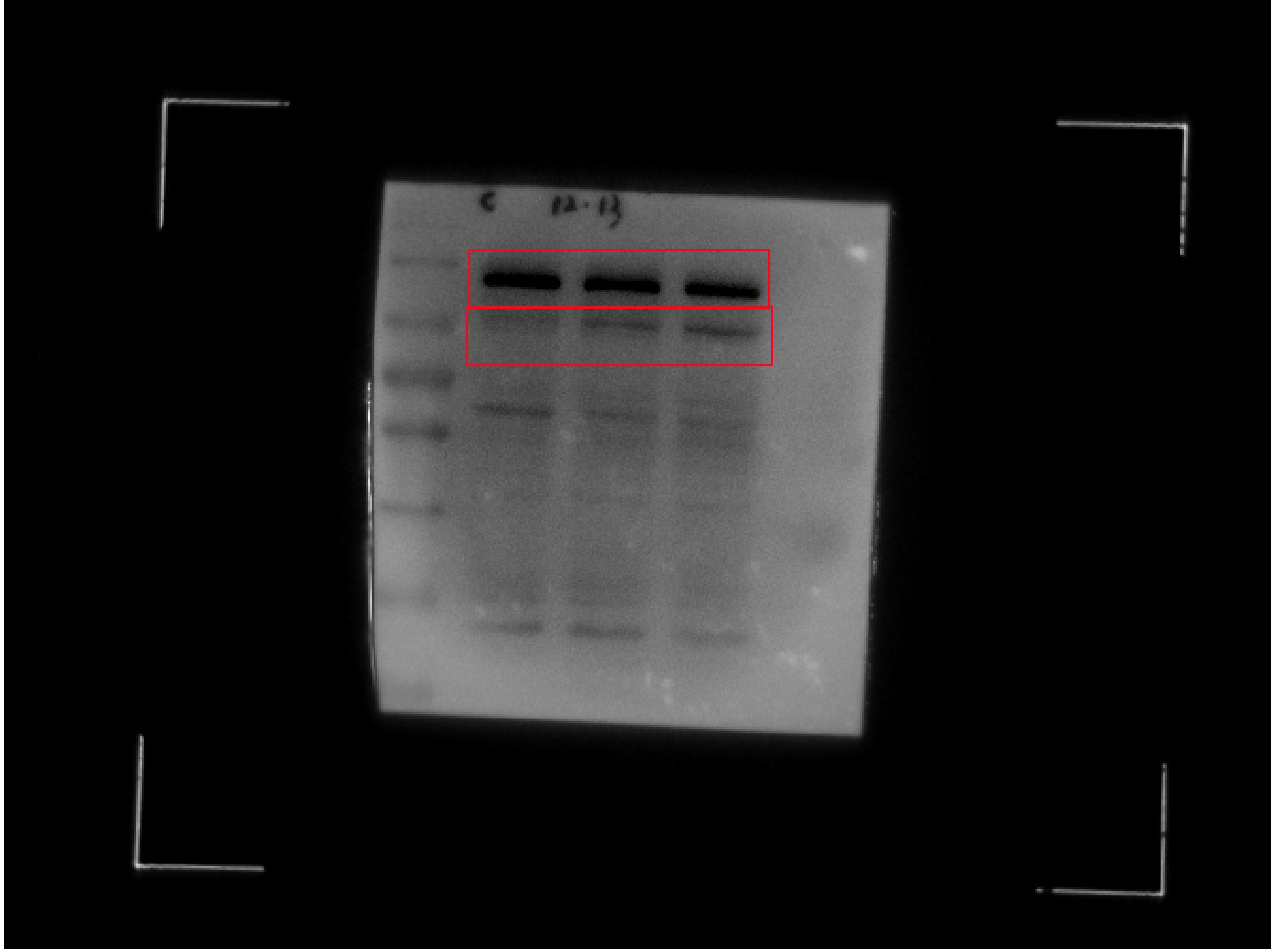

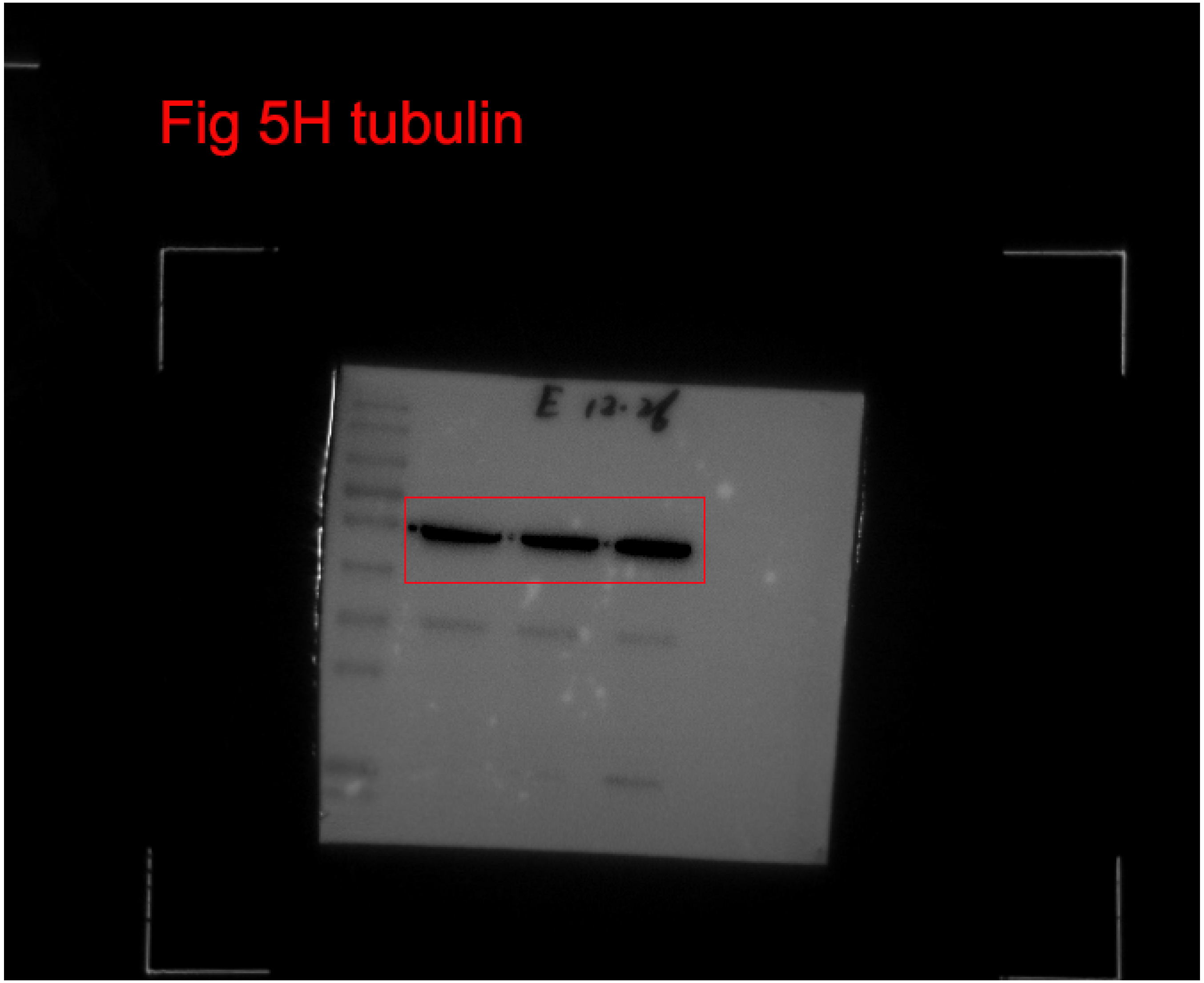
Fig 5H tubulin

Fig 5H LN229 Cleaved CC3 and CC3

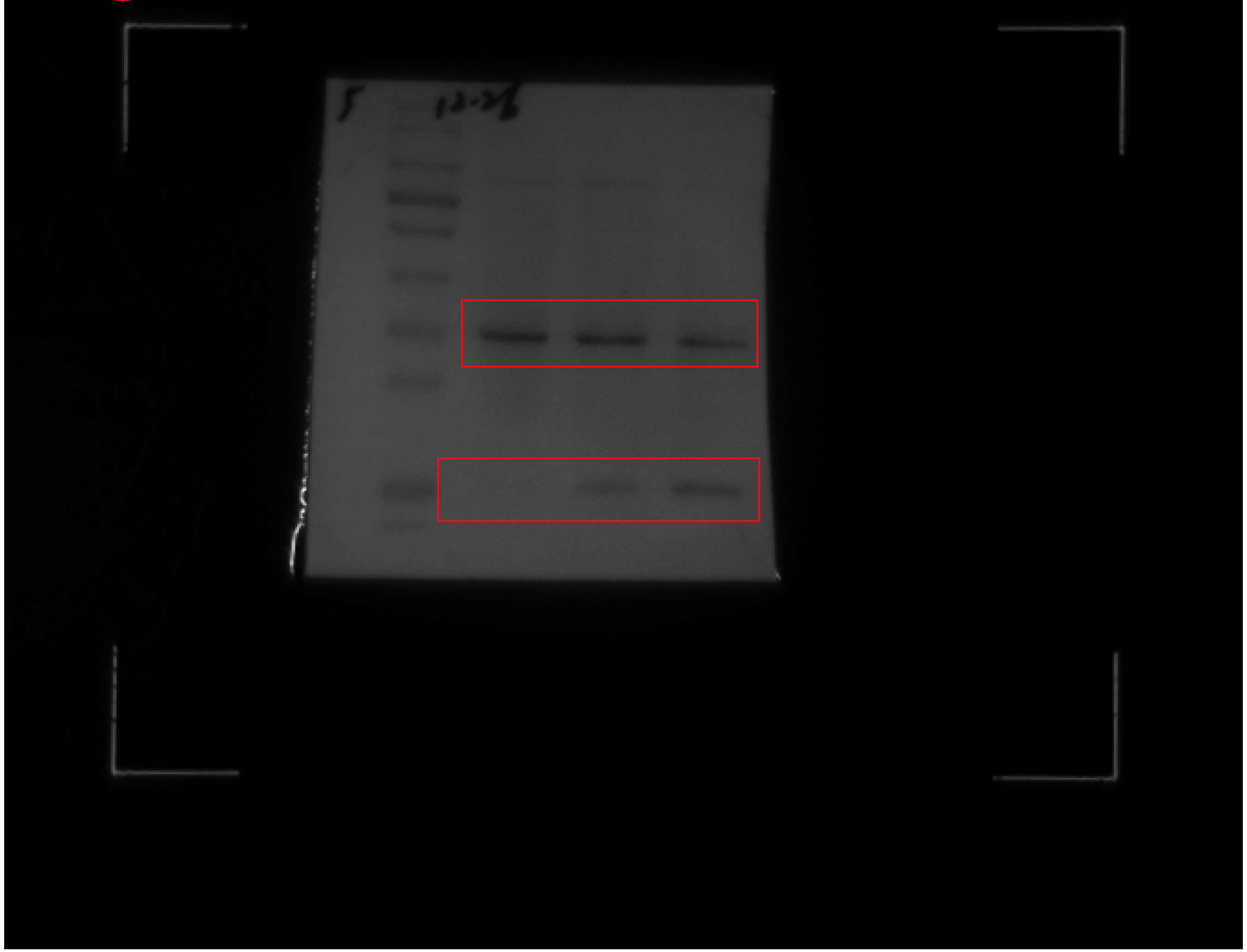

Fig 5H SHG141 Cleaved PARP and PARP

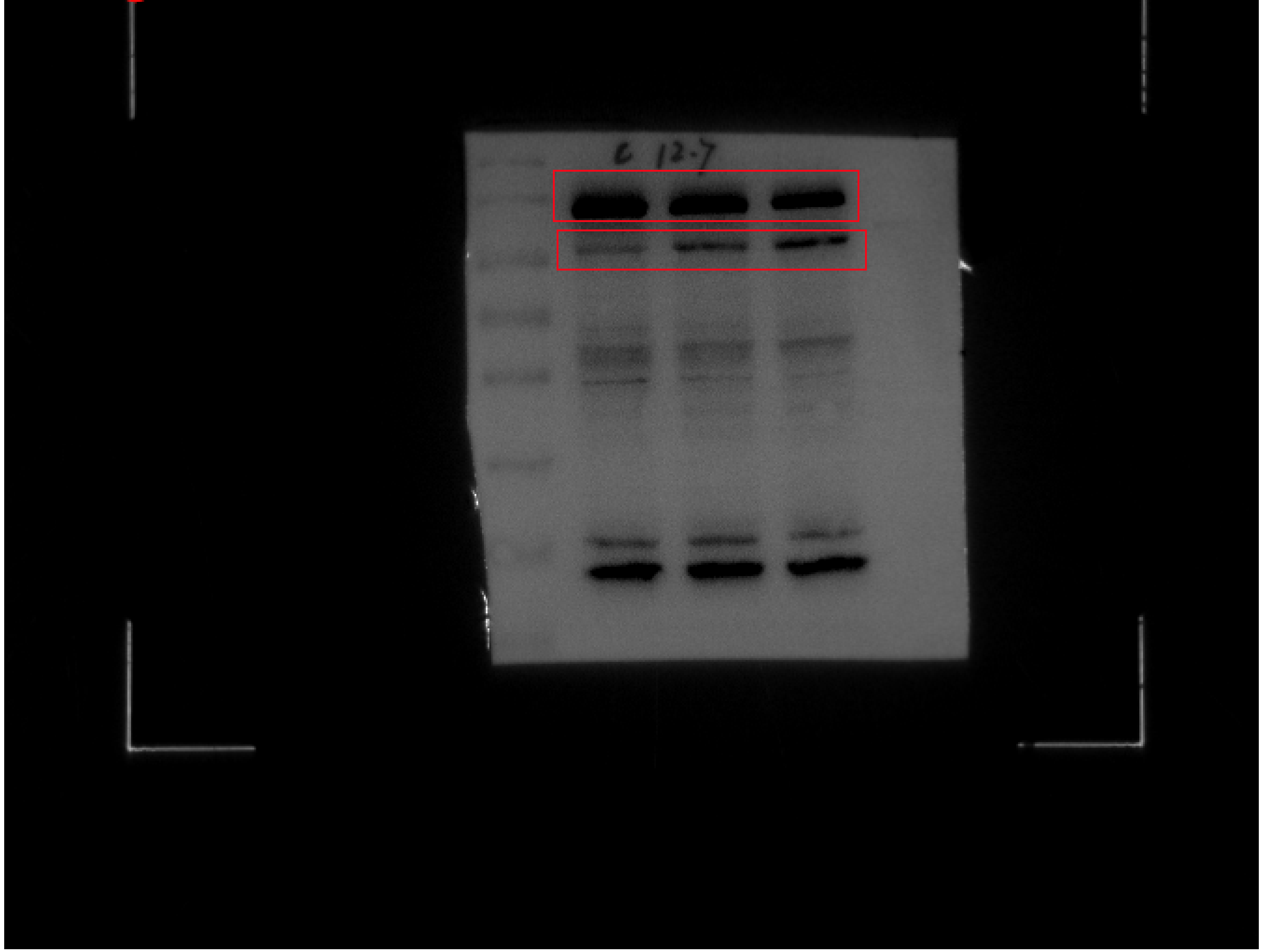

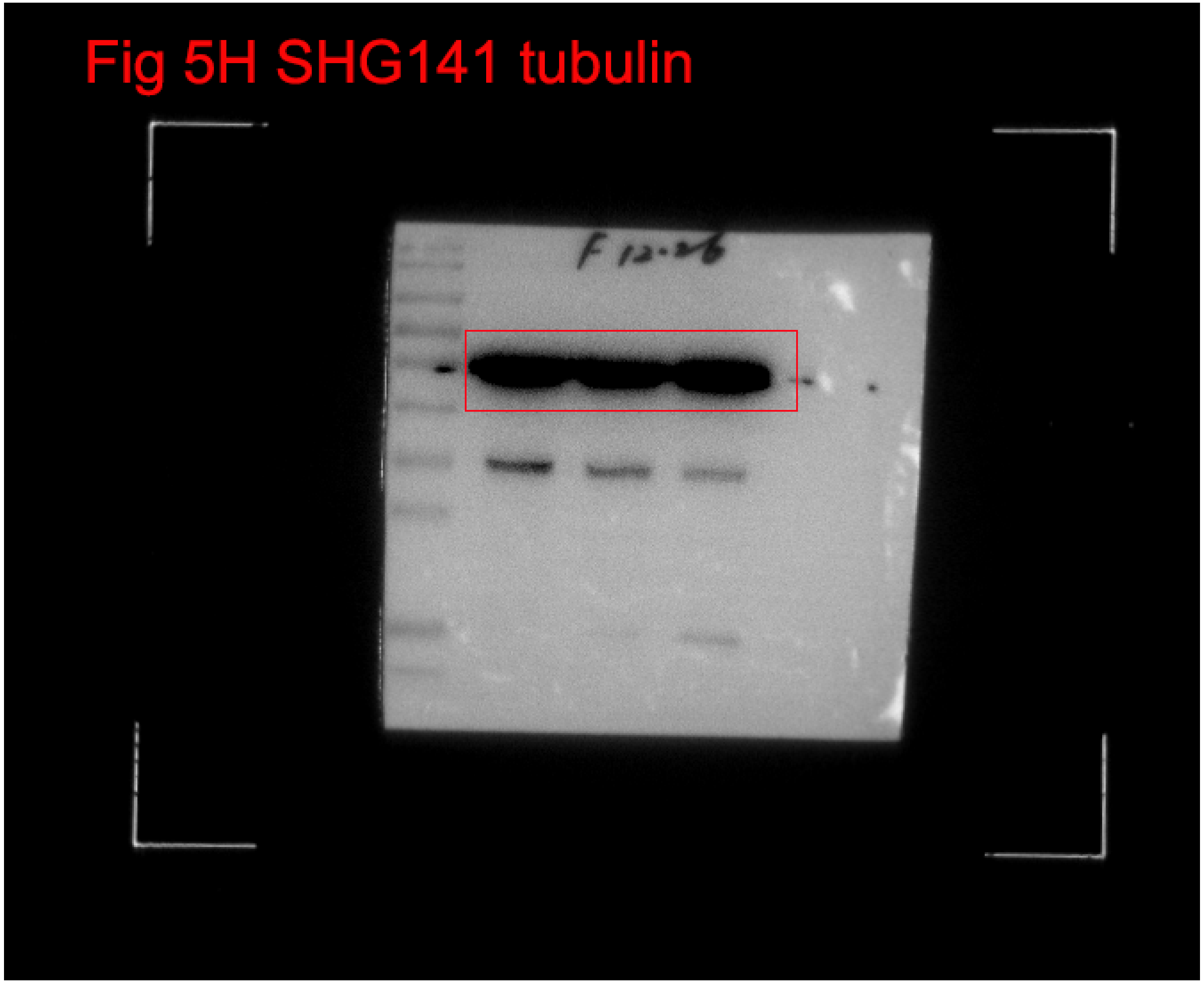
Fig 5H SHG141 tubulin

Fig 5H SHG141Cleaved CC3 and CC3

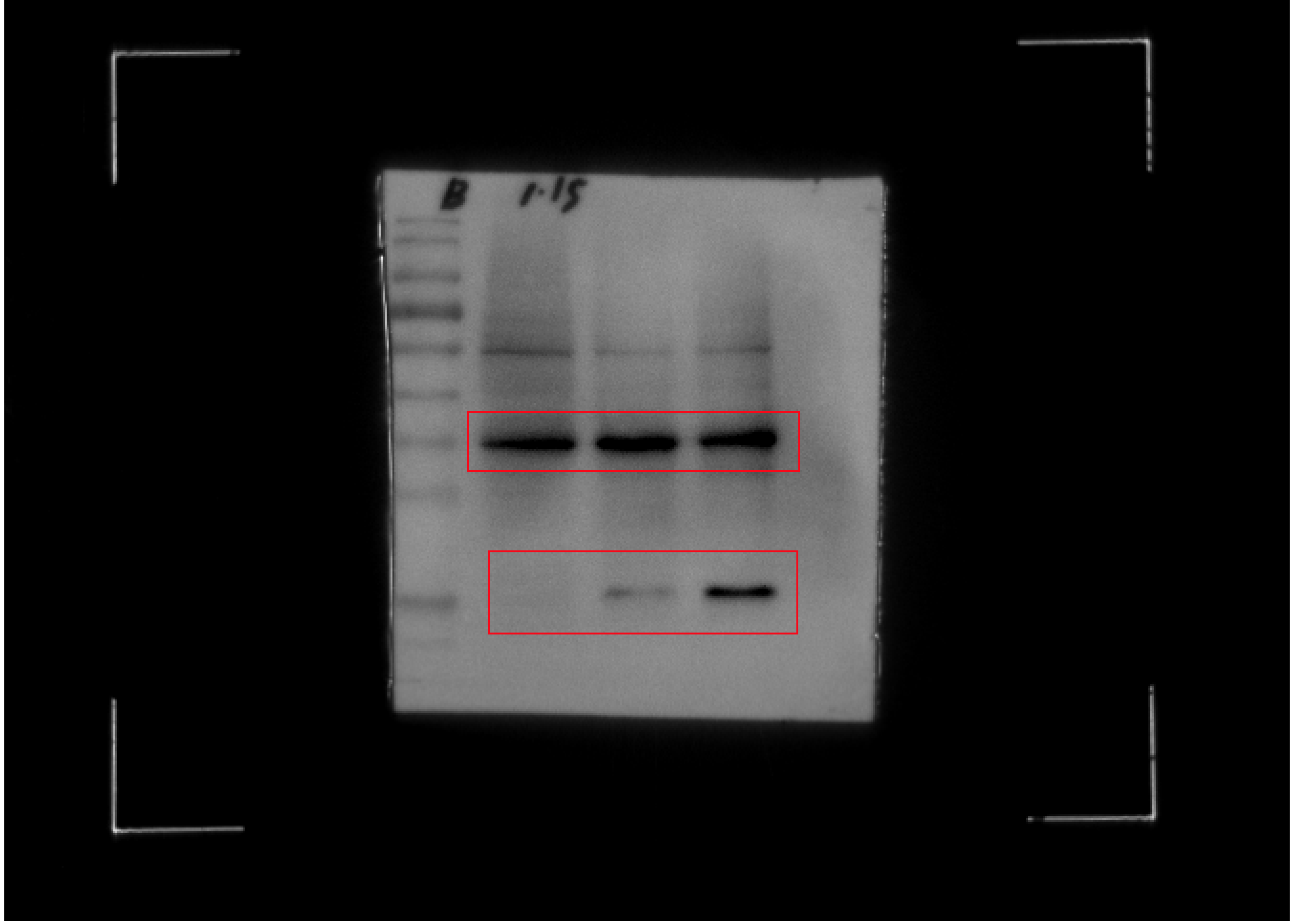

Fig 5H U251 Cleaved PARP and PARP

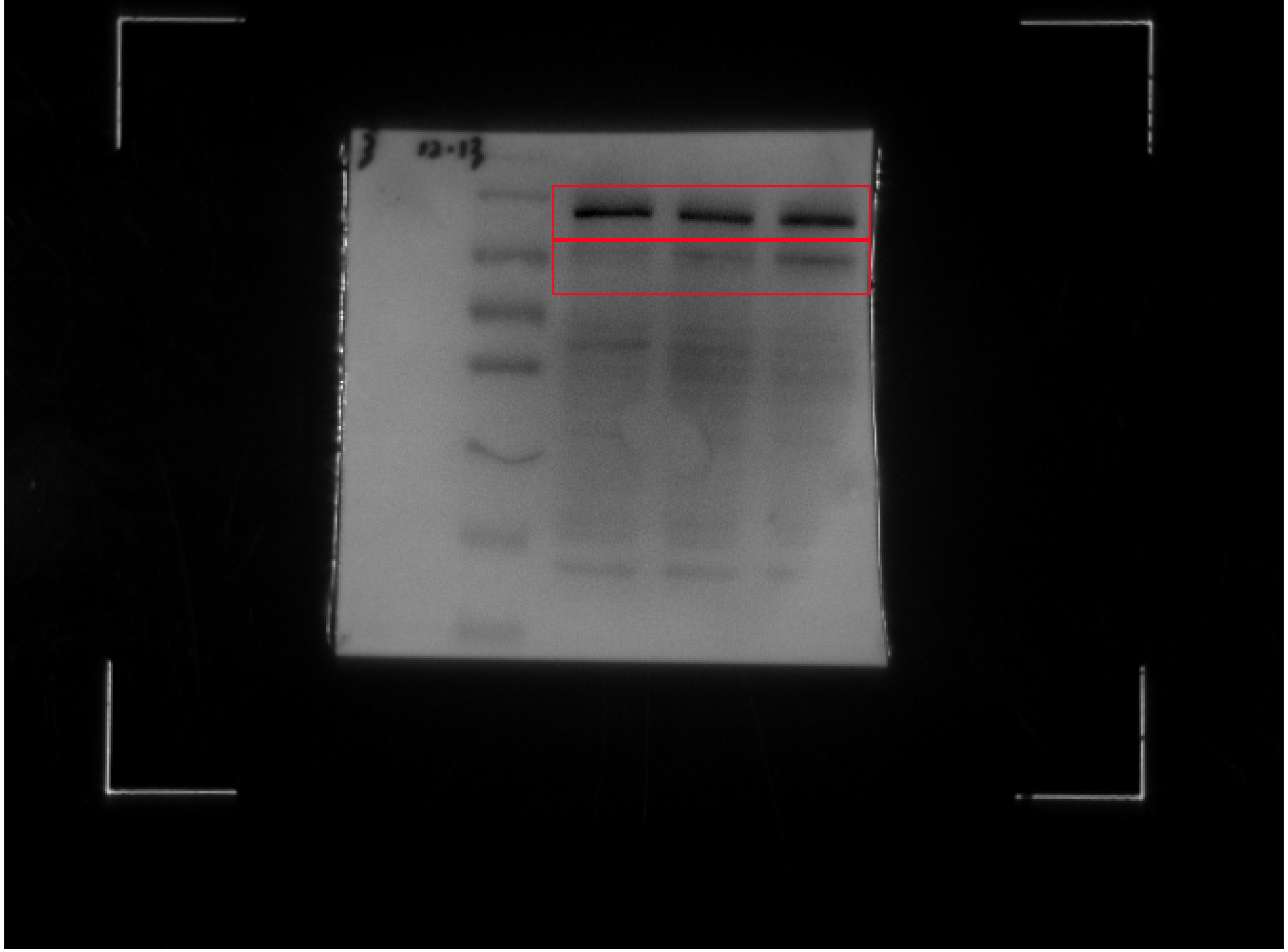

Fig 5H tubulin

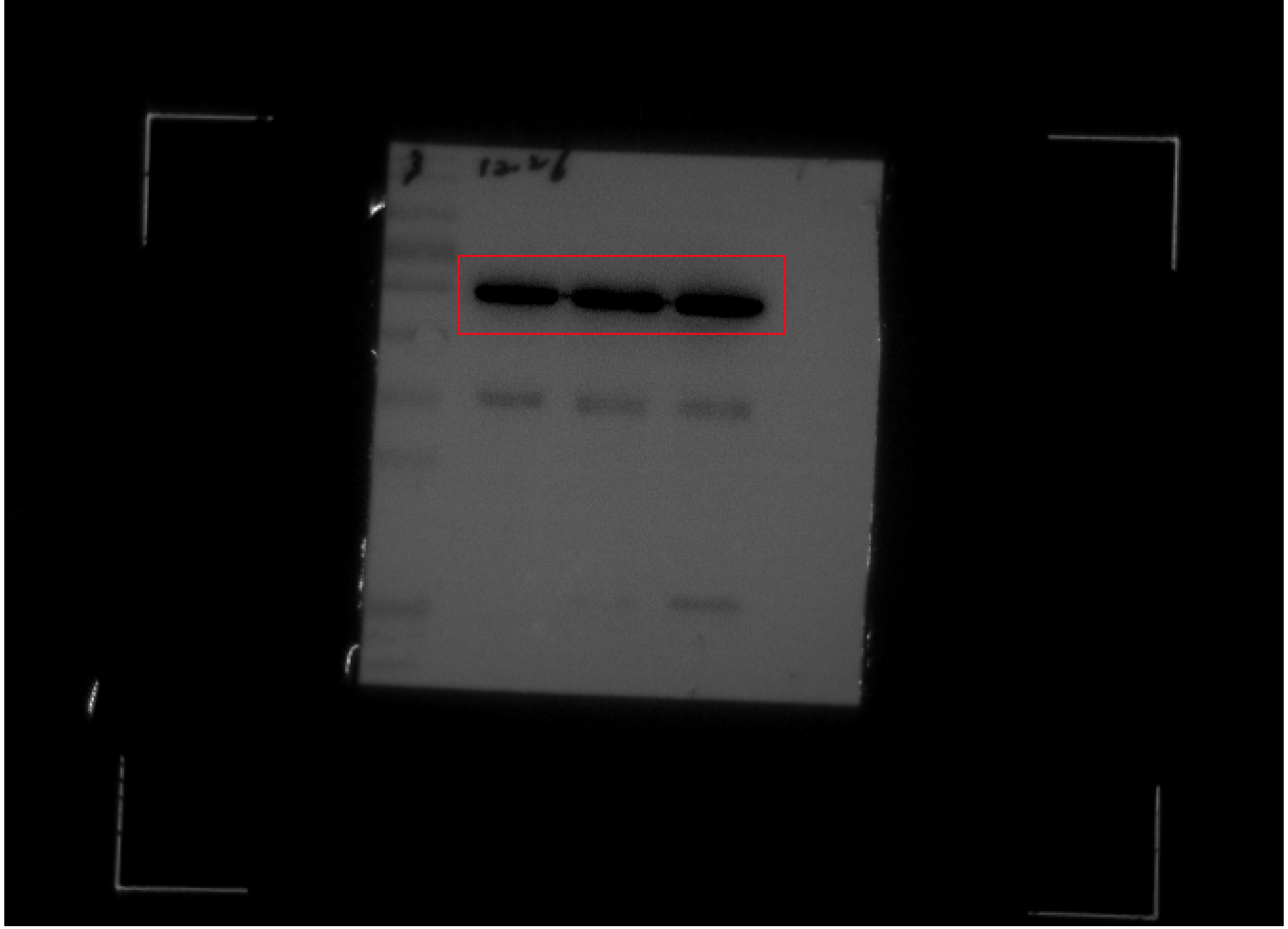

Fig 5H U251 Cleaved CC3 and CC3

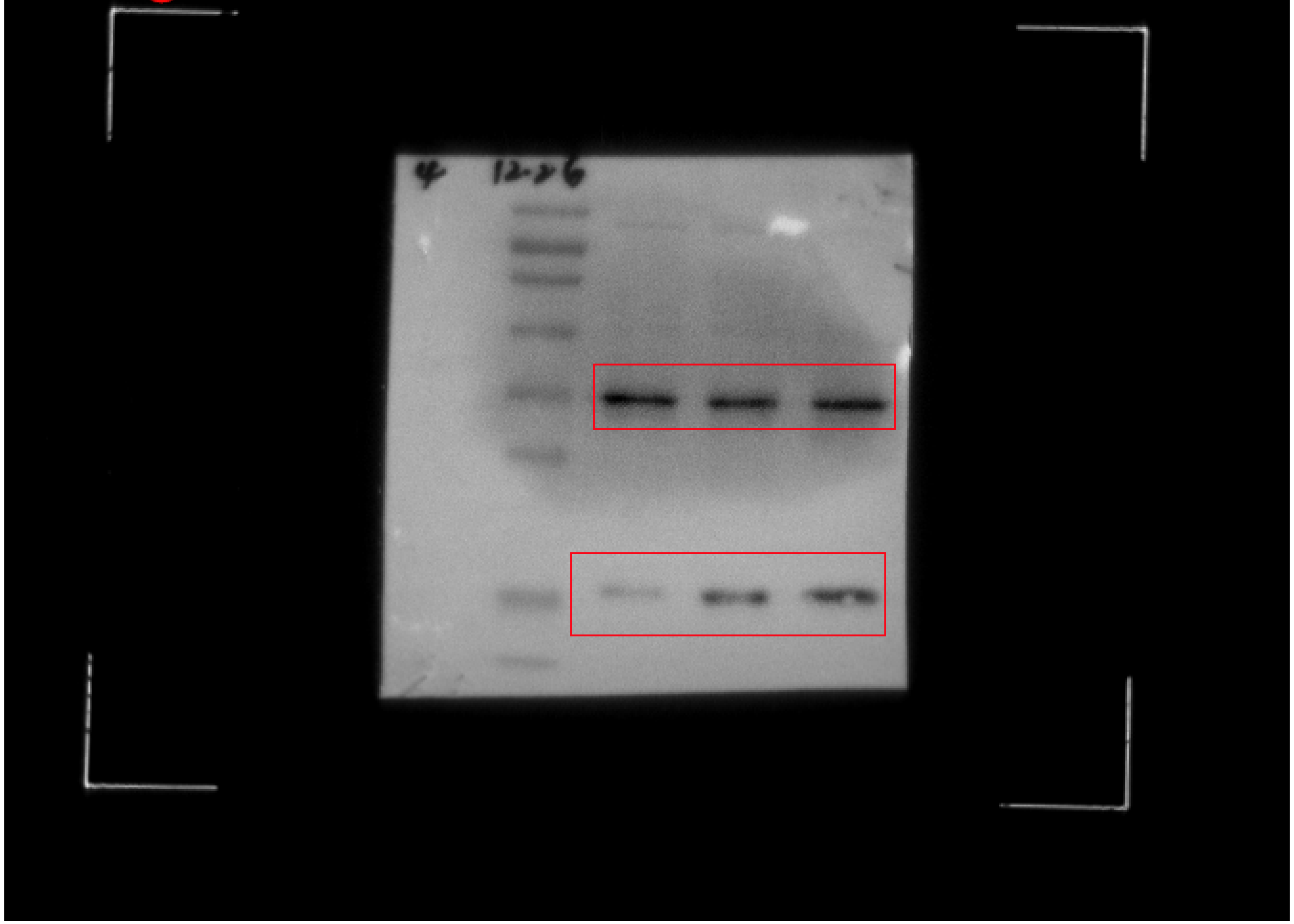

Fig 6C LN229 CC3 and Cleaved CC3

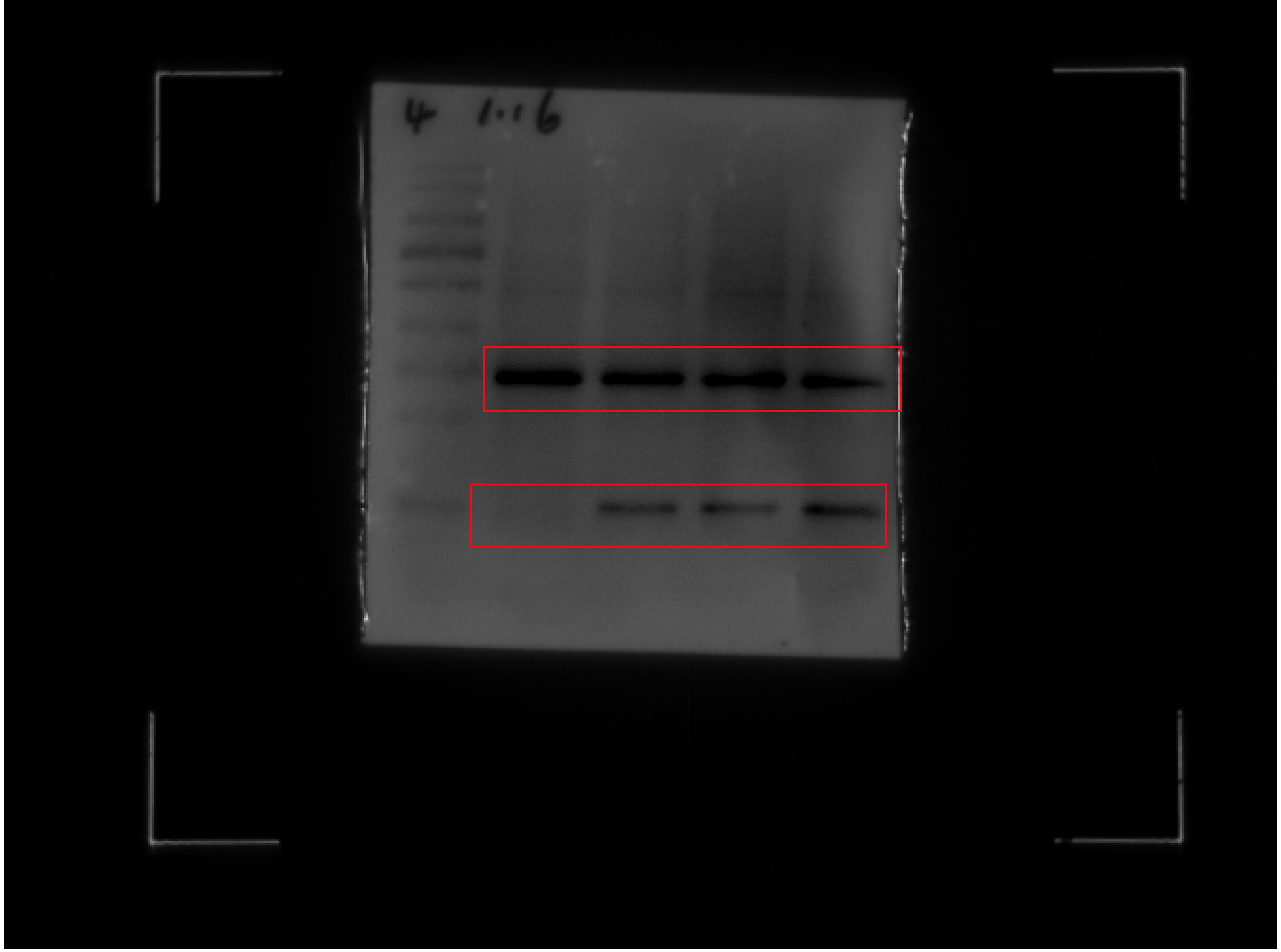

Fig 6C LN229 PARP and Cleaved PARP

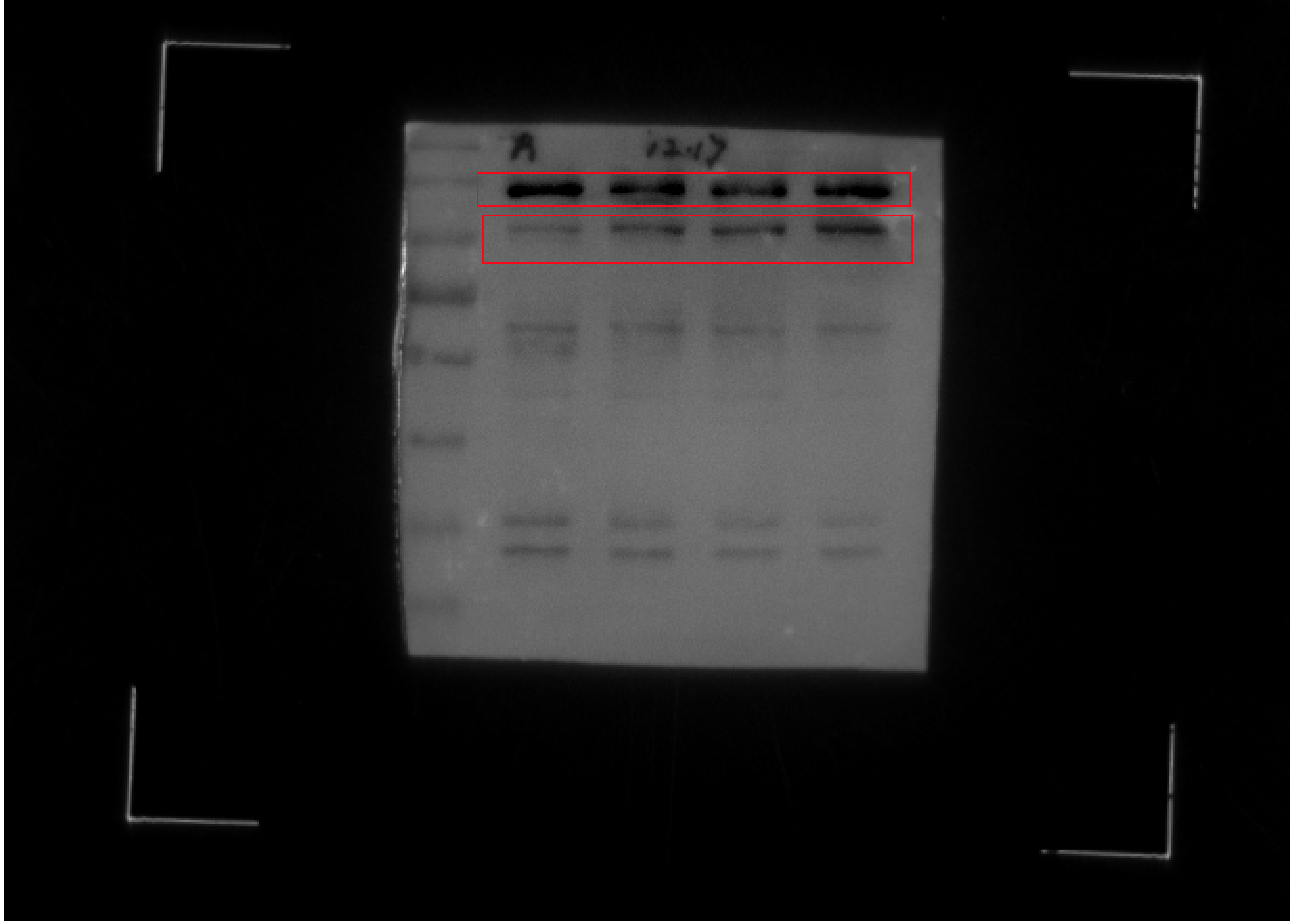

Fig 6C LN229 tubulin

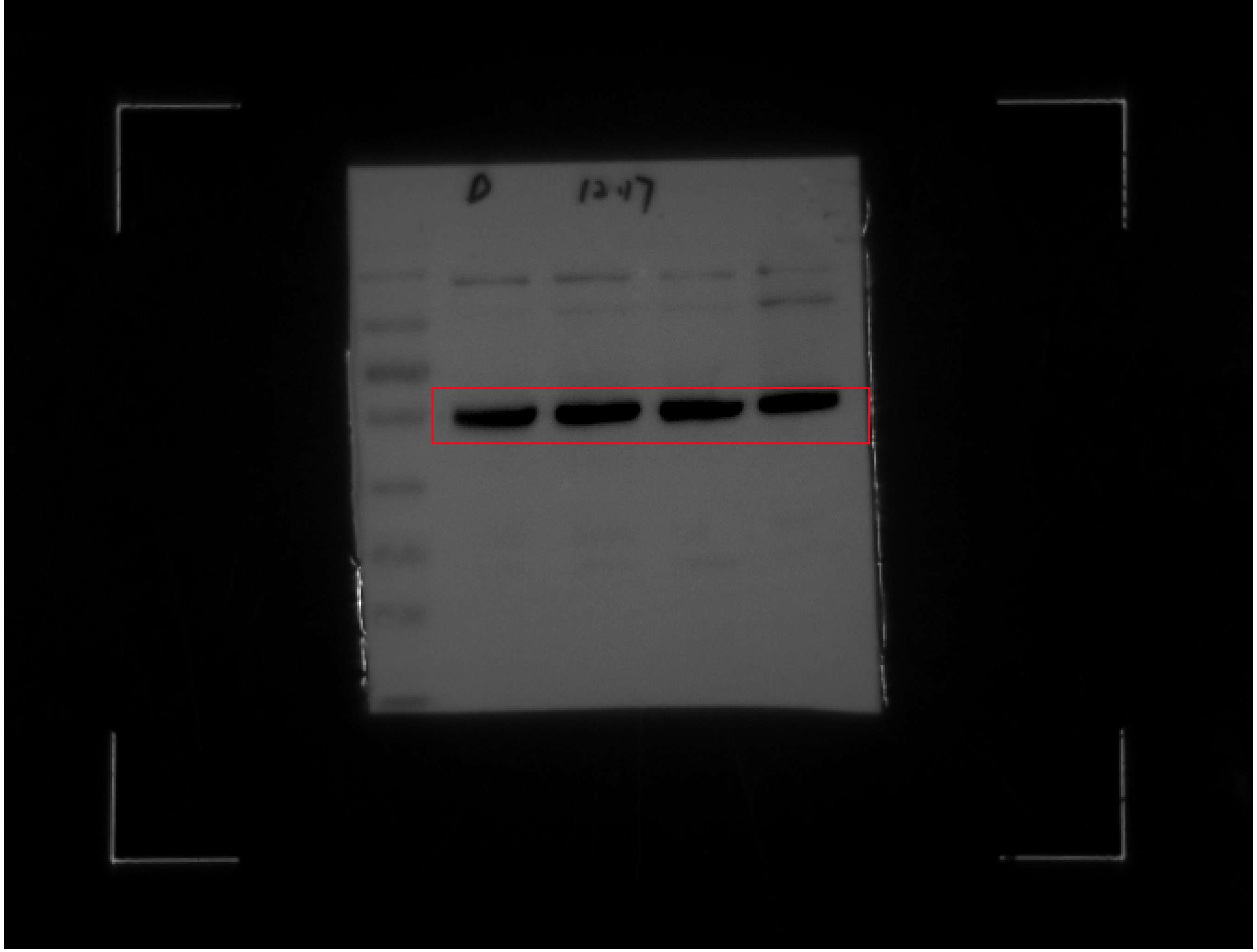

Fig 6C SHG141 CC3 and Cleaved CC3

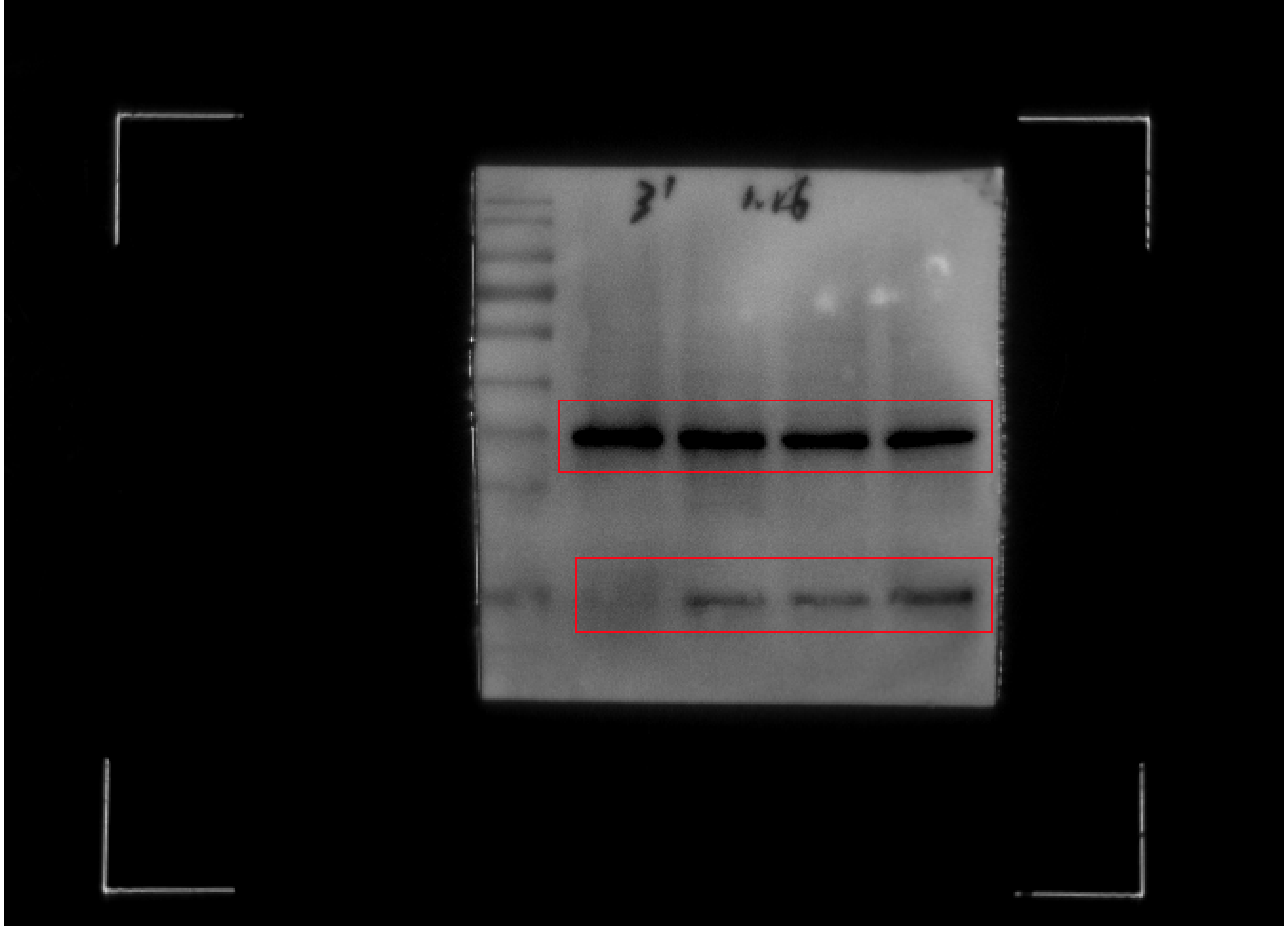

Fig 6C SHG141 PARP and Cleaved PARP

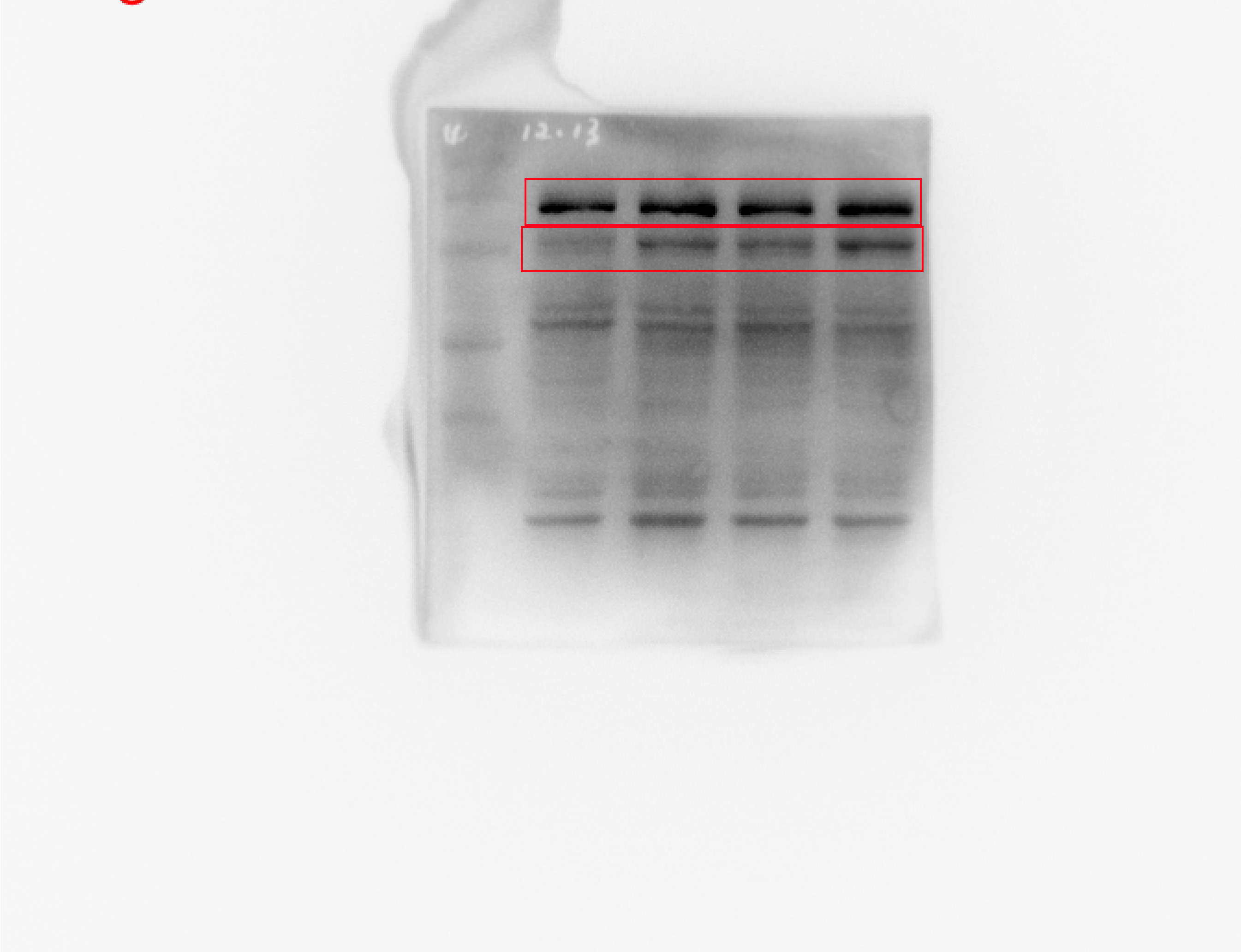

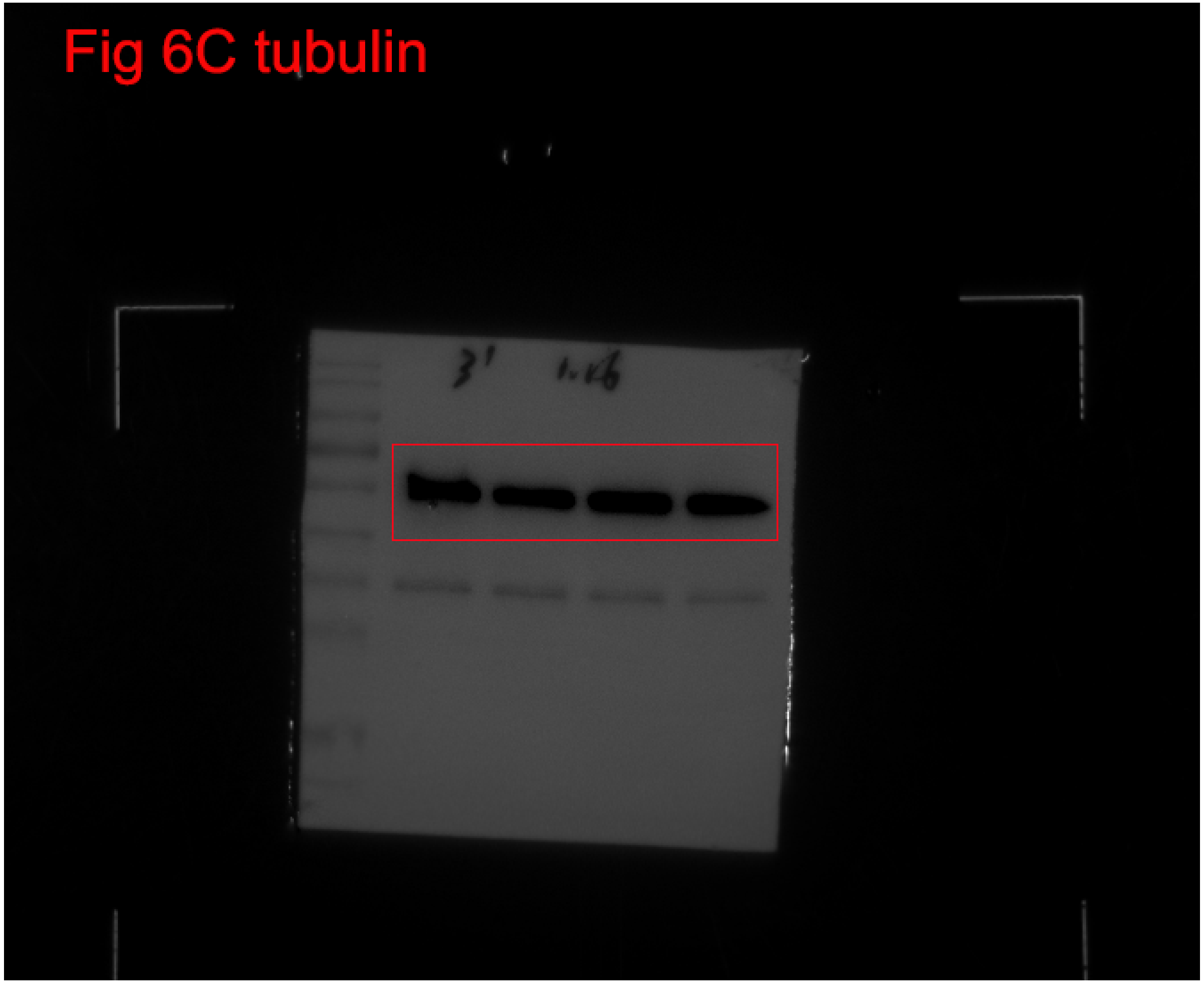
Fig 6C tubulin

Fig 6C Cleaved CC3

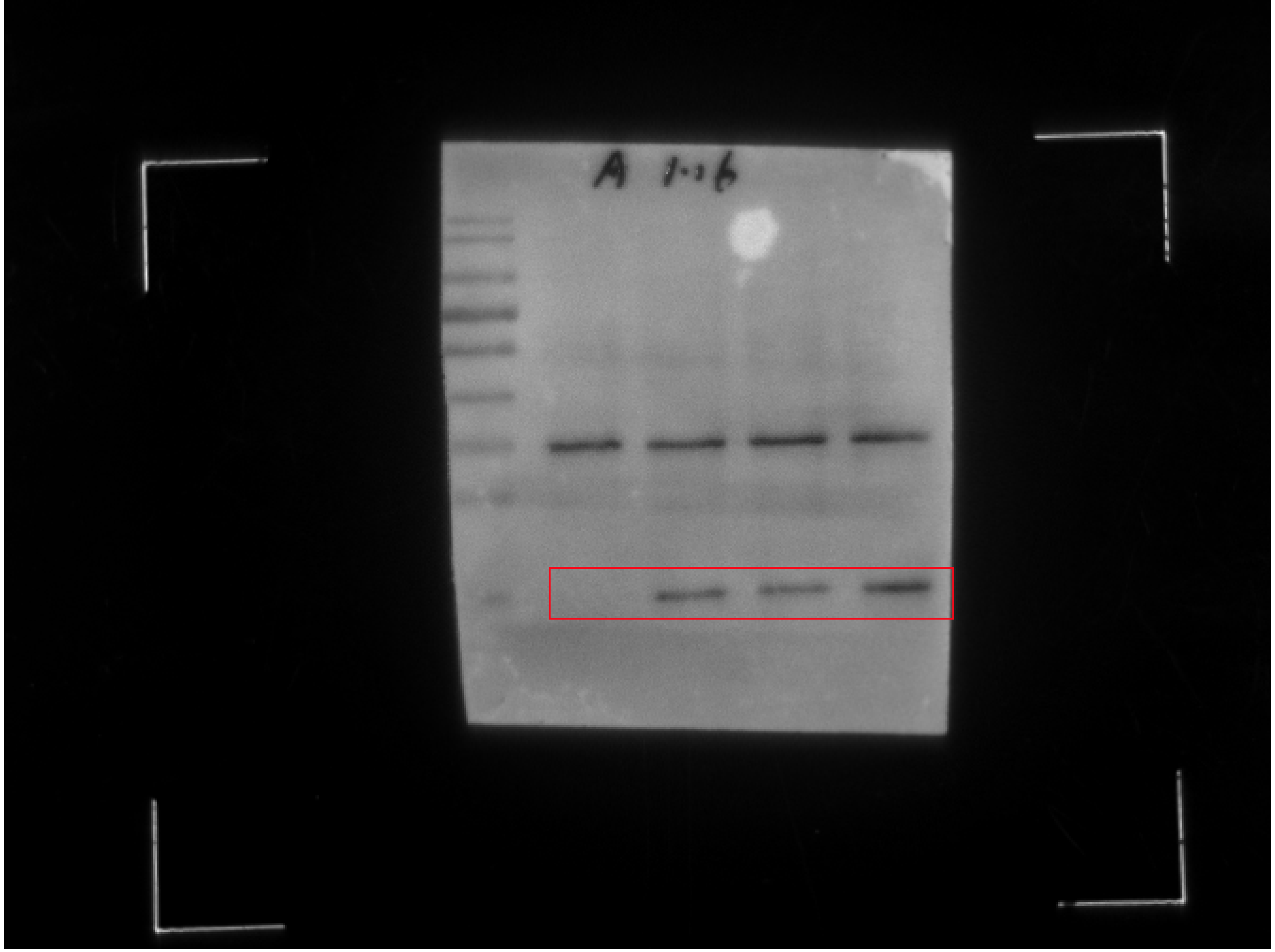

Fig 6C Cleaved PARP and PARP

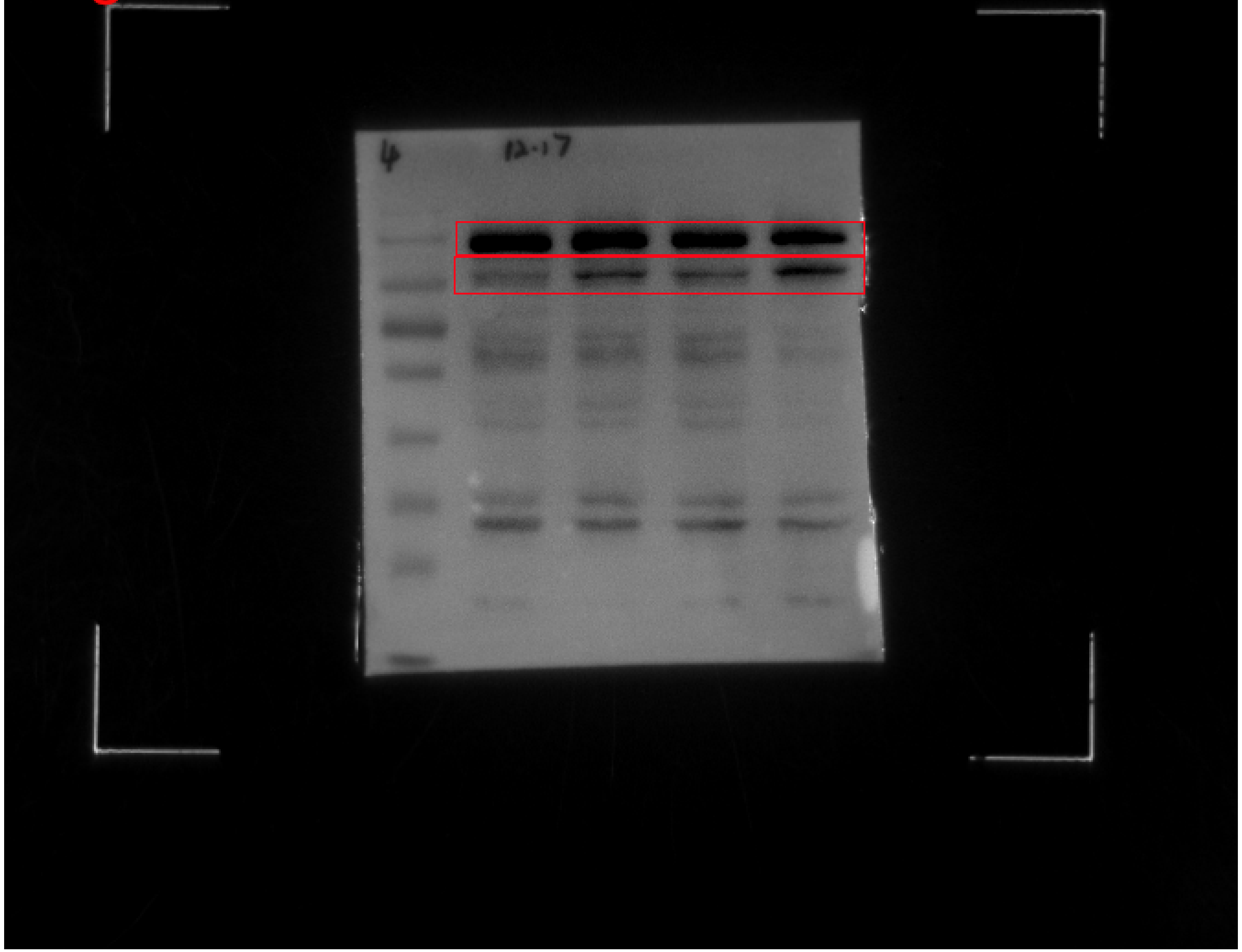

Fig 6C tubulin

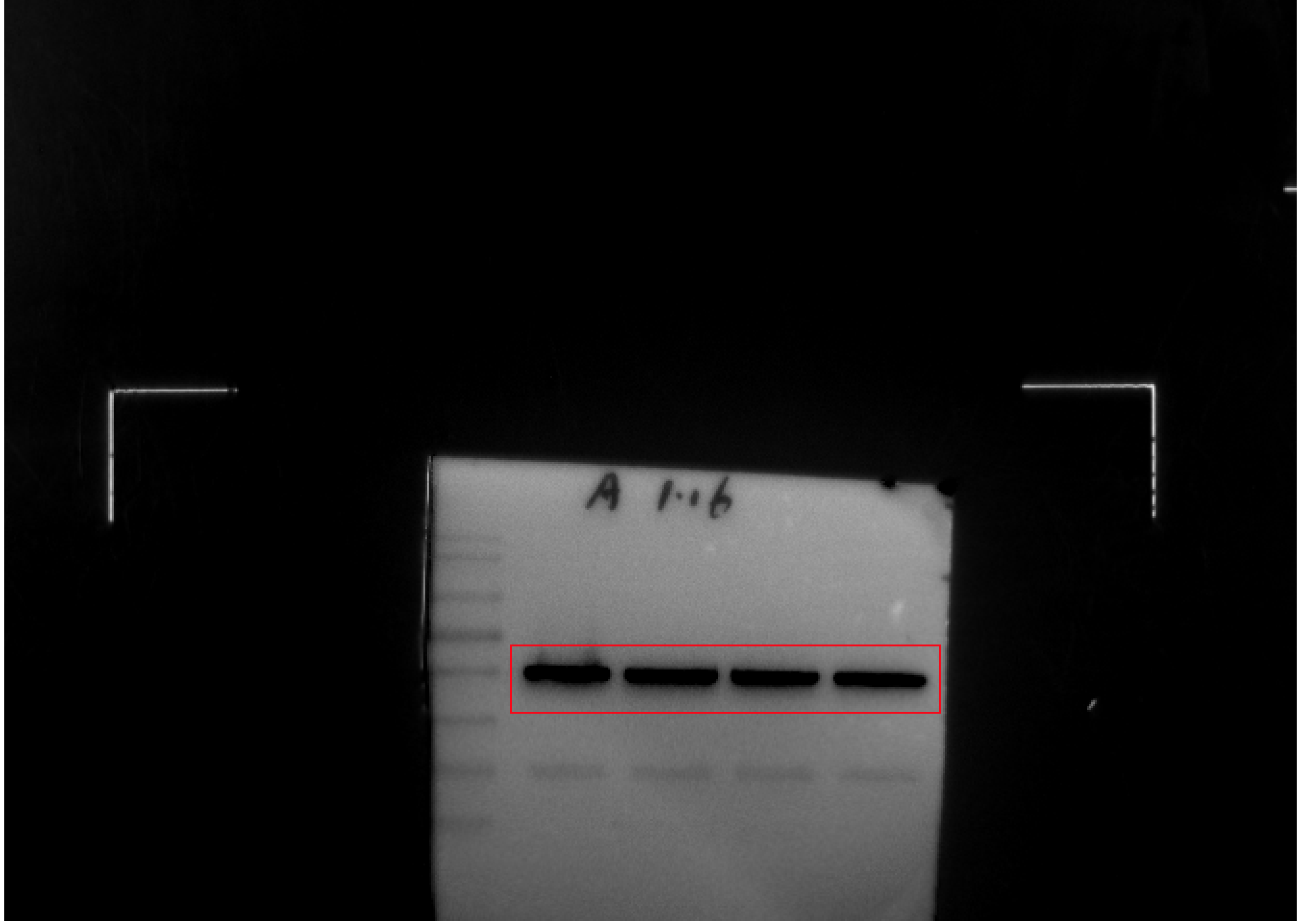

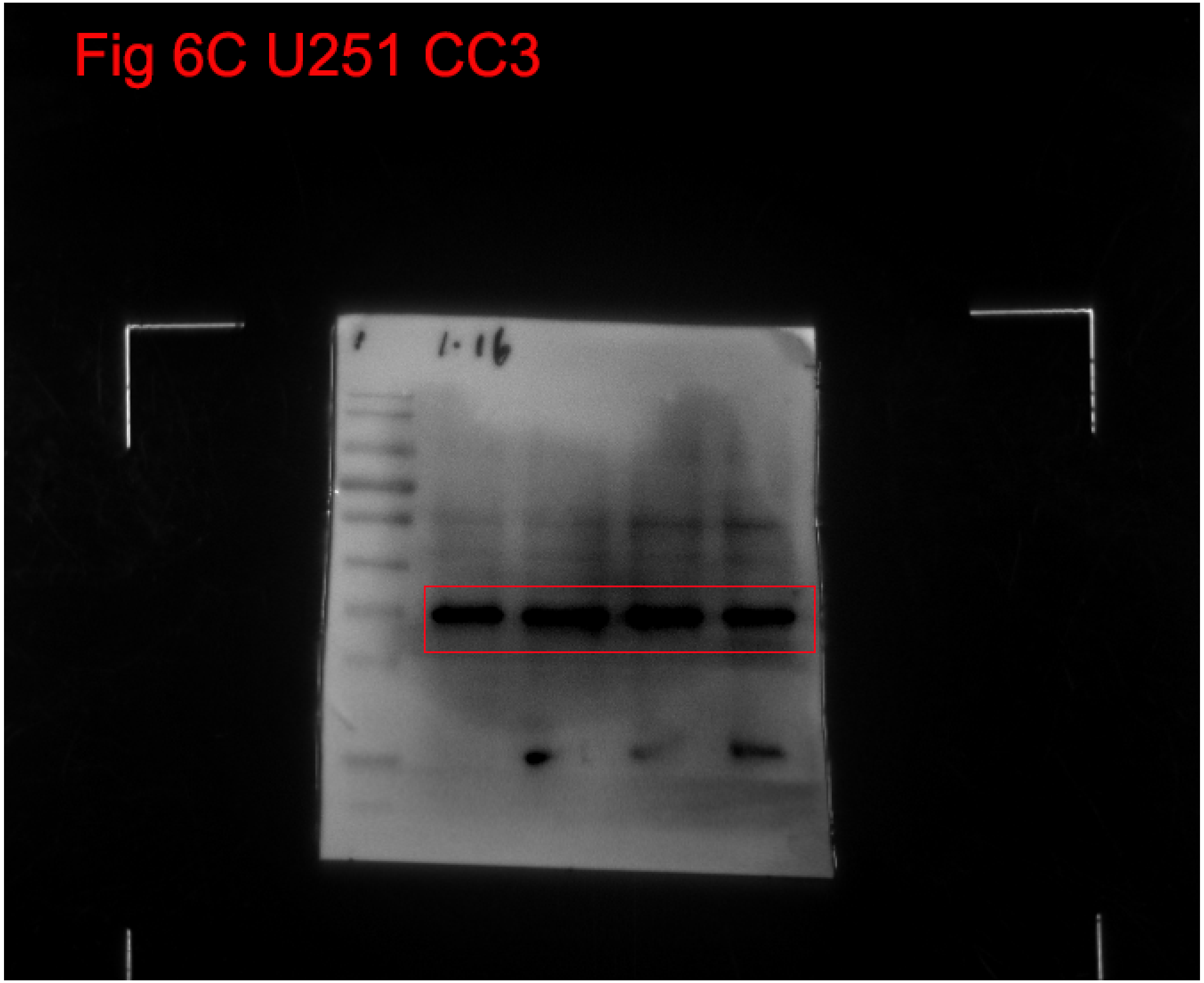
Fig 6C U251 CC3
1.16

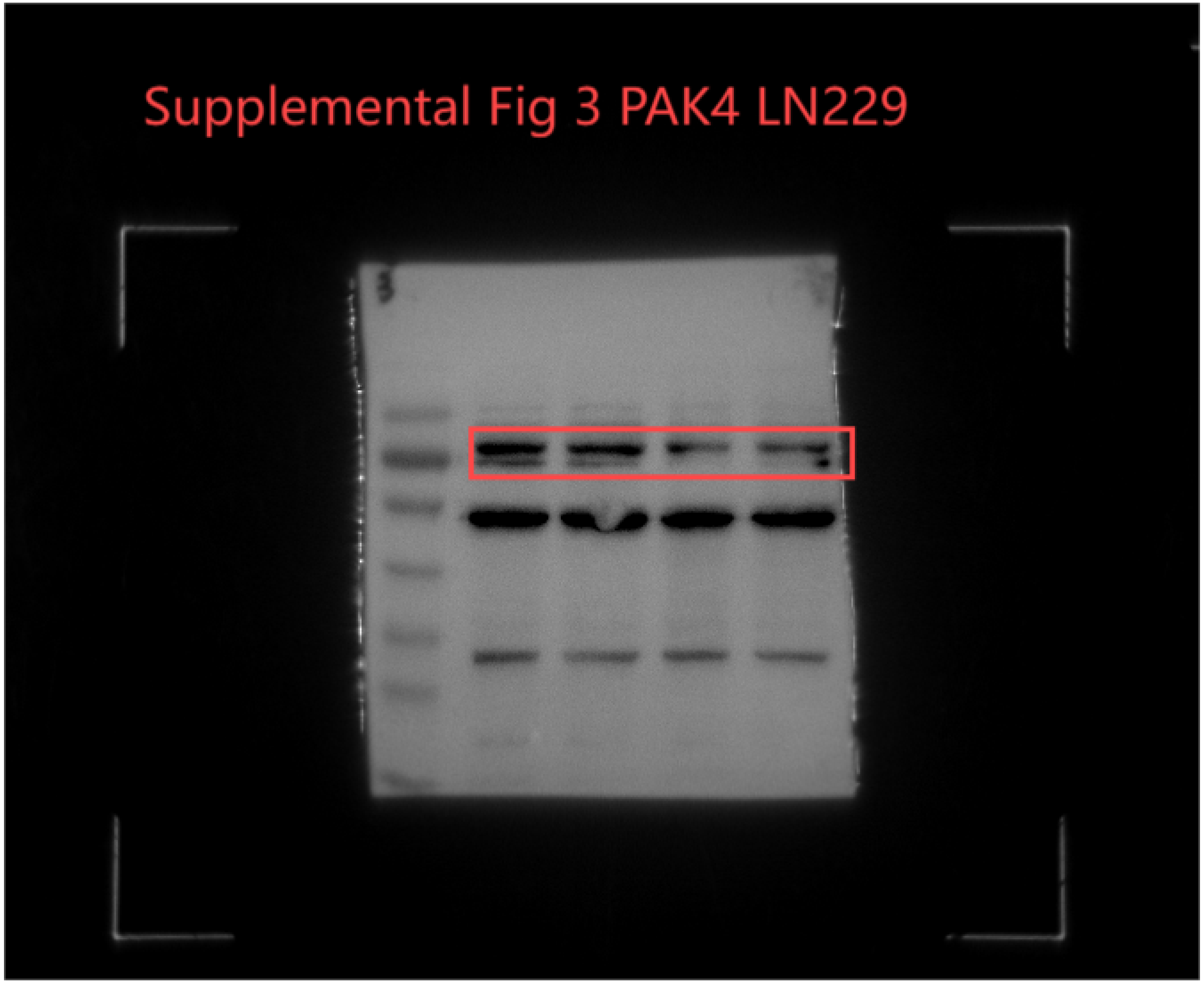
Supplemental Fig 3 PAK4 LN229

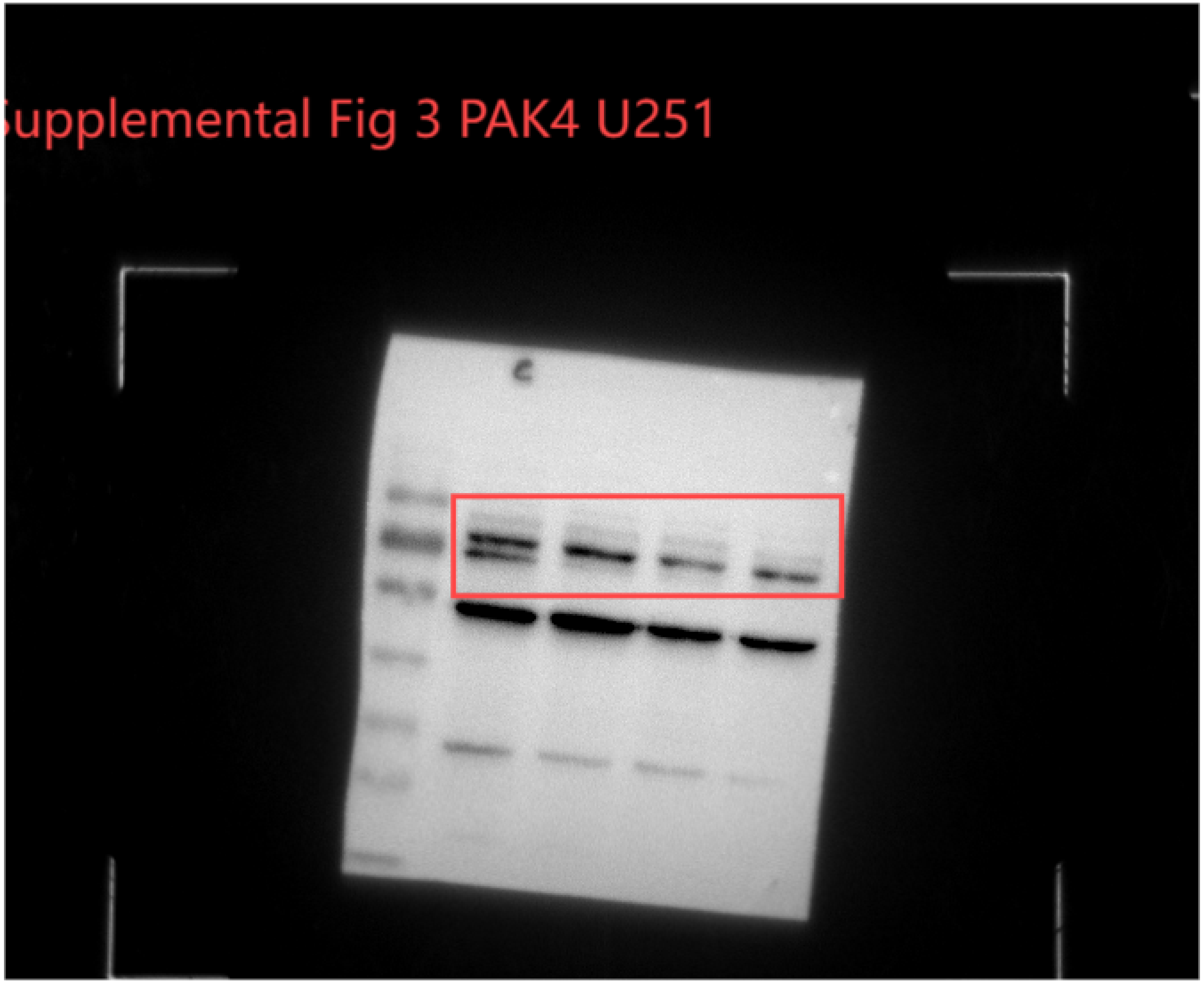
Supplemental Fig 3 PAK4 U251

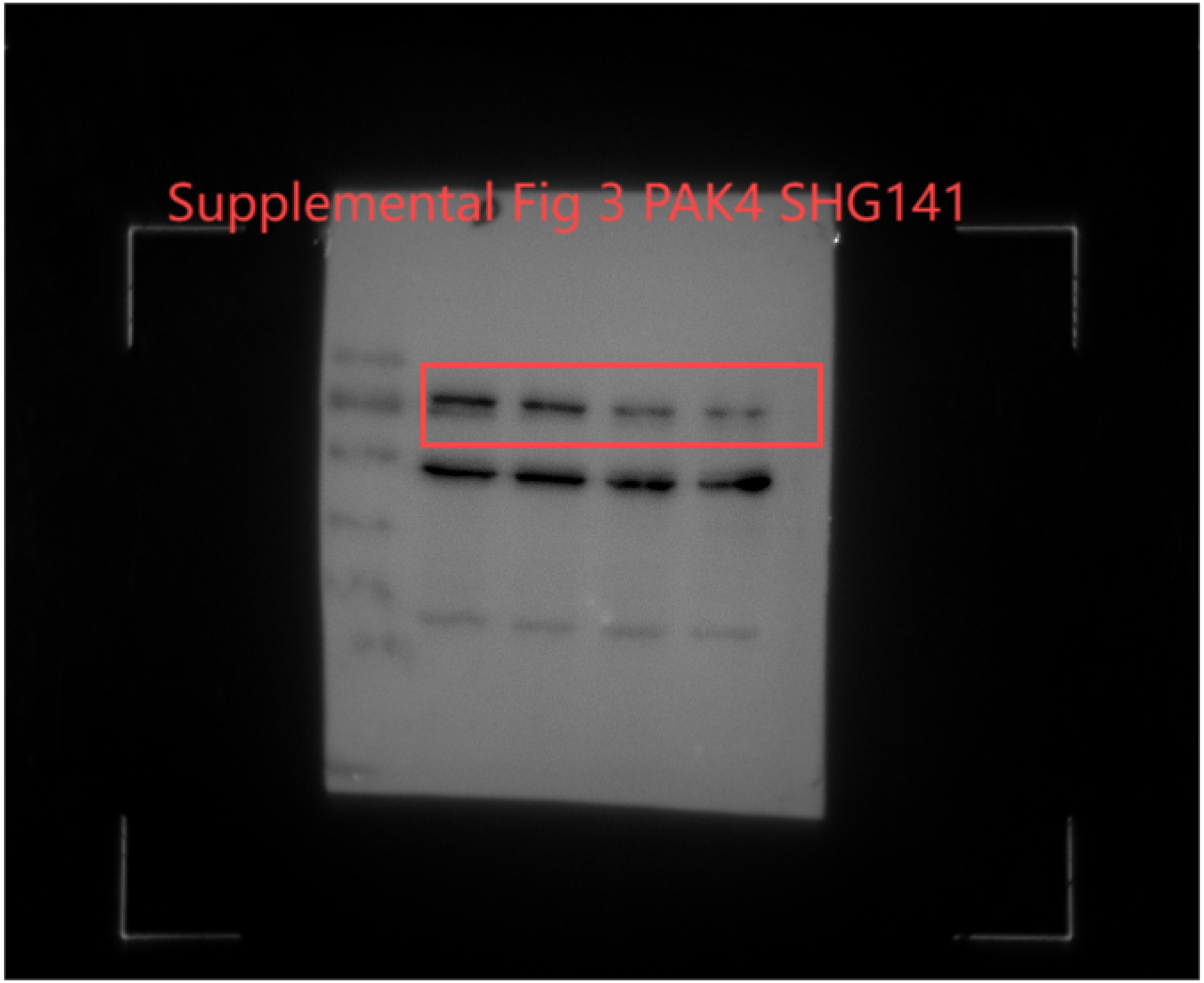
Supplemental Fig 3 PAK4 SHG141

Supplemental Fig 3 STAT3 LN229

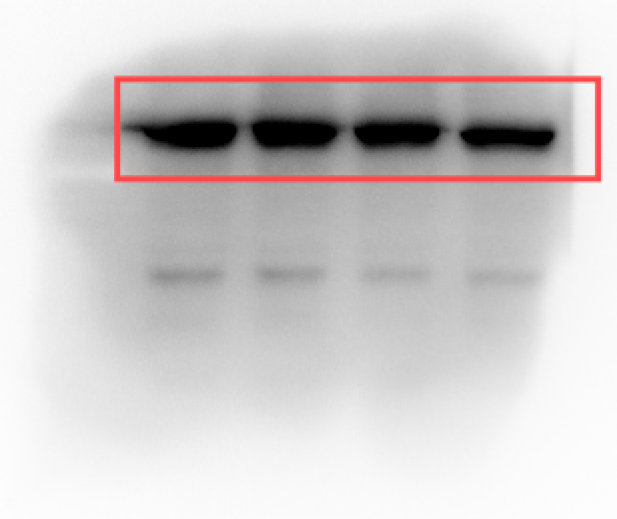

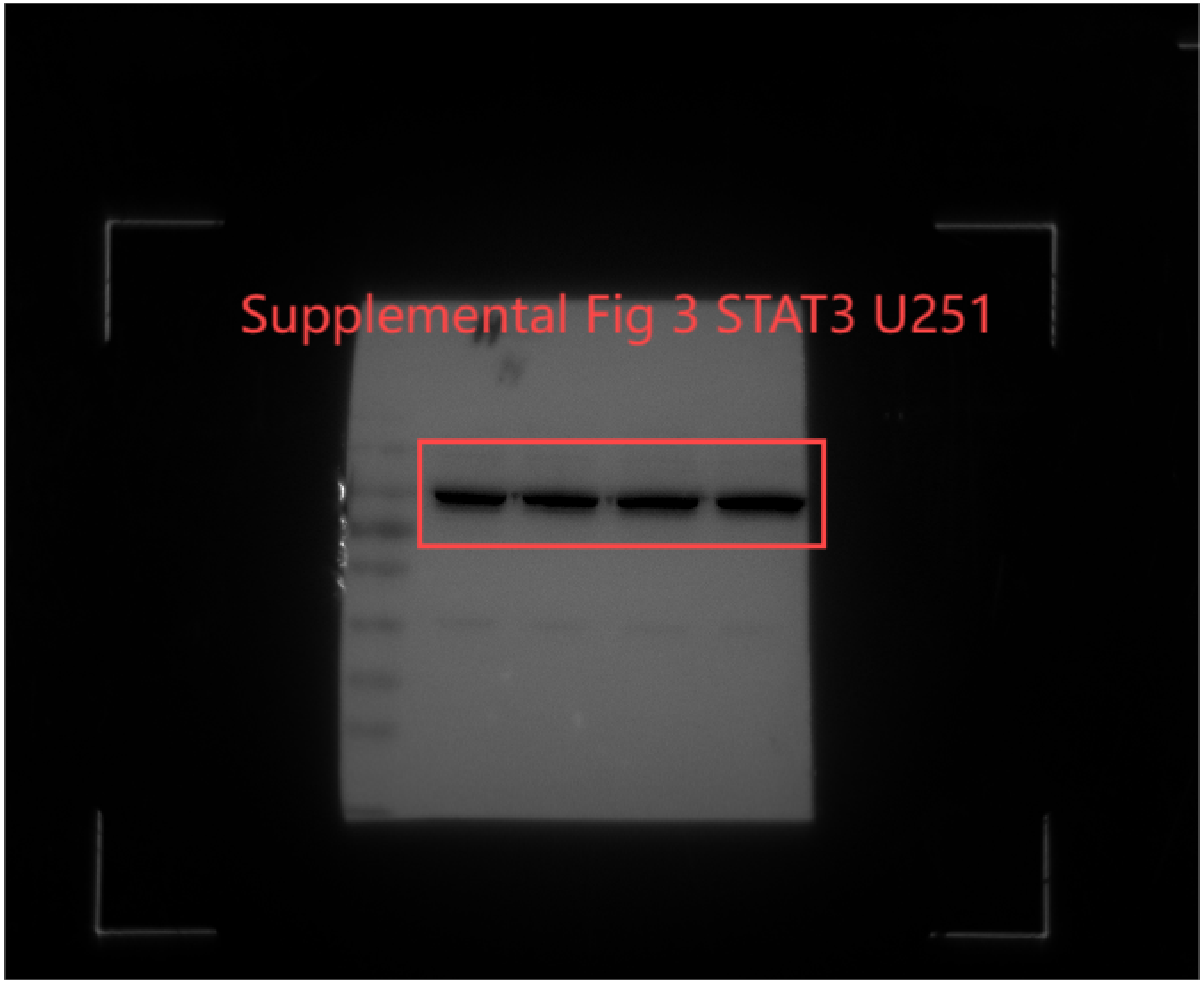
Supplemental Fig 3 STAT3 U251

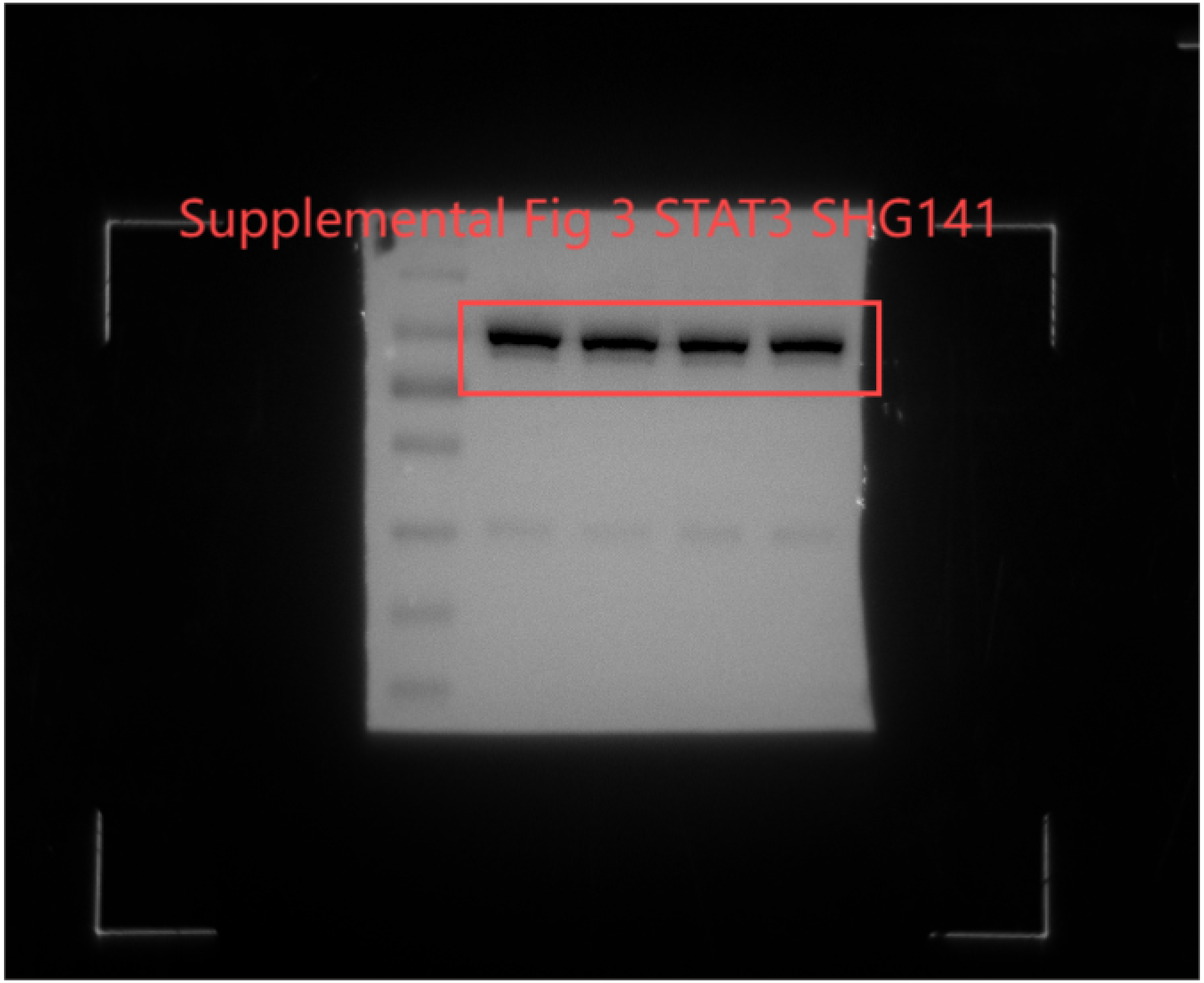
Supplemental Fig 3 STAT3 SHG141

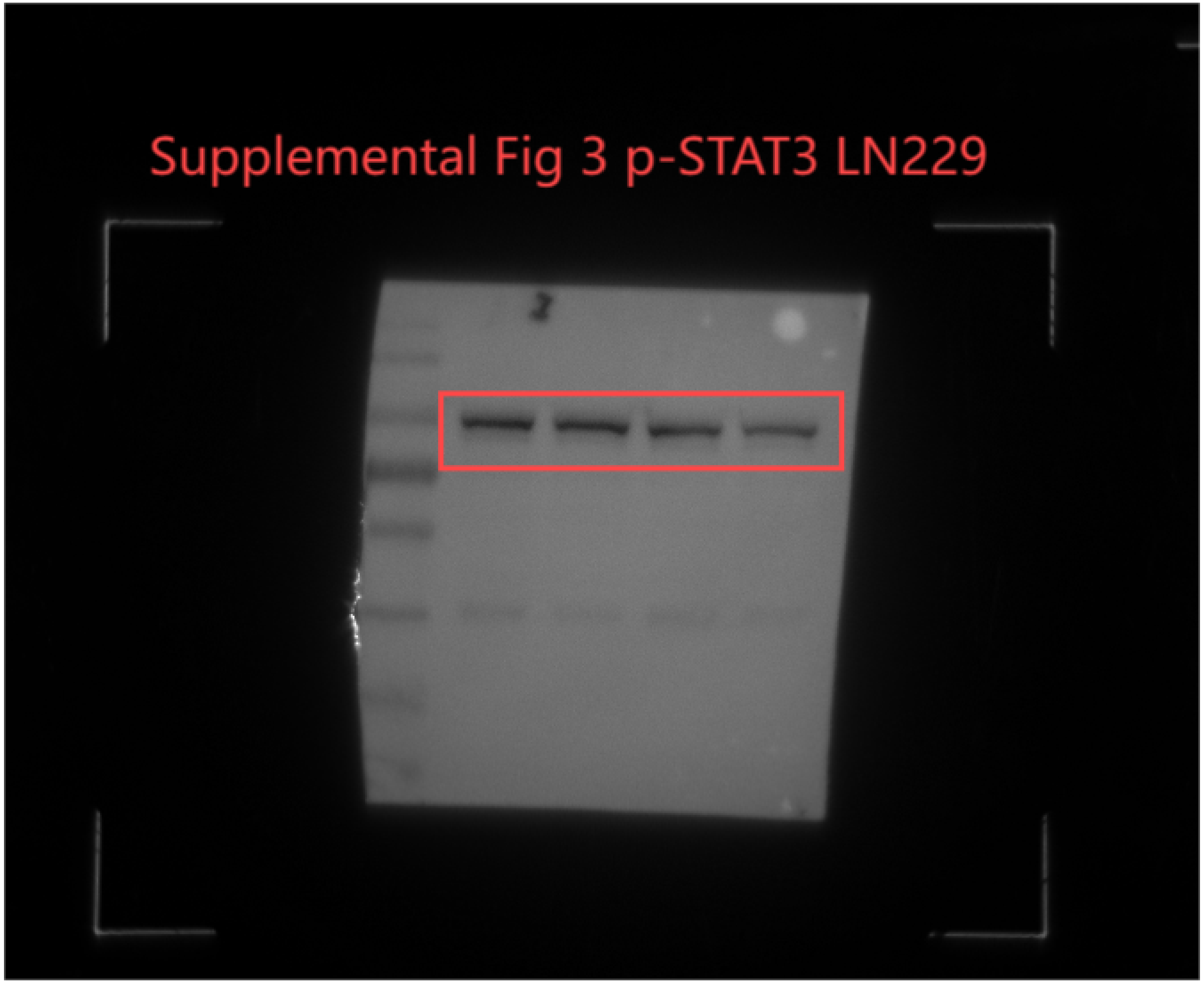
Supplemental Fig 3 p-STAT3 LN229

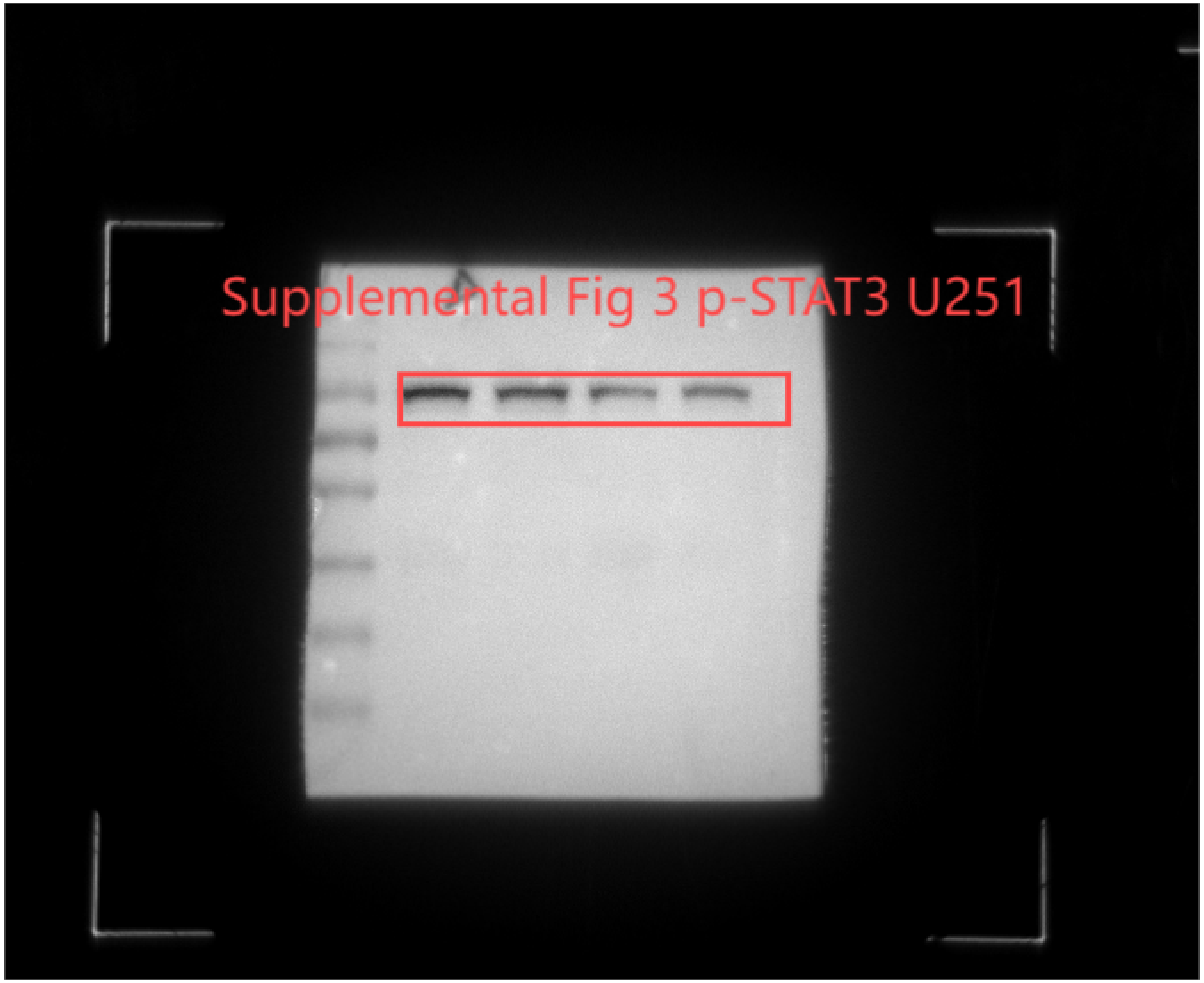
Supplemental Fig 3 p-STAT3 U251

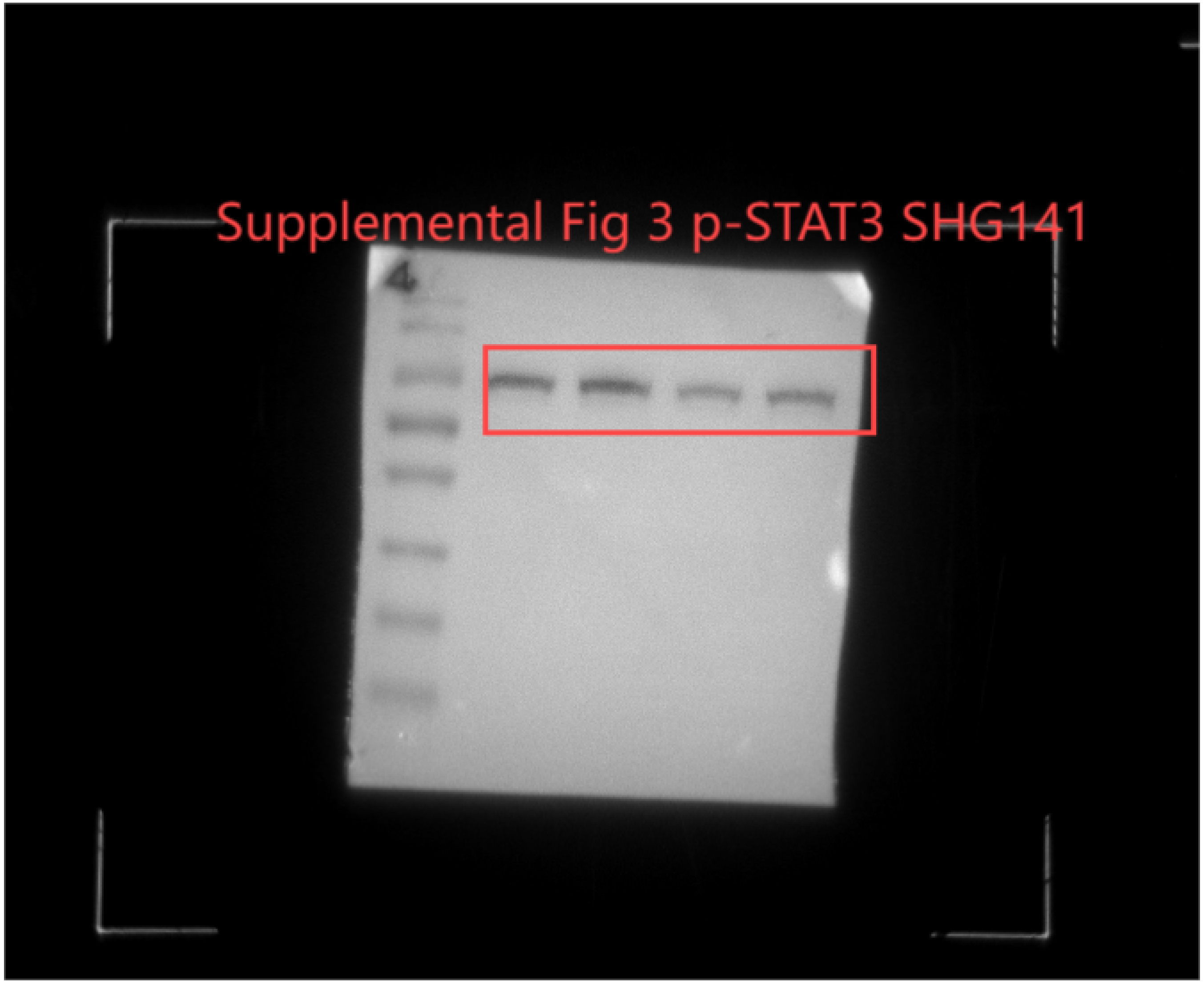
Supplemental Fig 3 p-STAT3 SHG141

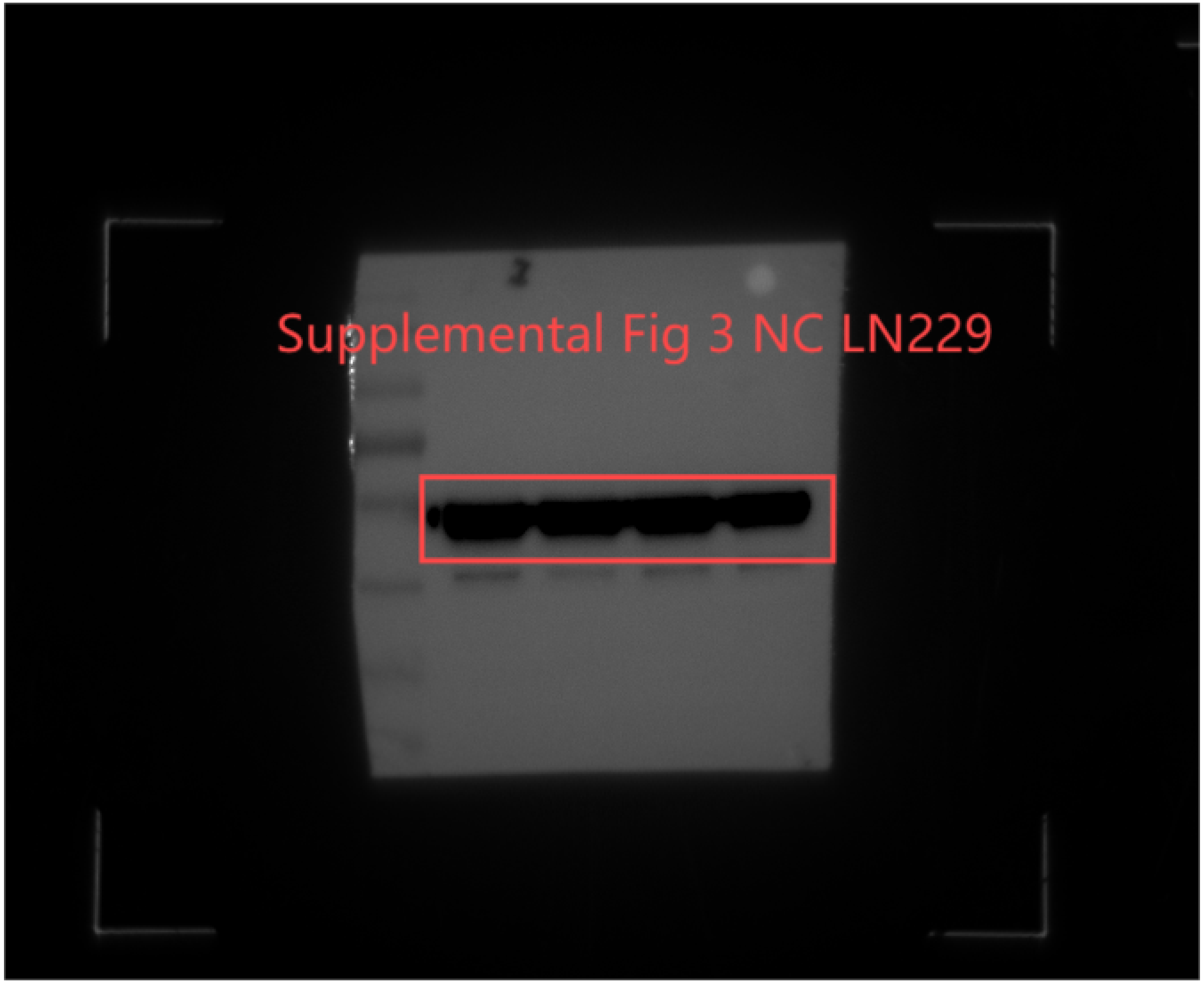
2
Supplemental Fig 3 NC LN229

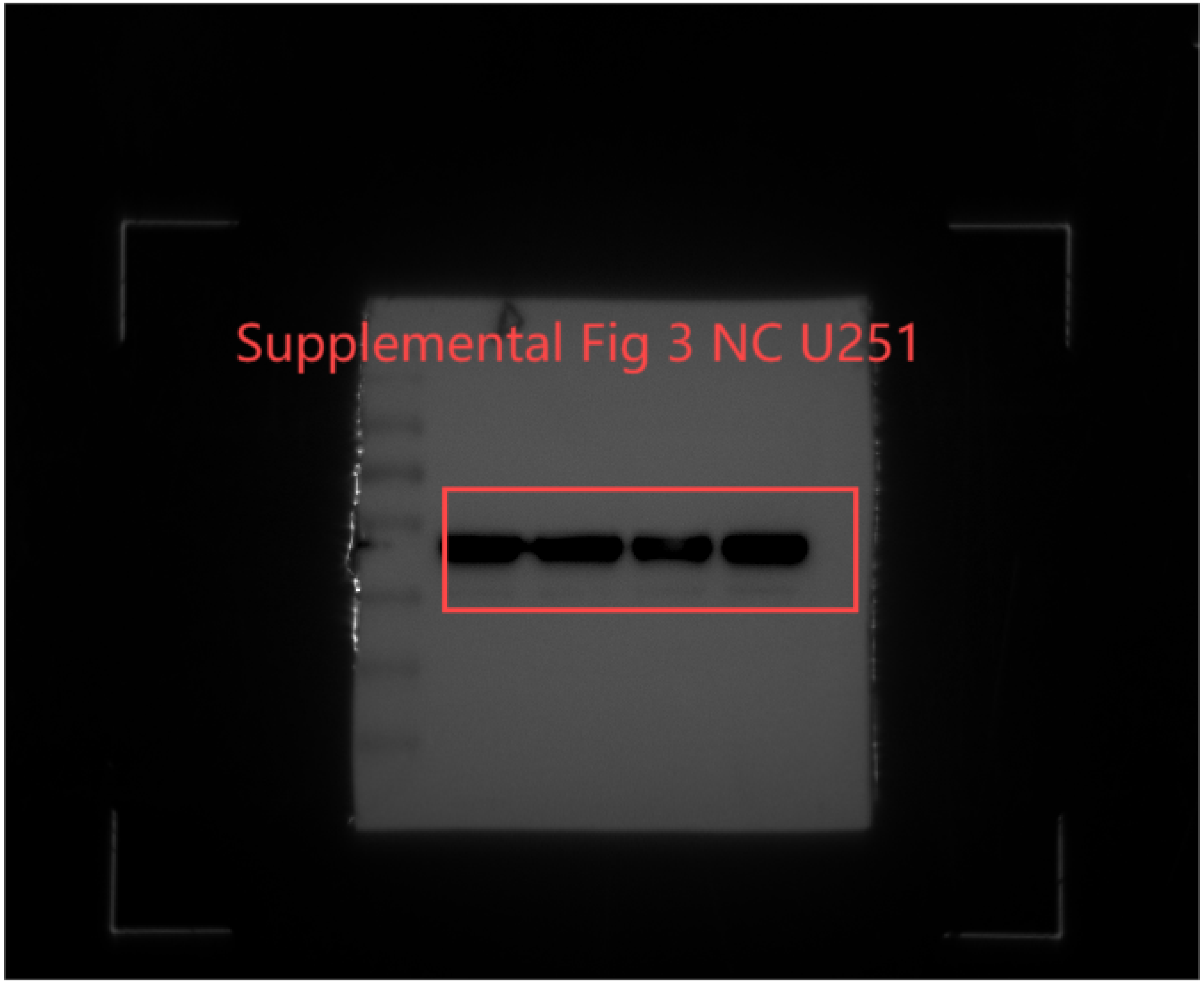
Supplemental Fig 3 NC U251

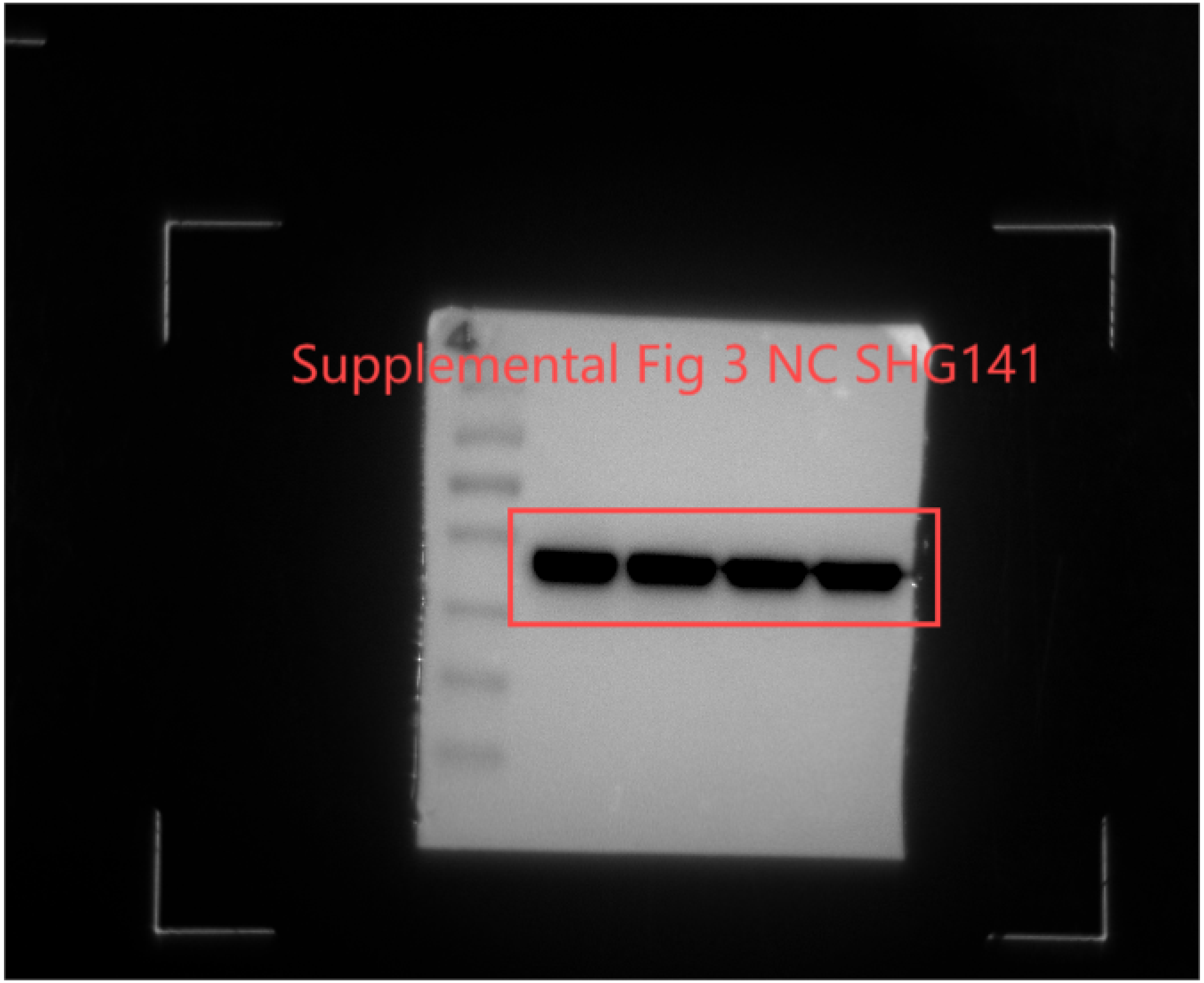
Supplemental Fig 3 NC SHG141
